# Foxo3 regulates cortical and medullary thymic epithelial cell homeostasis with implications in T cell development

**DOI:** 10.1038/s41419-024-06728-0

**Published:** 2024-05-21

**Authors:** Camila Ribeiro, Pedro Ferreirinha, Jonathan J. M. Landry, Fátima Macedo, Laura G. Sousa, Rute Pinto, Vladimir Benes, Nuno L. Alves

**Affiliations:** 1 i3S - Instituto de Investigação e Inovação em Saúde, Universidade do Porto, Porto, Portugal; 2 IBMC - Instituto de Biologia Molecular e Celular, Universidade do Porto, Porto, Portugal; 3 ICBAS - Instituto de Ciências Biomédicas Abel Salazar, Universidade do Porto, Porto, Portugal; 4 Genomics Core Facility, European Molecular Biology Laboratory, Heidelberg, Germany; 5 Departamento de Ciências Médicas, Universidade de Aveiro, Aveiro, Portugal

**Keywords:** Adaptive immunity, Central tolerance, Autoimmunity, T cells

## Abstract

Within the thymus, thymic epithelial cells (TECs) create dedicated microenvironments for T cell development and selection. Considering that TECs are sensitive to distinct pathophysiological conditions, uncovering the molecular elements that coordinate their thymopoietic role has important fundamental and clinical implications. Particularly, medullary thymic epithelial cells (mTECs) play a crucial role in central tolerance. Our previous studies, along with others, suggest that mTECs depend on molecular factors linked to genome-protecting pathways, but the precise mechanisms underlying their function remain unknown. These observations led us to examine the role of Foxo3, as it is expressed in TECs and involved in DNA damage response. Our findings show that mice with TEC-specific deletion of *Foxo3* (Foxo3^cKO^) displayed a disrupted mTEC compartment, with a more profound impact on the numbers of CCL21^+^ and thymic tuft mTEC^lo^ subsets. At the molecular level, Foxo3 controls distinct functional modules in the transcriptome of cTECs and mTECs under normal conditions, which includes the regulation of ribosomal biogenesis and DNA damage response, respectively. These changes in the TEC compartment resulted in a reduced total thymocyte cellularity and specific changes in regulatory T cell and iNKT cell development in the Foxo3^cKO^ thymus. Lastly, the thymic defects observed in adulthood correlated with mild signs of altered peripheral immunotolerance in aged Foxo3^cKO^ mice. Moreover, the deficiency in *Foxo3* moderately aggravated the autoimmune predisposition observed in *Aire*-deficient mice. Our findings highlight the importance of Foxo3 in preserving the homeostasis of TECs and in supporting their role in T cell development and tolerance.

## Introduction

Within the thymus, the development of immunologically competent and self-tolerant T cells relies on inductive signals provided by specialized microenvironments constituted by thymic epithelial cells (TECs) [1]. Notably, deficits in TEC function arise with age, infection and cancer cytoablative chemotherapies, resulting in reduced T cell production and skewed T cell receptor (TCR) repertoire. Such failures in TEC function contribute to poor T cell responses and immune surveillance or may also promote autoimmunity, thereby increasing morbidity and mortality in the elderly and in patients undergoing bone marrow transplantation or receiving anticancer treatments [2]. Thus, understanding the molecular mechanisms controlling TEC homeostasis is essential for developing novel therapeutic strategies that can enhance immune reconstitution or correct insufficient T cell responses.

The thymic epithelium is broadly subdivided into cortical (cTEC) and medullary (mTEC) subsets, which arise from common bipotent TEC progenitors (TEPs) [3]. While cTECs promote T cell lineage commitment and positive selection, mTECs regulate the elimination of self-reactive thymocytes via negative selection or their deviation into regulatory T cells. The crucial role of mTECs in tolerance induction is, in part, attributed to their ability to promiscuously express tissue-restricted antigens (TRAs) [3]. The complex molecular mechanisms governing mTEC function are under intense scrutiny and depend, to some extent, on the autoimmune regulator (Aire). Studies have suggested that Aire interacts with proteins involved in DNA damage response, leading to the induction of double-strand DNA breaks and facilitating the transcription of TRA-encoding genes [4]. Interestingly, we previously showed that p53, a well-known regulator of the DNA damage response, serves as a guardian of mTECs and their function in T cell development and self-tolerance [5]. These findings suggest the possibility that promiscuous gene expression of TRAs in mTECs leads to genomic instability, which requires protecting mechanisms to preserve the integrity of the medullary microenvironment. Yet, the pathways controlling the balance of the mTEC niche under physiological conditions are still not fully understood.

In this study, we sought to identify novel transcription factors involved in the DNA damage pathway, which might protect the genomic integrity of mTECs while maintaining their role in thymopoiesis. Particularly, we focused on investigating the role of forkhead box O 3 (Foxo3) in TECs. Foxo3 is a member of the Foxo family of transcriptions factors, which also includes Foxo1, Foxo4 and Foxo6. Foxo proteins have a pleiotropic role in several cellular processes, including survival, proliferation and metabolism [6]. In particular, the *Foxo3* gene has been implicated in increased human longevity and a reduced risk of age-related diseases. Foxo3 is believed to contribute to longevity by regulating responses to various stress signals, including cell cycle arrest, DNA damage and oxidative stress [7]. In this study we examine the link between Foxo3 and the homeostasis of the TEC microenvironment, and its role in T cell development.

## Results

### The deletion of *Foxo3* in TECs leads to a reduction in the mTEC compartment

The observation that p53 controls mTEC homeostasis prompted us to search for novel transcription factors involved in p53-mediated DNA damage response. To do so, we examined transcriptomic data sets from cTECs and mTECs of the 2-week-old thymus [5, 8] (Fig. S1A). Given the high expression of *Foxo3* in TECs, we sought to determine its role in TEC differentiation and function. To specifically delete *Foxo3* in TECs, we developed a Foxo3 conditional KO model by crossing mice carrying loxP-flanked *Foxo3* alleles (Foxo3^fl/fl^) [9] with mice expressing the Cre recombinase under the control of the *Foxn1* promoter (Foxn1^Cre^) [10]. Foxn1^Cre^Foxo3^fl/fl^ mice (Foxo3^cKO^) were born without any apparent abnormalities and showed a similar development to their control Foxo3^fl/fl^ littermates (Foxo3^Ctr^). PCR analysis of genomic DNA showed that the *Foxo3* floxed allele was deleted in TECs from Foxo3^cKO^ thymus, but not in thymocytes. Moreover, the *Foxo3* floxed allele was not deleted in TECs from Foxo3^Ctr^ thymus (Fig. S1B, C). These results suggest that Foxo3 expression is successfully inactivated in TECs from Foxo3^cKO^ mice.

**Fig. 1:**
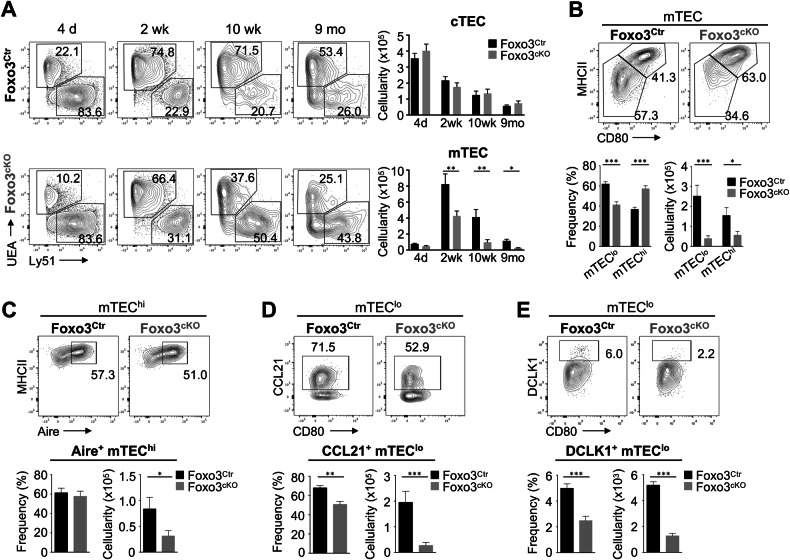
Foxo3 supports the size and functional diversity of the mTEC compartment. **A** Thymi were isolated from Foxo3^Ctr^ (Foxo3^fl/fl^) and Foxo3^cKO^ (Foxn1^Cre^:Foxo3^fl/fl^) mice at the indicated time points. TECs (CD45^-^EpCAM^+^) were analysed for cTEC (Ly51^+^) and mTEC (UEA^+^) composition. Bar graphs show absolute cell numbers. **B** mTEC^lo^ (MHCII^low^CD80^low^) and mTEC^hi^ (MHCII^high^CD80^high^) composition within total mTECs of the 10-week-old adult thymus. **C** Aire expression within mTEC^hi^. **D** CCL21 expression within mTEC^lo^. **E** Expression of tuft cell marker DCLK1 within mTEC^lo^. Bar graphs show absolute cell numbers and percentages. Data are representative of 2 or 3 independent experiments per time-point (*n* = 6National Natural Science Foundation of China (Grant No. 82372631). National Natural Science Foundation of China (Grant No. 82173024). Beijing Natural Science Foundation (Grant No.7212061)9 independent samples). All data are represented as mean ± SEM. d - days; wk - weeks; mo - months. *P < 0.05; **P < 0.01; ***P < 0.001.

We began by comparing the thymic epithelium composition of Foxo3^Ctr^ and Foxo3^cKO^ mice during key time points of postnatal life. Although unaltered in the postnatal day 4 thymus, the total number of TECs was significantly reduced in the 2- and 10-week-old Foxo3^cKO^ thymus (Fig. S1D). Despite moderate variations in the frequency of cTECs/mTECs, all major TEC subsets, including cTECs, mTEC^lo^, mTEC^hi^Aire^-^ and mTEC^hi^Aire^+^, were detected in the thymus of Foxo3^cKO^ at postnatal day 4 (Fig. 1A, Fig. S1E and data not shown). These results indicated that the first stages of TEC differentiation appeared to proceed normally in the absence of Foxo3. Notably, the number of mTECs, but not cTECs, became significantly reduced in the Foxo3^cKO^ thymus from 2-week-old onwards (Fig. 1A). Further analysis of MHCII^low^CD40^low^ (cTEC^lo^) and MHCII^hi^CD40^hi^ (cTEC^hi^) subsets showed no differences between Foxo3^Ctr^ and Foxo3^cKO^ in the adult thymus (Figure S1F). In contrast, the adult mTEC compartment of the Foxo3^cKO^ thymus showed marked alterations in the frequency of MHCII^low^CD80^low^ (mTEC^lo^) and MHCII^hi^CD80^hi^ (mTEC^hi^) subsets, leading to a more pronounced reduction in the numbers of the mTEC^lo^ population (Fig. 1B). We next examined the composition of functionally distinct subsets included within mTEC^hi^ (Aire^+^) and mTEC^lo^ (CCL21^+^ and thymic tuft cells) populations [3]. Although there were differences in the frequency of mTEC^hi^, the fraction of the Aire^+^ subset within mTEC^hi^ was comparable between Foxo3^Ctr^ and Foxo3^cKO^ groups (Fig. 1C). In contrast, concomitant to a reduction in the frequency of mTEC^lo^, the percentages of CCL21^+^ and tuft-like DCLK1^+^ cells within mTEC^lo^ were further diminished in the mutant thymus. Thus, despite the reduced cellularity of Aire^+^ cells, the deficiency in *Foxo3* led to a more significant decrease in the number of mTEC^lo^ subsets (Fig. 1D, E). The histological analysis of adult Foxo3^cKO^ thymi showed reduced overall thymic areas, while the cortex and medulla areas appeared to segregate normally. Despite the relative proportion of the medullary area to the total thymic area found in the Foxo3^cKO^ thymus being comparable to that in controls, its structural organization showed a reduced number of medullary islets (Fig. S1G). These results suggest that a Foxo3-dependent pathway is essential for maintaining normal homeostasis of the mTEC compartment.

### *Foxo3* deficiency induces changes in the transcriptome of cTECs and mTECs

**Fig. 2:**
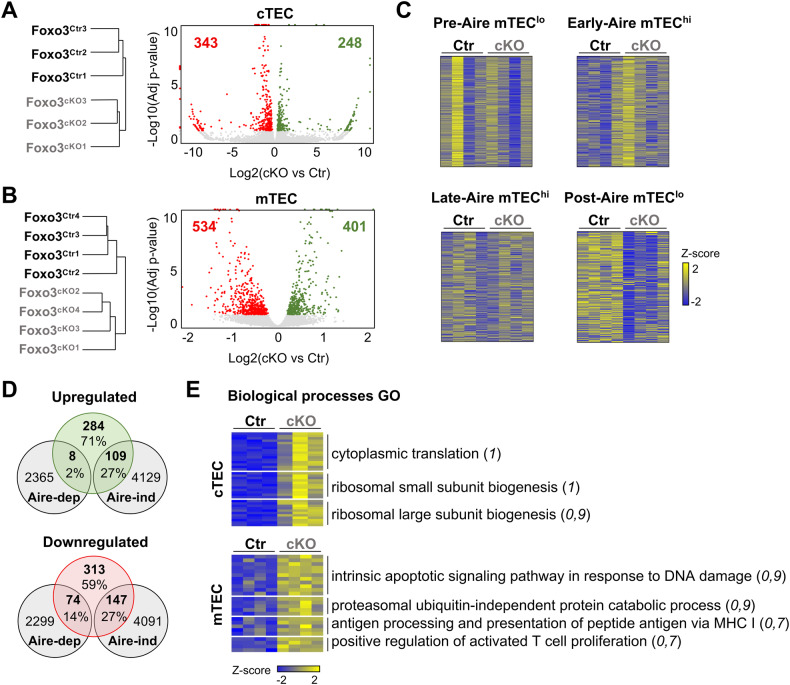
Foxo3^cKO^ TEC subsets display altered transcriptional signatures. **A**, **B** RNA-Seq analysis of FACS-sorted cTECs (**A**) and mTECs (**B**) purified from 6-week-old Foxo3^Ctr^ and Foxo3^cKO^ mice. Hierarchical clustering of all biologically independent samples (left). Volcano plot depicting dzownregulated and upregulated DE genes in Foxo3^cKO^ versus Foxo3^Ctr^ samples (right). In volcano plots, off-scale points were collapsed to the nearest axis. **C** Relative expression level of genes specifically upregulated in pre-, early-, late- and post-Aire mTECs (data obtained from [11]) in Foxo3^Ctr^ and Foxo3^cKO^ mTECs. **D** Venn diagrams depict the frequency and number of Foxo3^cKO^ mTEC upregulated (top) and downregulated (bottom) genes classified as Aire-dependent (Aire-dep) or Aire-independent (Aire-ind) tissue-restricted antigens, as defined in [8]. **E** Representation of the most enriched biological processes obtained by Gene Ontology (GO) analysis of upregulated genes in cTECs and mTECs. Terms represented show statistical significance with a marginal posterior probability estimate that is > 0.7 (enclosed within parentheses). Heatmap rows represent the relative expression level of the differentially expressed genes for each biological process. Their identity is specified in Supplementary Table 6 (for cTECs) and Supplementary Table 8 (for mTECs).

To elucidate the mechanism underlying the role of Foxo3 in TEC maintenance, we performed bulk RNA-sequencing (RNA-Seq) analysis and compared the transcriptomes of cTECs and mTECs isolated from 6-week-old Foxo3^Ctr^ and Foxo3^cKO^ mice. We selected this age to ensure the mTEC phenotype unfolds in Foxo3^cKO^ mice and, consequently, to capture potential transcriptional changes, all while obtaining a sufficient number of cTECs and mTECs for analysis. Hierarchical clustering of all biological samples showed that cTECs and mTECs separated primarily according to cell types (data not shown). The quantification of reads covering exon 2 of *Foxo3* was markedly reduced in Foxo3^cKO^-derived TEC subsets, confirming the specific inactivation of the gene in mutant mice (Fig. S2A). Next, we compared the impact of *Foxo3* deficiency in the transcriptome of cTECs and mTECs. Approximately 600 genes for cTECs and 900 genes for mTECs showed statistically significant differences, which segregated control and mutant samples in the Principal Component 1 (PC1) (Fig. 2A, B, Figure S2B, Supplementary Tables 1–4). There was a small overlap in the identity of differentially expressed genes (DEGs) in cTECs and mTECs, suggesting that *Foxo3* deficiency has specific effects on the transcriptome of each cell population (Fig. S2C). Given the functional interplay between p53 and Foxo3 in the DNA damage response [6, 7], and our previous observations that p53 controls a broad molecular network of the mTEC transcriptome, including *Foxo3* [5], we compared the overlap between statistically significant DEGs affected by *Trp53* and *Foxo3* deficiency in cTEC and mTEC subsets. The deficiency in *Trp53* had a limited impact on the cTEC transcriptome compared to the effects of *Foxo3* deficiency (Fig. S2D) [5]. In contrast, the influence of *Trp53* deletion was broader than that of *Foxo3* in mTECs. Yet, only approximately 46% (435/935) of DEGs in Foxo3^cKO^ mTECs were commonly affected by *Trp53* deficiency, indicating that Foxo3 and p53 control the expression of common and specific targets in mTECs (Fig. S2D). Despite the changes imposed by *Foxo3* deficiency in cTECs, the expression of particular genes associated with cTEC-dependent functions (migration, commitment, selection) showed no difference between Foxo3^Ctr^ and Foxo3^cKO^ cTECs (Fig. S2E). On the contrary, the expression of particular genes linked to mature mTECs, such as *Aire* and *CD80*, was increased in mutant mTECs (Fig. S2F). These changes may be attributed to the RNA-Seq analysis being done in total mTECs, which show an increased frequency of mTEC^hi^ in the Foxo3^cKO^ thymus (as shown in Fig. 1).

We began by examining whether *Foxo3* deficiency caused alterations in transcriptional signatures of mTEC subsets defined by their history of Aire expression. We isolated the genetic signatures of pre-, early-, late-, and post-Aire mTECs [11], and examined the variation in the expression of the top 200 enriched genes within each cluster between control and mutant mTECs. While gene sets associated with pre-, early- and late-Aire cells lacked a specific pattern, the genetic signature of the post-Aire subset was downregulated in Foxo3^cKO^ mTECs (Fig. 2C). Similar complementary analysis of mTEC I-IV [12] populations revealed that the genetic signature of mTEC IV, which includes Post-Aire cells, was similarly downregulated in mutant mTECs (Fig. S3A). Recent findings indicated that Post-Aire cells harbour additional heterogeneity, consisting of cells that have genetic programs resembling those of extra-thymic cells, such as tuft cells, keratinocytes, ionocytes, enterocytes and more [13, 14]. We examined how *Foxo3* deficiency affected the genetic signatures of these mTEC mimetics [13]. Although our analysis was conducted on bulk mTEC populations and these specialized subsets are rare within mTECs, we found that gene sets, associated with Tuft- and Neuroendocrine-like cells were downregulated, whereas the ones linked to Microfold-like cells appeared slightly enriched in Foxo3^cKO^ mTECs (Fig. S3B). Moreover, we obtained a list of Aire-dependent and Aire-independent TRAs genes, identified through statistical comparison of their expression on wild type (WT) and Aire knockout (Aire^KO^) mTECs [8], and determined their presence among the DEGs of Foxo3^cKO^ mTECs. Downregulated DEGs included 41% of TRAs (14% Aire-dependent and 27% Aire-independent). Conversely, upregulated DEGs contained 29% TRAs (2% Aire-dependent and 27% Aire-independent) (Fig. 2D). These results suggested that *Foxo3* deficiency mostly affected the representation of Aire-independent TRAs.

To further dissect the molecular role of Foxo3 in TECs, we performed gene ontology (GO) analysis on cTEC and mTEC DEGs. GO analysis in downregulated DEGs in mutant cTECs or mTECs yielded no significant term enrichment. In contrast, upregulated DEGs in mutant cTECs showed enrichment in processes linked with ribosomes and their biogenesis (Fig. 2E, Supplementary Tables 5, 6). Notably, GO analysis of upregulated DEGs in Foxo3^cKO^ mTECs revealed enrichment in the intrinsic apoptotic signalling pathway, protein degradation, MHC I antigen presentation and processes that promote T cell activation (Fig. 2E, Supplementary Tables 7, 8). These results indicate that Foxo3 has a pervasive but diverse impact in the transcriptional programs of cTECs and mTECs.

### *Foxo3* deficiency alters the DNA damage response in mTECs

**Fig. 3:**
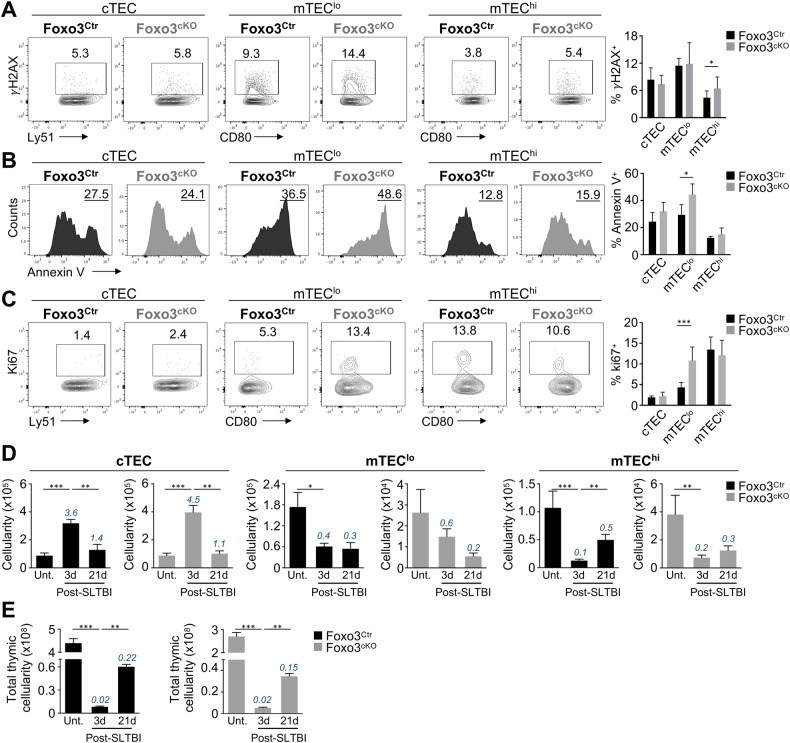
Foxo3^cKO^ mTECs present an impaired DNA damage response. **A**–**C** Frequency of *γ*H2AX^+^ cells (**A**), Annexin V^+^ cells in 7-AAD^-^ (**B**) and Ki67^+^ cells (**C**) in cTECs, mTEC^lo^ and mTEC^hi^ in Foxo3^Ctr^ and Foxo3^cKO^ adult thymus. Data are representative of 3 independent experiments (*n* = 7 to 9 independent samples) and represented as mean ± SD. **D**, **E** 10-week-old Foxo3^Ctr^ and Foxo3^cKO^ mice were subjected to sublethal total-body irradiation (SLTBI) and analysed at day 3 and day 21 post-irradiation. 10-week-old Foxo3^Ctr^ and Foxo3^cKO^ untreated mice (Unt.) were also analysed. The cellularity of cTECs, mTEC^lo^ and mTEC^hi^ (**D**) and total thymic cellularity (**E**) were determined at the indicated time-points. Numbers inserted above day 3 and day 21 columns depict the normalized recovery index of each population (average fold change relative to the corresponding untreated cellularity, which was set as 1). Data are representative of 2 or 3 independent experiments (*n* = 6–9 independent samples) and represented as mean ± SEM. *P < 0.05; **P < 0.01; ***P < 0.001.

Given the recognized role of Foxo3 in DNA damage response, we were particularly intrigued by the fact that the most significant augmented pathway in Foxo3^cKO^ mTECs was apoptosis in response to DNA damage. Concordantly, the analysis of the expression of Bcl-2 family members showed a statistically significant upregulation of some BH3-only and BAX/BAK-like genes in Foxo3^cKO^ mTECs. A similar trend was observed in the remaining pro-apoptotic genes, whereas the levels of their pro-survival counterparts were unaltered in mutant mTECs (Figure S3C). Moreover, assessment of double-strand DNA breaks and apoptosis showed an increase in the frequency of γ-H2AX in mTEC^hi^, and a higher frequency of annexin V^+^ cells in mTEC^lo^ of the Foxo3^cKO^ thymus (Fig. 3A, B). Due to the role of Foxo3 in promoting cell cycle arrest after DNA damage [15], we also analysed the proliferative rate of TECs and observed a higher proportion of Ki67^+^ Foxo3^cKO^ mTEC^lo^ (Fig. 3C). This observation was consistent with an increase in the genetic signature of recently identified proliferating mTECs, also termed transit-amplifying cells [16, 17], in mutant mTECs (Fig. S3D). To further determine whether Foxo3^cKO^ mTECs possess an impaired DNA damage response, we examined the regenerative capacity of mutant TECs after acute damage induced by ionizing radiation. Analysis of cTEC recovery on days 3 and 21 post-radiation showed a comparable dynamic between the control and mutant thymus. Despite showing numerical differences at steady state (untreated), the numbers of mTEC^lo^ and mTEC^hi^ significantly decreased by day 3 post-radiation in the control and mutant thymus (Fig. 3D; Fig. S4A). The high sensitivity of mTECs to radiation-induced damage was consistent with previous observations [5, 18, 19]. Yet, the radiation-induced decline was less pronounced in the Foxo3^cKO^ thymus, suggesting that deficiency in Foxo3 may confer some short-term resistance to apoptosis provoked by acute radiation (Fig. 3D; Fig. S4A). In both groups, mTEC^lo^ showed no significant regeneration by day 21 after treatment. Although the number of mTEC^hi^ was comparable by day 3 in both groups, their recovery was affected in Foxo3^cKO^ thymus by day 21 (Fig. 3D; Fig. S4A). Furthermore, despite an equivalent level of depletion in thymocyte cellularity by day 3 in both groups, the recovery was also affected in the Foxo3^cKO^ thymus by day 21 post-treatment (Fig. 3E; Fig. S4B). These results suggest that the regenerative response upon radiation-induced damage is inhibited in the Foxo3^cKO^ thymus.

### TEC-specific deletion of *Foxo3* affects the development of regulatory T cells and iNKT cells

**Fig. 4:**
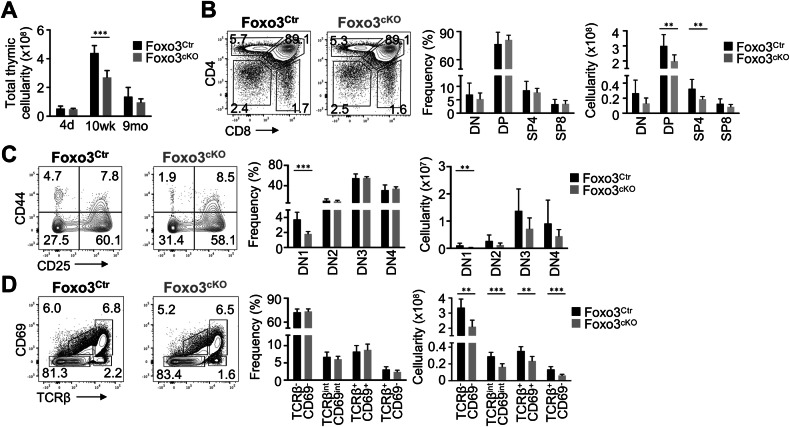
Thymopoiesis in Foxo3^cKO^ mice. **A** Total thymic cellularity at the indicated time points. **B** CD4 and CD8 expression on total thymocytes in the 10-week-old thymus of Foxo3^Ctr^ and Foxo3^cKO^ mice. **C** CD44 and CD25 expression on Lin^-^ CD4^-^ CD8^-^ DN thymocytes. **D** CD69 and TCRβ expression on total thymocytes. Bar graphs show absolute cell numbers and percentages. Data are representative of 2 to 4 independent experiments (*n* = 6–12 independent samples). All data are represented as mean ± SD. *P < 0.05; **P < 0.01; ***P < 0.001.

We next proceeded to examine how the described cellular and molecular alterations within the TEC microenvironments influence T cell development in the Foxo3^cKO^ thymus. While slightly similar at postnatal day 4, the number of thymocytes was notably reduced in the 10-week-old mutant thymus. These differences became however attenuated in the 9-month-old thymus (Fig. 4A). In the adult thymus, TEC-specific deletion of *Foxo3* did not lead to abnormalities in the frequency of the main thymocyte subsets, such as double-negative (DN), double-positive (DP), and CD4 and CD8 single-positive (SP) cells (Fig. 4B). The composition of immature DN subsets (DN1, CD25^−^CD44^+^; DN2, CD25^+^CD44^+^; DN3, CD25^+^CD44^−^; DN4, CD25^−^CD44^−^), gated on lineage negative (Lin^-^) markers (CD4, CD8, NK1.1; CD11b, CD11c, CD19, TCRγδ, Ter-119, GR1), was also similar in the mutant thymus, except for a significant reduction in the frequency and numbers of DN1 cells (Fig. 4C). Additionally, the frequency of DP thymocytes initiating selection (TCRβ^int^CD69^int^), post-positive selection SP thymocytes (TCRβ^hi^CD69^hi^) and mature SP thymocytes (TCRβ^hi^CD69^-^) [20] was similar in Foxo3^Ctr^ and Foxo3^cKO^ thymus. Still, their numbers were diminished as a result of the decreased total thymocyte cellularity in the Foxo3^cKO^ thymus (Fig. 4D).

**Fig. 5:**
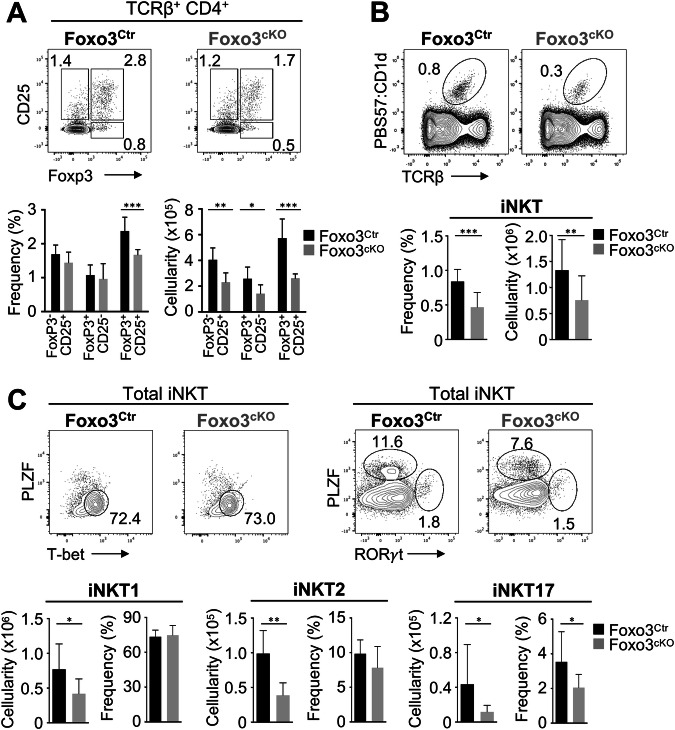
Foxo3^cKO^ mice exhibit compromised regulatory T cell and iNKT cell development. **A** CD25 and Foxp3 expression on TCRβ^+^ CD4^+^ thymocytes of 10-week-old thymus of Foxo3^Ctr^ and Foxo3^cKO^ mice. **B** Expression of TCRβ and reactivity with PBS57-loaded CD1d tetramer in total thymocytes. **C** Detection of T-bet^+^ iNKT1, PLZF^+^ iNKT2 and ROR*γ*t^+^ iNKT17 in total thymic iNKTs. Bar graphs show absolute cell numbers and percentages. Data are representative of 2 to 4 independent experiments (*n* = 6–12 independent samples). All data are represented as mean ± SD. *P < 0.05; **P < 0.01; ***P < 0.001.

Subsequently, we determined whether the more significant changes in mTEC numbers of the Foxo3^cKO^ thymus could directly impact mTEC-dependent T cell developmental stages, such as negative selection, SP4 maturation, regulatory T cell and iNKT cell differentiation [1]. The co-expression of Helios and PD1 [21] on both DP thymocytes and CD4^+^ Foxp3^-^ SP thymocytes showed that cortical and medullary negative selection, respectively, remained unaltered in the 10-week-old Foxo3^cKO^ thymus (Fig. S5A). However, the mutant thymus showed a moderate increase in the proportion of immature CD24^hi^CD62L^lo^ CD4 SP thymocytes. Despite the partial block in SP4 maturation, the numbers of both immature and mature SP4 were diminished in adult Foxo3^cKO^ thymus, likely due to the aforementioned decline in total thymocyte cellularity (Fig. S5B). Strikingly, the percentage and number of mature (CD25^+^Foxp3^+^) regulatory T cells were significantly decreased in the adult Foxo3^cKO^ thymus (Fig. 5A). Although Foxo3-deficiency did not impact the percentages of immature regulatory T cells (CD25^+^Foxp3^-^ and CD25^-^Foxp3^+^ SP4s) [22], their numbers were also reduced in the mutant thymus (Fig. 5A). A complementary analysis excluding CD44^hi^ recirculating cells [22] confirmed that *Foxo3* deficiency significantly affected the frequency of newly generated regulatory T cells, resulting in a tendency towards a reduction in their numbers (Fig. S5C). We next set in vitro suppression assays using equal numbers of regulatory T cells from the control or mutant thymus, and assessed their cell-intrinsic capacity to suppress TCR-mediated polyclonal proliferation of WT conventional T cells (TCRβ^+^CD4^+^CD25^-^). The precursor frequency and average division of conventional T cells were higher when co-cultured with regulatory T cells isolated from the Foxo3^cKO^ thymus compared to the control (Fig. S5D), indicating that regulatory T cells derived from the Foxo3^cKO^ thymus showed lower suppressive function.

Besides their well-defined role in conventional αβ T cell development, mTECs also control the development of CD1d-restricted iNKT cells [23]. Using CD1d:PBS57 loaded tetramer, we found a significant reduction in the frequency and absolute number of iNKT cells in the adult Foxo3^cKO^ thymus (Fig. 5B). Following recognition of CD1d/glycolipid complexes, iNKT cells undergo a stepwise maturation program characterized by the loss of CD24 and acquisition of CD44 and NK1.1 [24]. We found a specific and significant decrease in the numbers of stage 1 (CD24^-^CD44^-^NK1.1^-^) and stage 2 (CD24^-^CD44^+^NK1.1^-^) iNKT subsets in the mutant thymus (Fig. S5E). Lastly, we analysed the expression of T-bet, PLZF and RORγt to determine whether *Foxo3* deficiency affected iNKT1, iNKT2 and iNKT17 cells [25]. Although the numbers of all three iNKT subsets decreased, the decline in iNKT2 cells in the Foxo3^cKO^ thymus was statistically more significant (Fig. 5C).

### Effect of TEC-specific deletion of *Foxo3* in peripheral immune tolerance

**Fig. 6:**
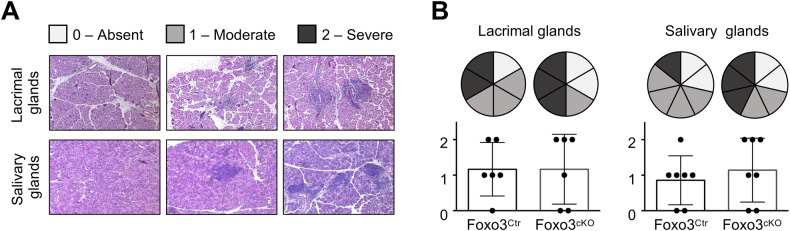
TEC-specific *Foxo3* deletion affects peripheral tolerance induction. **A** Representative H&E staining of the different degrees of infiltration. **B** Severity scores for inflammatory lymphocytic infiltration in salivary and lacrimal glands of 10 months-old Foxo3^Ctr^ and Foxo3^cKO^ mice. Pie charts represent absent, moderate and severe lesions as light grey, dark grey and black, respectively. Bar graphs represent absent, moderate and severe lesions scored as 0–2, respectively. Data are represented as mean ± SD.

Lastly, we investigated whether the aforementioned thymic phenotypes predisposed to changes in peripheral immune tolerance in aged (10-month-old) Foxo3^cKO^ mice. It is worth noting that Foxo3^cKO^ mice were generated in a C57Bl/6 background, which is known for its resistance to autoimmunity resulting from defects in central tolerance, such as impaired mTEC and/or Aire function [14, 26]. Although not statistically significant, the frequency of mice with severe lymphocytic infiltrations in the salivary glands was increased in aged Foxo3^cKO^ mice (Fig. 6A, B). Despite these mild alterations, we did not find changes in antinuclear antibodies in the serum of aged mutant mice (data not shown).

The mild signs of disturbed tolerance in aged Foxo3^cKO^ mice led us to determine the role of Foxo3 in the context of a genetically susceptible model for autoimmunity. To do so, we crossed Foxo3^cKO^ mice onto an Aire^KO^ background (Aire^KO^:Foxo3^cKO^) and determined whether *Foxo3* deficiency aggravated the autoimmune predisposition previously reported in *Aire*-deficient mice (Aire^KO^) [3]. Considering the description of the impact of *Foxo3* deficiency on the homeostasis and genetic program of TECs, T cell development, and tolerance in Figs. 1–5, as well as the challenges associated with establishing dual-deficient Aire^KO^:Foxo3^cKO^ mice, we did not reanalyse single Foxo3^cKO^ mice within this experimental complementary setup, which include Aire^WT^, Aire^KO^ and Aire^KO^:Foxo3^cKO^ groups. Thus, we did not directly compare the independent or interdependent role of Foxo3 and Aire.

Aire^+^ mTECs represent one of the most dominant mature mTEC lineages in the postnatal thymus [11, 12, 17, 27] and Aire deficiency is associated with defects in mTEC, regulatory T cell and iNKT cell differentiation [11, 16, 28–31]. We confirmed that Aire^KO^ (Aire^KO^:Foxo3^Ctr^) adult thymus had a reduced number of cTEC, mTEC^lo^ and an increase in mTEC^hi^ when compared to Aire^WT^ controls (Aire^WT^:Foxo3^Ctr^). Yet, the cellularity of cTEC, mTEC^lo^ and mTEC^hi^ subsets was further decreased in Aire^KO^:Foxo3^cKO^ age-matched thymus (Fig. S6A). Furthermore, we confirmed that the percentage and number of regulatory T cells and iNKT cells were reduced in the adult Aire^KO^ (Aire^KO^) thymus, and these defects were also further reduced in their Aire^KO^:Foxo3^cKO^ counterparts (Fig. S6B, C). These results suggested that *Foxo3* deficiency further accelerated the defects in TECs, regulatory T cell and iNKT cell development in Aire^KO^ mice. Considering that Aire^KO^ mice represent an early-onset autoimmunity model, we chose to anticipate the assessment of altered peripheral tolerance to 6 months of age. In line with previous studies in the C57BL/6 background [14, 26], aged Aire^KO^ mice showed moderate to severe lymphocytic infiltrates, mostly affecting the salivary and lacrimal glands, when compared to age-matched controls. Although lacking statistical significance, Aire^KO^Foxo3^cKO^mice showed a moderate aggravation compared to Aire^KO^ mice, with a higher number of animals displaying severe lymphocytic infiltrates in salivary glands (Fig. S6D). Analysis of other organs, including lacrimal glands, kidney and colon, showed no apparent differences between Aire^KO^ and Aire^KO^Foxo3^cKO^ mice (Fig. S6D), suggesting that this exacerbation effect could be organ-specific. Collectively, our findings suggest that *Foxo3* deficiency in TECs may predispose to failures in central tolerance, ultimately leading to impaired peripheral tolerance.

## Discussion

The differentiation of the TEC compartment is initiated during embryonic development and persists through adult life. This dynamic process is supported by the coordinated functioning of specialized bipotent precursors and restricted c/mTEC precursors [3]. Our findings imply that Foxo3 primarily controls the cellularity of mTECs. There are several possible explanations for our observations that are not mutually exclusive. Mature mTECs, particularly the Aire^+^ subset, are estimated to have a turnover rate of 7–10 days [32, 33], implicating the need for replacement by upstream progenitors. Thus, the reduction in Foxo3^cKO^ mTECs may stem from a failure in mTEC precursors and/or their capacity to replace mature lineages. As mTEC precursors exist within mTEC^lo^ subset [3], the more pronounced effect in the mTEC^lo^ subset would align with this possibility. Yet, mTEC^lo^ also include differentiated populations such as CCL21^+^ and Post-Aire cells. An alternative, and not mutually exclusive, possibility is that *Foxo3* deficiency may render mTECs more susceptible to death. The increase in annexin V^+^ in Foxo3^cKO^ mTEC^lo^ and the enrichment in genes involved in DNA-damage apoptosis found in Foxo3^cKO^ mTECs would support this hypothesis, suggesting that the mTEC^lo^ subsets are more sensitive to the absence of Foxo3. Further studies that aid in deciphering the signals responsible for activating Foxo3 in TECs and determining whether the function of Foxo3 is differentially regulated in distinct c/mTEC subsets will offer insights concerning the regulation of Foxo3-dependent pathways in TECs.

Although *Foxo3* deficiency predominantly affected mTEC cellularity, *Foxo3* was more expressed in cTECs than mTECs. Our transcriptomic data suggest that Foxo3 controls distinct genetic programs in each TEC subset. At present, the mechanisms governing cTEC homeostasis and biology remain unclear. Our results showed that *Foxo3* deficiency also influences the cTEC genetic program. While cTEC numbers were seemingly unaltered under steady-state conditions, the transcriptomic alterations in cTECs correlated with a decrease in the frequency of DN1 thymocytes and an overall reduction in cellularity of all thymocyte subsets in the adult Foxo3^cKO^ thymus. Yet, despite the reduction in thymic subsets, the percentages of cells in distinct subsequent stages of cortical T cell development (DN2-DN4, DP) were comparable between mutant and control mice. We can only speculate on how *Foxo3* deficiency affects the function of cTECs resulting in a reduced number of cortical DN1 and DP thymocytes in the adult thymus. cTECs provide key chemokines (Cxcl12, Ccl19, Ccl21), ligands (Dll4) and cytokines (Il7, Scf1) to regulate the attraction, commitment and proliferation of early T cell precursors, as well as self-peptides:MHC ligands for positive selection [3]. The absence of transcriptional alterations of these essential cTEC-derived factors may suggest that they are not the underlying cause for the reduction in the number of DP thymocytes in the adult Foxo3^cKO^ thymus. Concordantly, *Foxo3* deficiency appeared not to affect the rate at which DN intrathymic T cell precursors progress through T cell commitment and positive selection, as indicated by the similar frequency of cells within DN2-DN4, and pre and post-positive selection DP stages compared to controls. One possibility is that *Foxo3* deficiency affects the entry of early thymic precursor and/or the DN1 niche may be affected in the mutant thymus, which would lead to a reduction in the number of DN1 cells and their downstream progeny, DN2-DN4 and DPs. Our data suggest that Foxo3 restrains ribosomal biogenesis in cTECs. Previous studies in *D. melanogaster* and *C. elegans* have demonstrated that Foxo3 suppresses protein translation in response to reduced insulin signalling or stress signals, such as nutrient deprivation or reactive oxygen species [34, 35]. In this scenario, *Foxo3* deficiency in cTECs may lead to enhanced translation, resulting in an enrichment of functional processes that control protein synthesis, including ribosome biogenesis. It remains to be determined whether these molecular changes in cTECs, or the ones reported in mTECs, contribute to anatomic alterations in the cortical niche and/or perturb the corticomedullary junction, thereby affecting the entry, migration or nurture of DN1 cells. This scenario could also involve a possible complex functional and structural interplay between cTECs, mTECs and other non-epithelial thymic stromal cells (eg, endothelia and mesenchyme). Seminal research has shown that distinct clonally derived medullary islets formed during the embryonic period expand during postnatal life and merge into larger medullary areas in the adult thymus [36]. The reduced mTEC compartment in the adult Foxo3^cKO^ thymus was associated with fewer medullary islets, which may affect the spatial organization of mTECs and their relationship with other stromal components. Future studies 3D reconstruction studies of the Foxo3^cKO^ thymus should investigate the effects of Foxo3 on the organization and position of diverse cTEC and mTEC subsets, and determine their potential impact on T cell development.

The role of Foxo3 in regulating the integrity of the mTEC compartment is in line with its role in preserving genomic stability and control of cell cycle [7, 15]. Moreover, we infer that the higher frequency of cycling cells in *Foxo3*-deficient mTEC^lo^ could be due to a compensatory response triggered by apoptosis following DNA damage and/or related to the absence of Foxo3-dependent regulation of cell cycle arrest [7]. The most significant difference in the frequency of cells with DNA damage (*γ*H2AX^+^) was found in the Foxo3^cKO^ mTEC^hi^ subset. Intriguingly, previous studies have connected double-strand DNA breaks to Aire-mediated TRA expression [37]. While most of the studies have primarily focused on mTEC^hi^, it is worth noting that TRA expression occurs in both Aire^-^ mTEC^hi^ and mTEC^lo^ subsets, partly regulated by Fezf2 and other unidentified transcriptional regulators [8, 38]. It is conceivable that the unique epigenetic and transcriptional states of mTECs, particularly related to TRA expression, initiate DNA breaks that require a reparative DNA damage response. Our transcriptional analysis revealed that Foxo3^cKO^ mTECs predominantly altered the expression of Aire-independent TRAs. Given that mTECs express clusters of TRAs at a single-cell level [38–40], the susceptibility of Foxo3-deficient cells to apoptosis may lead to alterations in the representation of particular mTEC clusters, and TRAs, in mutant mTECs. In this regard, the decline in the mTEC compartment and altered transcriptome of mTECs caused by *Foxo3* deficiency mirrors some of the changes found in the context of *p53* deficiency [5]. p53 directly targets and activates the expression of *Foxo3* [41], and Foxo3 can promote p53 stabilization and p53-driven apoptosis [42]. Conversely, Foxo3 can also be negatively regulated by p53 through the activity of serum and glucocorticoid-inducible kinase 1 (SGK1) [43]. Additionally, under oxidative stress, p53 can inhibit Foxo3 transcriptional activity [44]. Thus, the functional relationship between p53 and Foxo3 appears to be context-dependent. Albeit *Foxo3* is downregulated in p53^cKO^ mTECs, *Trp53* remains unaltered in Foxo3^cKO^ mTECs, suggesting that Foxo3 may operate downstream of p53 in controlling mTEC homeostasis. In this regard, approximately 46% of DEGs found in Foxo3^cKO^ mTECs were also affected by *Trp53* deficiency, indicating that Foxo3 and p53 control the expression of common targets in mTECs. Still, the significant number of DEGs affected only by *Foxo3* deficiency infers the existence of other p53-independent mechanisms to control Foxo3 in mTECs. We hypothesize that Foxo3 and p53 collaboratively control common and specific regulatory pathways within mTECs. Future studies should investigate how Foxo3 controls DNA repair and cell cycle in mTECs, and how these processes connect to promiscuous gene expression and the maintenance of their function.

The perturbation in the mTEC compartment of Foxo3^cKO^ mice predominantly impacts specific stages of T cell development, namely iNKT cells and regulatory T cells. These observations are in line with previous studies implicating mTECs in the development of these specific T cell subsets [23, 45]. CCL21^+^ and thymic tuft mTECs have been shown to control the development of functional iNKT sublineages [46]. Specifically, the decline in iNKT2 cells in the Foxo3^cKO^ thymus aligns with the reduction in tuft cell-like mTECs, known to regulate iNKT2 cell development [12, 46]. Moreover, our findings indicate that the Foxo3^cKO^ mTEC microenvironment compromises the development and function of regulatory T cells, potentially predisposing to failures in peripheral immune tolerance. There are several possibilities for the reduced development and suppressive function of regulatory T cells in a polyclonal non-antigen-specific setting, which may not be mutually exclusive. These could involve a reduction in the size of the mTEC compartment, alterations in processing or presentation of self-antigen:MHC II complexes by Foxo3^cKO^ mTECs and/or failure in other mTEC-derived signals critical for T regulatory cell differentiation [3]. Future studies should clarify if these defects in regulatory T cells are general or antigen-specific. The observations that *Foxo3* deficiency further potentiated the previously reported defects in mTEC, iNKT and regulatory T cell development in Aire^KO^ mice were intriguing. Further studies are required to define the interdependent or independent roles of Foxo3 and Aire in mTECs and how these affect iNKT and regulatory T cell development and function. Still, the mild autoimmune manifestations detected in Foxo3^cKO^ mice, either in Aire^WT^ or Aire^KO^ settings, may be attributed to the resistant C57BL/6 background and/or the presence of extrathymic T regulatory cell-dependent compensatory mechanisms [26, 47], which cooperatively might be sufficient to prevent the development of overt autoimmunity. Previous studies have shown that Aire-dependent regulatory T cells, which are established during the perinatal stages of life, play a crucial role in protecting against autoimmune responses later in life [47, 48]. Notably, the differentiation of mTECs and regulatory T cells in Foxo3^cKO^ thymus seemed to be normal during the perinatal period, with the more severe thymic phenotype unfolding in adulthood. We reason that the Foxo3^cKO^ thymus may establish a functional pool of regulatory T cells during early life, which serves to prevent an overt autoimmune response in aged mice. Additionally, in C57BL/6 mice, PD1-expressing conventional T cells and regulatory T cells accumulate in nonlymphoid organs of Aire^KO^ mice [26, 47], presumably contributing to an immunosuppressive state for otherwise more severe autoimmunity observed in different genetic backgrounds.

Our findings on Foxo3 align with previous studies that establish a link between mTEC homeostasis, TRA expression, the initiation of a DNA damage response and induction of immunological tolerance [4, 5]. Worth noting, Foxo3 has additional roles in response to other physiologic stress stimuli, including growth factor deprivation, metabolic stress and oxidative stress [6, 49–52]. In this regard, other Foxo family proteins, including Foxo1, Foxo4 and Foxo6, control basic cellular processes in response to nutrient deprivation and stress [6, 7]. Future studies must clarify the individual and cooperative functions of other Foxo proteins in the control of TEC homeostasis, akin to the collaborative roles attributed to the Foxo family in other tissues [53–55]. In conclusion, our study underscores the important influence of Foxo3 in controlling TEC biology and function.

## Methods and Materials

### Mice

All mice were in a C57BL/6 background and housed under specific pathogen-free conditions. Foxo3^fl/fl^ and Aire^−/−^ were purchased from The Jackson Laboratory [9, 56]. Foxn1^Cre^ mice were obtained from Dr. Thomas Boehm [57]. The required number of animals in each experimental setting was estimated based on results obtained with similar procedures to those in previous studies [5], considering both biological and technical variability. Apart from categorization based on genotype, mice were randomly assigned to particular treatment and timepoint experimental groups, irrespectively of sex, and no exclusions were made. Whenever feasible, sample preparation and data acquisition were conducted in a blinded manner with respect to the genotype. In radiation recovery studies, mice received sublethal total-body irradiation (450 rads) with a Cs^137^ radiation source (Gammacell 1000, Nordion). Experiments were performed in accordance with European guidelines for animals used for scientific purposes (Directive 2010/63/EU).

### PCR genotyping

*Foxo3* inactivation was confirmed by PCR analysis (using primers *a*, *b* and *d*) to detect the excision of exon 2, the first coding exon, which encodes the start codon and the N-terminal half of the protein and forkhead DNA binding domain [9]. Primers spanning the *loxP* site in intron 1, *a* (F 5′-AACAACCTCACACATGTGCC-3′) and *b* (R 5′- AGTGTCTGATACCGAAGAGC-3′) were used to detect the Foxo3^fl^ allele. The excision was detected using primers *a* and *d* (R 5′-CATGCAGTCCGAGAGATTTG-3).

### Flow Cytometry and cell sorting

For hematopoietic cell analysis, thymocytes were obtained from mechanically disrupted thymus samples and subsequently stained with antibodies listed below for T cell development characterization [5, 58, 59]. This procedure liberates a significant fraction of hematopoietic cells while preserving most of thymic stroma cells. For stromal cell analysis, the remaining thymic fragments underwent enzymatic digestion, resulting in a cell suspension that was subjected to CD45^+^ cell depletion using anti-CD45 microbeads and LS columns (Miltenyi Biotec), as previously described [5, 58–60]. The total thymocyte cellularity was estimated by adding the number of hematopoietic cells (CD45^+^) obtained from the mechanically liberated fraction and the number of hematopoietic cells (CD45^+^) obtained from the digested fraction before anti-CD45 depletion. The number of distinct T cell subsets was estimated from the total thymocyte cellularity. The total TEC cellularity was estimated by determining the number of TECs (CD45^-^EpCAM^+^) obtained from the digested fraction after CD45 depletion, as previously described [5, 58, 59]. Enriched TEC samples were analysed using the following antibodies: anti-CD45.2 (104, eBioscience, cat. no.: 45-0454-82), anti-EpCAM (G8.8, Biolegend, cat. no.: 118225) or anti-EpCAM (G8.8, Biolegend, cat. no.: 118212), anti-Ly51 (6C3, eBioscience, cat. no.: 12-5891-82), anti-I-A/I-E (M5/114-15-2, eBioscience, cat. no.: 47-5321-82), anti-CD80 (16-10A1, Biolegend, cat. no.: 104731). Biotinylated UEA-1 (Vector Laboratories) was detected with streptavidin Brilliant Violet 711 conjugate (Biolegend, cat. no.: 405241) or streptavidin PE-Cy7 conjugate (eBioscience, cat. no.: 25-4317-82). For intracellular stainings, we used a Foxp3 staining buffer set (eBioscience). Intracellular markers were analysed using the following antibodies: anti-CCL21 (59106, R&D Systems, IC457G-100UG), anti-Aire (5H12, eBioscience, cat. no.: 50-5934-82), anti-γH2AX (2F3, Biolegend, cat. no.: 613405) and anti-Ki67 (11F6, Biolegend, cat. no.: 151204), as described [5, 58, 59]. Intracellular detection of DCLK1 was achieved using anti-DCLK1 (DCAMKL1, Abcam, cat. no.: Ab31704), followed by donkey anti-rabbit Alexa Fluor 488 (Invitrogen, cat. no.: A21206). Apoptosis was assessed using FITC Annexin V (Biolegend, cat. no.: 640905), 7-AAD (Biolegend, cat. no.: 420403) and Annexin V Binding Buffer (Biolegend, cat. no.: 422201).

For hematopoietic cell analysis, thymocyte samples obtained from mechanically disrupted thymus were stained for flow cytometry analysis. The complete list of antibodies can be found in Table 1. CD1d tetramers loaded with PBS57 or unloaded CD1d tetramers were obtained from the National Institutes of Health Tetramer Core Facility. All intracellular staining was performed using a Foxp3 staining buffer set (eBioscience). Flow cytometry analyses were performed on an LSR Fortessa (Becton Dickinson) and cell sorting was performed on a FACS Aria II (Becton Dickinson). Data were analysed on FlowJo software.

**Table 1:** Antibodies used in flow cytometry analysis of hematopoietic populations in thymus samples.

Specificity	Clone	Supplier	Cat. No.
CD177	2B8	eBioscience	47-1171-82
CD11b	M1/70	eBioscience	50-0112-82
CD11c	N418	eBioscience	50-0114-82
CD19	eBio1D3	eBioscience	50-0193-82
CD24	M1/69	eBioscience	48-0242-82
CD24	M1/69	Biolegend	101831
CD25	PC.61.5	eBioscience	25-0251-82
CD4	GK1.5	Biolegend	100453
CD44	IM7	Biolegend	103022
CD44	IM7	Biolegend	103049
CD5	53-7.3	Biolegend	100605
CD62L	MEL-14	Biolegend	104437
CD69	H1.2F3	Biolegend	104511
CD8	53-6.7	Biolegend	100741
FoxP3	FJK-16s	eBioscience	17-5773-80
GR1	RB6-8CS	eBioscience	50-5931-82
Helios	22F6	Biolegend	137204
NK 1.1	PK136	Biolegend	108713
NK1.1	PK136	eBioscience	17-5941-82
PD1	29F-1A12	Biolegend	135229
PLZF	Mags.21F7	eBioscience	53-9320-80
RORgt	Q31-378	BD	564723
T-bet	4B10	Biolegend	644832
TCRβ	H57-597	eBioscience	12-5961-82
TCRβ	H57-597	eBioscience	45-5961-80
TER-119	TER-119	eBioscience	17-5921-82
γδ TCR	eBioGL3	eBioscience	17-5711-82

### RNA sequencing

The total RNA library preparation and high-throughput sequencing of sorted cTEC/mTEC samples from 6 weeks-old Foxo3^cKO^ and Foxo3^Ctr^ littermates were performed at Gene Core facility (EMBL, Germany) as previously described [5, 58]. Fourteen sequencing libraries (three for cTEC control, three for cTEC Foxo3 cKO, four for mTEC control, four for mTEC Foxo3 cKO) were prepared using NEB Next RNA ultraprotocol (#E7530 NEB). Obtained libraries were quantified fluorimetrically, pooled in equimolar amounts and sequenced on the Illumina NextSeq sequencer (NextSeq 2000 P3 flowcell) in single-end mode (75 bases), following manufacturer’s instructions (Illumina). The reads were aligned to the mouse genome (mm10) using STAR (version 2.4.2a) with mm10 GTF annotation. The number of reads per gene was generated during alignment step (quantMode GeneCounts) and gene counts were then analyzed with DESeq2 package [61], as previously done [5, 58, 62]. An average of 58 million reads were mapped per Foxo3^Ctr^ cTEC and Foxo3^cKO^ cTEC samples, and 49 and 51 million reads were mapped per Foxo3^Ctr^ mTEC and Foxo3^cKO^ mTEC samples, respectively. Reads per kb of genes per million mapped reads (RPKM) values were calculated based on the normalized read counts. We selected differentially expressed genes based on the adjusted p-value lower than 0.1. Reads per kb of exon model per million mapped reads (RPKM) values were computed from normalized read counts. Gene ontology (GO) enrichment analysis was performed using model-based gene set analysis (MGSA) [63]. The analysis was performed with 20 independent runs of the Markov chain of 1.109 steps each. For each parameter, we used a regularly spaced grid with 11 points. The search intervals for the parameters p, alpha, and beta were set to [0.001, 0.1], [0, 0.2], and [0.5, 0.9] respectively for the search on biological process terms, and [0.001, 0.1], [0, 0.15], and [0.5, 0.9] for the search on molecular function terms. Functional categories with a marginal posterior probability estimate higher than 0.65 were retained for further analysis. The hierarchical clustering, represented as a dendrogram, of TEC populations were performed using the hclust function in R on euclidean distances between the variance of the log-transformed read counts for each genes across samples. The sequencing reads have been submitted to ENA (http://www.ebi.ac.uk/ena) and further information and resources, code and methods will be accessible under the following project number: PRJEB64863 (available upon publication). Requests for further information should be directed to and will be fulfilled by the corresponding author.

### Comparative transcriptomic analysis

Analysis of publicly available RNA-seq data and identification of the specific genes of different mTEC subpopulations was performed as previously described [58]. Briefly, the specific gene signatures of mTEC I, mTEC II, mTEC III, and mTEC IV [12] were obtained by extracting the top 200 most expressed genes for which the relative expression in the selected population was ≥ 2-fold the expression in all other populations. The specific gene signatures of early-, pre-, late-, and post-Aire mTEC [11] were obtained by extracting the top 200 most expressed genes with z-score ≥ 1.5. Mimetic and transit-amplifying subset signatures were derived from differentially expressed genes by filtering for genes unique to each cluster with fold-change > 2 and adjusted p < 0.01 [13]. The proliferating TEC signature was used as provided [16, 17].

### Regulatory T cell suppression assay

For in vitro suppression assays, 5 × 10^4^ control conventional T cells (CD4^+^CD25^-^) were labelled with1 μM CFSE after cell sorting and cultured in duplicates for 3 days in round bottom 96-well plates in the presence of 1 µg/ml anti-CD3 (145-2C11; BD Pharmingen), γ-irradiated splenocytes (200 rads; 1 × 10^5^ /well), and different ratios of either control or Foxo3 cKO-derived regulatory T cells (CD4^+^CD25^+^). Suppressive ability of regulatory T cells was assessed based on conventional T cell division, measured by the number of cells found within discrete levels of CFSE dilution. The average number of divisions was calculated for cells that performed at least one division. The precursor frequency of dividing cells (percentage of cells in the initial population that had undergone division) was calculated as follows: [∑_n≥1_(P_n_/2^n^)]/[∑_n≥0_(P_n_/2^n^)], where *n* is the division number that cells have gone through and P_*n*_ is the number of cells in division *n* [64].

### Histology

Paraffin-embedded tissue sections of thymus, lacrimal gland, salivary glands, stomach, colon, pancreas, kidney and liver were stained with haematoxylin and eosin (H&E). Analysis was done on a light microscope (Olympus CX31) and images were captured using a brightfield microscope (Leica DM2000 LED). Thymic section images were processed using Fiji software. Histopathology was scored in a blind and randomized fashion by four independent observers. For organs in which no lymphocytic infiltration is detected in any animal of the genotypes under comparison, data are not shown.

### Statistical analysis

Statistical analyses were performed using GraphPad Prism software. The 2-tailed Mann-Whitney test was used for all statistical analyses. Sample sizes (n) and number of experiment replications are reported in the respective figure legends. Samples correspond to biological replicates. Significant *P*-values are as follows: *P < 0.05; **P < 0.01; ***P < 0.001. Nonsignificant differences are not specified.

### Supplementary information

Supplementary Figures

Supplementary Figure Legends

Supplementary Tables

## Data Availability

Data was deposited in the GEO database (PRJEB64863). Requests for further information should be directed to and will be fulfilled by the corresponding author.
