# Supplementary material for: Foxo3 regulates cortical and medullary thymic epithelial cell homeostasis with implications in T cell development
Source: Cell Death Dis. 2024 May 21;15(5):352. doi: 10.1038/s41419-024-06728-0 (PMC11109193; doi:10.1038/s41419-024-06728-0)
Supplement: Supplementary file 1 — Supplementary Figures [file 41419_2024_6728_MOESM1_ESM.pdf]

Supplementary Figure 1

A

Gene	Rodrigues *et al.* 2018		Sansom *et al.* 2014	
	FPKM cTEC	FPKM mTEC	FPKM cTEC	FPKM mTEC
Foxo3	26.6	4.3	37.6	6.3
Batf	0.3	7.2	0.2	5.0
Atrx	4.9	5.8	20.0	19.8
Foxm1	4.3	3.9	11.3	6.2
E2f7	0.7	1.9	2.4	3.4
Tfap4	5.5	5.9	4.4	2.7
Trp53	15.3	19.9	69.5	69.8

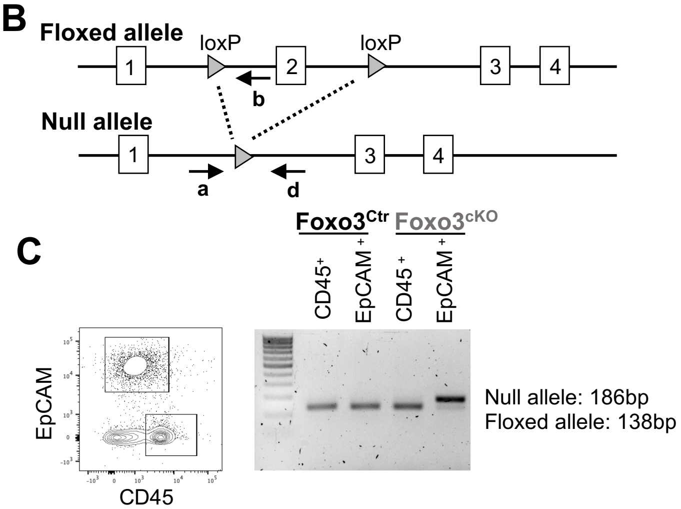

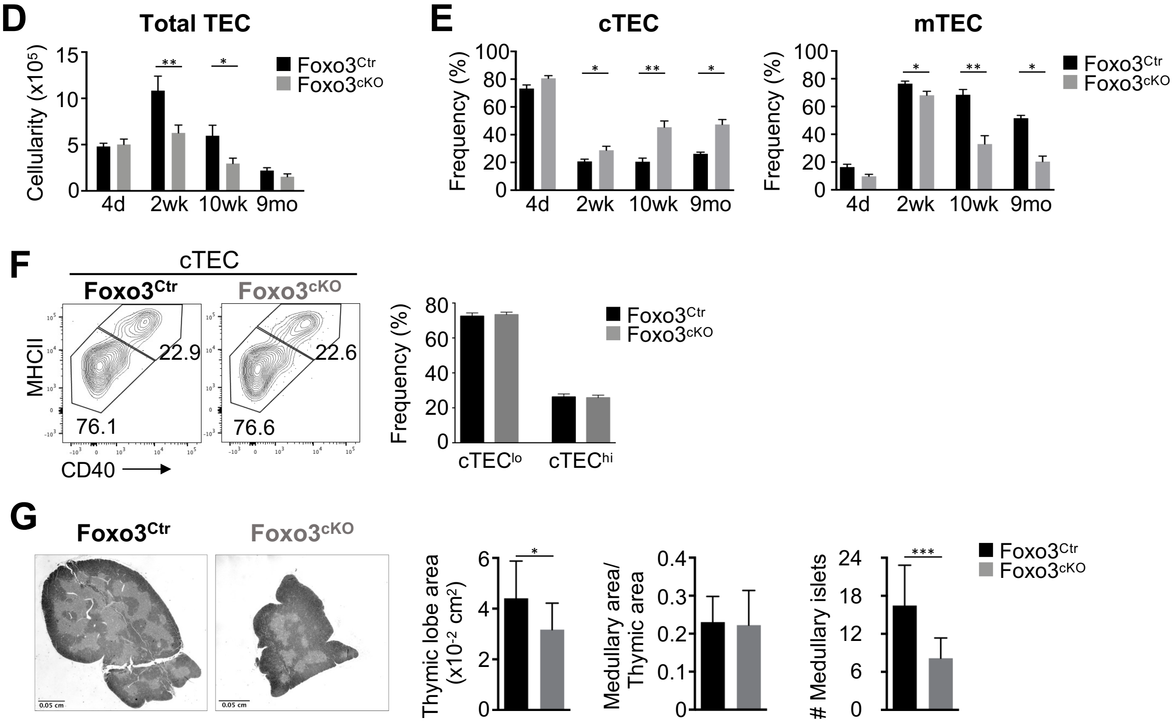

Supplementary Figure 2

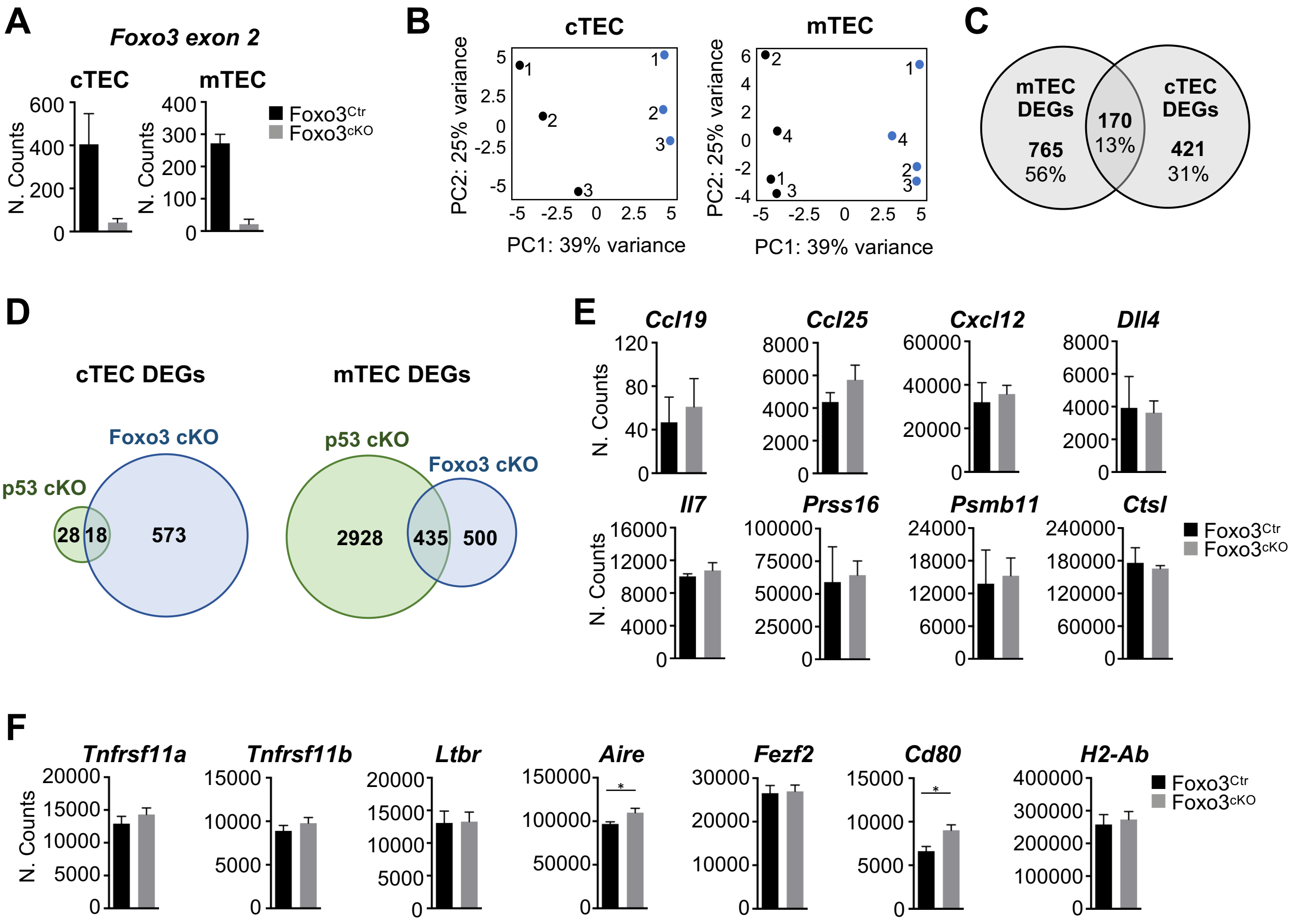

Supplementary Figure 3

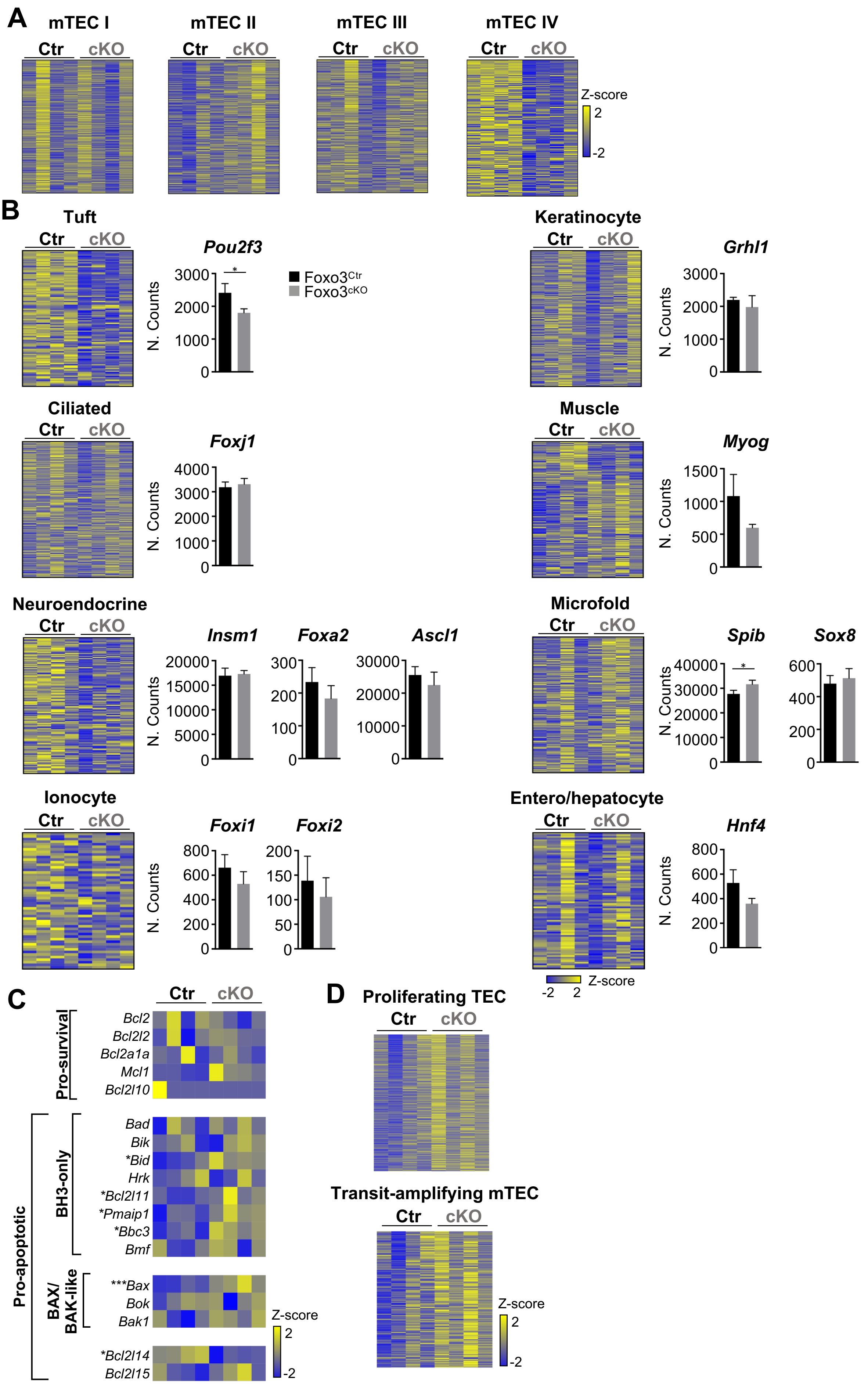

Supplementary Figure 4

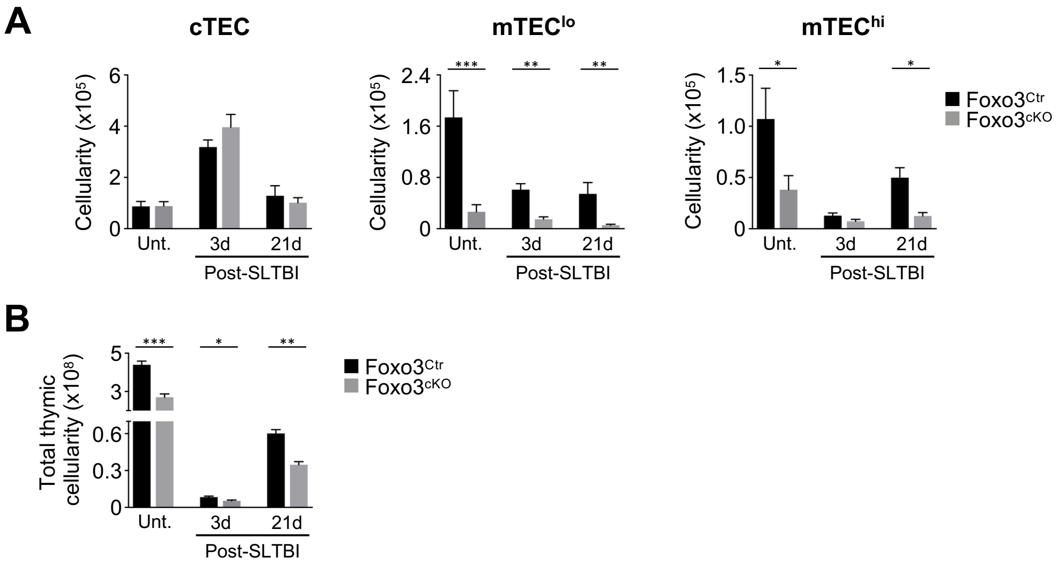

Supplementary Figure 5

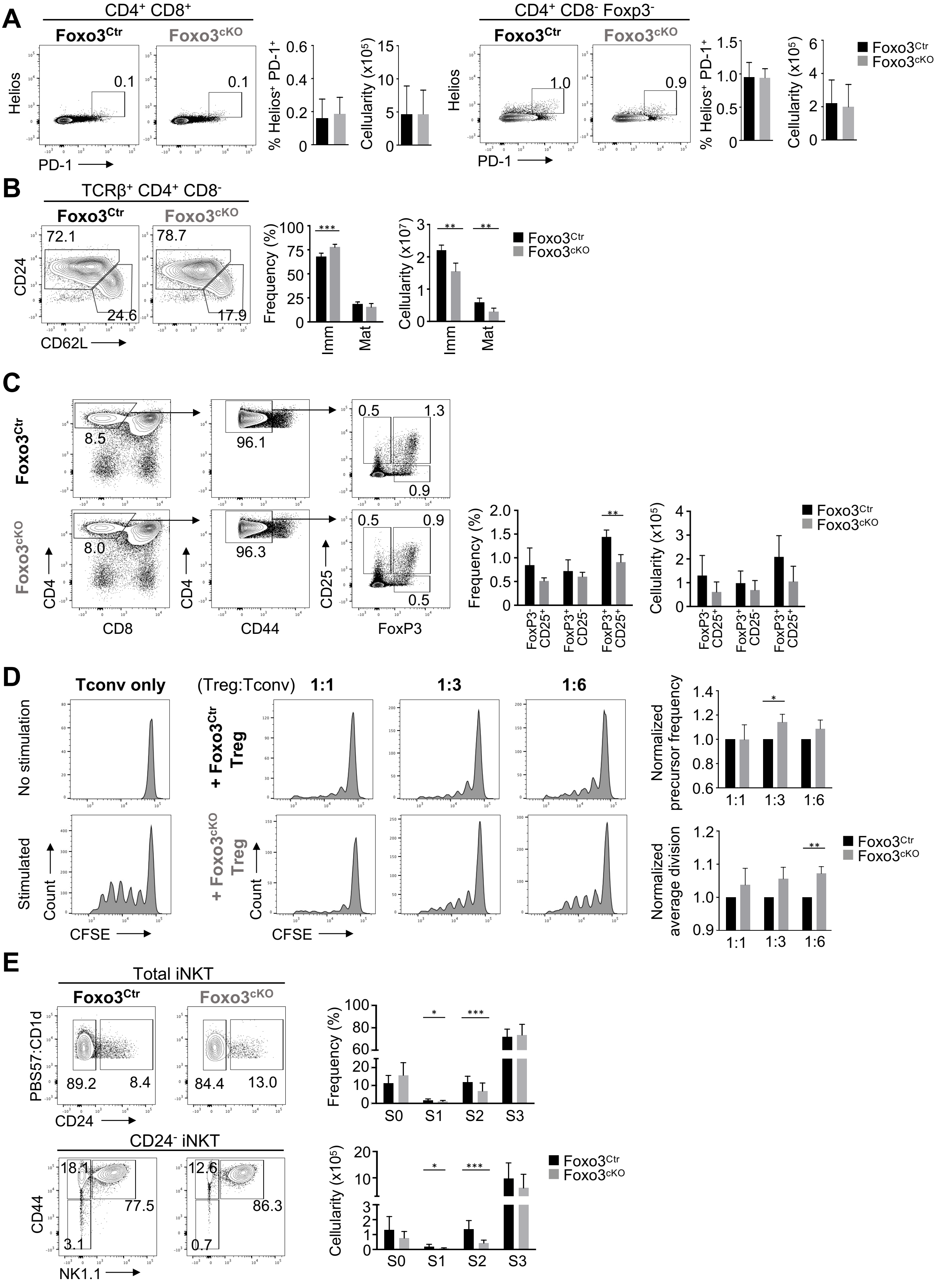

Supplementary Figure 6

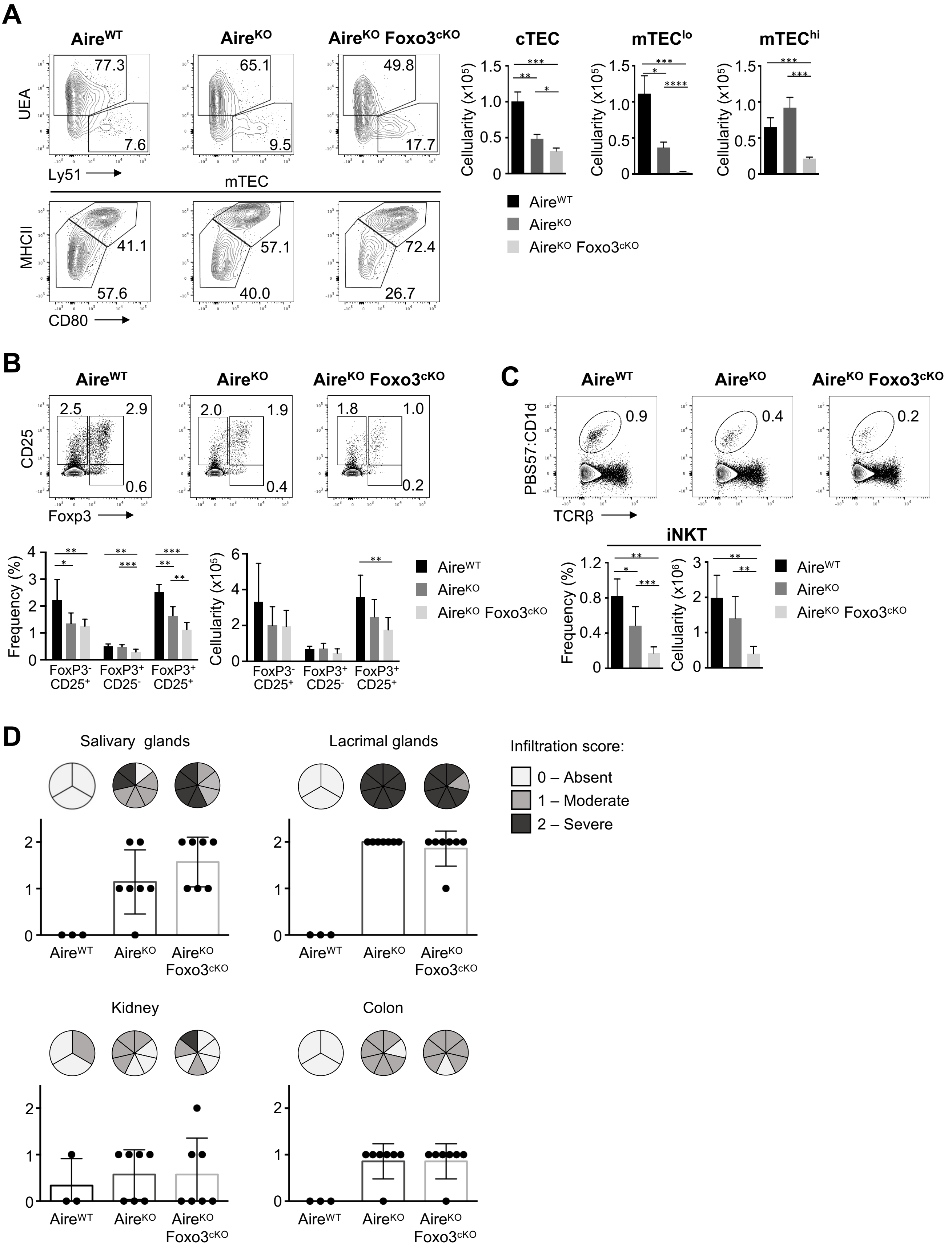

Supplementary figure legends:

Supplementary figure 1. Targeting *Foxo3* expression in TECs - (A) The gene list shows the transcription factor-coding genes found in *Mus musculus* annotated under the gene ontology term GO:0030330 "DNA damage response, signal transduction by p53 class mediator". FPKM was obtained from RNA sequencing analysis of cTECs and mTECs from postnatal thymus. **(B)** Genomic structure of *Foxo3*^{floxed} and *Foxo3*^{cKO} null alleles. Primers used for PCR (a + b + d) are indicated by small arrows. Grey triangles represent the loxP sequences. Represented exon and intron sizes are not to scale. **(C)** Confirmation of Foxn1^{Cre}-driven *Foxo3* exon 2 deletion by genomic PCR analysis of FACS-sorted thymocytes (CD45^{+}) and TECs (EpCAM^{+}) from Foxo3^{Ctr} (Foxo3$^{fl/fl}$) and Foxo3^{cKO} (Foxn1^{Cre}:Foxo3$^{fl/fl}$) mice. **(D)** Total TEC numbers in thymi isolated at the indicated time points. **(E)** Frequency of cTECs and mTECs at the indicated time points. **(F)** cTEC^{lo} (MHCII^{low}CD40^{low}) and cTEC^{hi} (MHCII^{high}CD40^{high}) composition within total cTECs of the 10-week-old adult thymus. Data are representative of 2 or 3 independent experiments per time-point (n=6-9 independent samples). All data are represented as mean ± SEM. **(G)** Thymic sections from 10-week-old Foxo3^{Ctr} and Foxo3^{cKO} mice stained with hematoxylin and eosin (H&E). Bar graphs depict mean and SD of thymic lobe area, medulla/total area ratio and medullary islet number measured on 3 sections per thymus from 5 Foxo3^{Ctr} and 5 Foxo3^{cKO} mice. wk - weeks; mo - months. *$P < 0.05$; **$P < 0.01$; ***$P < 0.001$.

Supplementary figure 2. Transcriptome analysis of Foxo3^{cKO} cTECs and mTECs - (A) Expression of exon 2 of *Foxo3* gene obtained from RNA-Seq analysis of FACS-sorted cTECs and mTECs purified from 6-week-old Foxo3^{Ctr} and Foxo3^{cKO} mice. **(B)** Principal component analysis (PCA) obtained from RNA-Seq analysis of cTECs and mTECs isolated from Foxo3^{Ctr} and Foxo3^{cKO} thymus. **(C)** Venn diagram depicts the number of differentially expressed genes detected in cTECs, mTECs and commonly detected in both cTECs and mTECs. **(D)** Venn diagrams depict the specific and common DEGs resulting from *Trp53* or *Foxo3* deficiency in cTECs (left) and mTECs (right). **(E)** Expression of cTEC-specific genes in Foxo3^{Ctr} and Foxo3^{cKO} cTECs. **(F)** Expression of mTEC-specific genes in Foxo3^{Ctr} and Foxo3^{cKO} mTECs. All data are represented as mean ± SD. *$P < 0.05$; **$P < 0.01$; ***$P < 0.001$.

Supplementary figure 3. Analysis of genetic signature of mTEC subsets in Foxo3^{cKO} mTECs - (A) Relative expression level of genes specifically upregulated in mTEC I, mTEC II, mTEC III and mTEC IV (data from (12)) in Foxo3^{Ctr} and Foxo3^{cKO} mTECs. **(B)** Relative expression level of genes specifically upregulated in mimetic mTEC subsets (as defined by Michelson et al., 2022) in Foxo3^{Ctr} and Foxo3^{cKO} mTECs. The expression of subset-specific transcription factor genes was measured in Foxo3^{Ctr} and Foxo3^{cKO} mTECs and is depicted on the right of the corresponding heatmaps. Bar graphs

show mean ± SD. *$P < 0.05$; **$P < 0.01$; ***$P < 0.001$. **(C)** Foxo3^{Ctr} and Foxo3^{cKO} mTEC samples were analysed for the relative expression of genes coding for pro-survival and pro-apoptotic Bcl2 family members. Statistically significant differentially expressed genes are marked as * (P< 0.05), ** (P< 0.01) or *** (P< 0.001). **(D)** Relative expression level of genes specifically upregulated in proliferating and transit-amplifying mTECs (as defined by Baran-Gale et al., 2020, and Michelson et al., 2022) in Foxo3^{Ctr} and Foxo3^{cKO} mTECs.

Supplementary figure 4. Analysis of TEC cellularity recovery following SL-TBI - 10-week-old Foxo3^{Ctr} and Foxo3^{cKO} mice were subjected to sublethal total-body irradiation (SLTBI) and analysed at day 3 and day 21 post-irradiation. 10-week-old Foxo3^{Ctr} and Foxo3^{cKO} untreated mice (Unt.) were also analysed. The cellularity of cTECs, mTEC^{lo} and mTEC^{hi} **(A)** and total thymic cellularity **(B)** were determined at the indicated time-points. Data are representative of 2 or 3 independent experiments (n=6-9 independent samples) and represented as mean ± SEM. *$P < 0.05$; **$P < 0.01$; ***$P < 0.001$.

Supplementary figure 5. T cell development in the Foxo3^{cKO} thymus - (A) Expression of Helios and PD-1 on CD4$^+$ CD8$^+$ DP thymocytes (left) and on CD4$^+$ CD8$^-$ FoxP3$^-$ thymocytes (right). **(B)** Expression of CD24 and CD62L on TCRβ$^+$ CD4$^+$ CD8$^-$ thymocytes. Bar graphs show absolute cell numbers and percentages. Data are representative of 3 independent experiments (n=9 independent samples). **(C)** CD25 and Foxp3 expression on non-recirculatory CD44$^{-/lo}$ SP4 thymocytes in the 10-week-old thymus of Foxo3^{Ctr} and Foxo3^{cKO} mice. All data are represented as mean ± SD. **(D)** *In vitro* suppression assay measuring CFSE labelling in conventional T cells (Tconv) from Foxo3^{Ctr} thymus (WT) on day 3 of stimulation and co-culture with Foxo3^{Ctr} or Foxo3^{cKO}-derived thymic regulatory T cells (Treg) at the indicated Treg:Tconv ratios. Graphs represent the average number of divisions and precursor frequency normalized relatively to the corresponding conditions with Foxo3^{Ctr} Tregs, which were set to 1. Results are presented as mean ± SEM of 4 independent experiments. **(E)** Expression of CD24, CD44 and NK1.1 on iNKTs for the quantification of stage 0 (CD24$^+$), stage 1 (CD24$^-$CD44$^-$NK1.1$^-$), stage 2 (CD24$^-$CD44$^+$NK1.1$^-$) and stage 3 (CD24$^-$CD44$^+$NK1.1$^+$). Bar graphs show absolute cell numbers and percentages. Data are representative of 2 to 4 independent experiments (n=6 to 12 independent samples). Data are represented as mean ± SD. *$P < 0.05$; **$P < 0.01$; ***$P < 0.001$.

Supplementary figure 6. Effects of TEC-specific *Foxo3* deletion in Aire-independent mTEC lineages and in the autoimmune syndrome of Aire^{KO} mice- (A) TECs from 10-week-old Aire^{WT}, Aire^{KO} and Aire^{KO}Foxo3^{cKO} thymus were analysed for cTEC and mTEC composition (top). Total mTECs were analysed for mTEC^{lo} and mTEC^{hi} composition (bottom). Bar graphs show absolute cell numbers. Data are representative of 4 independent experiments (n=5-10 independent samples). Data are

represented as mean ± SEM. **(B)** TCRβ$^+$ CD4$^+$ thymocytes from Aire^{WT}, Aire^{KO} and Aire^{KO}Foxo3^{cKO} thymus were analysed for CD25 and Foxp3 expression **(C)** Total thymocytes from Aire^{WT}, Aire^{KO} and Aire^{KO}Foxo3^{cKO} thymus were analysed for expression of TCRβ and reactivity with PBS57-loaded CD1d tetramer. Bar graphs show absolute cell numbers and percentages. Data are representative of 4 independent experiments (n=5-10 biologically independent samples). Data are represented as mean ± SD. **(D)** Severity scores for inflammatory lymphocytic infiltration in salivary glands, lacrimal glands, kidney and colon of 6 months-old Aire^{WT}, Aire^{KO} and Aire^{KO}Foxo3^{cKO} mice. Pie charts represent absent, moderate and severe lesions as light grey, dark grey and black, respectively. Bar graphs represent absent, moderate and severe lesions scored as 0-2, respectively. Data are represented as mean ± SD. *$P < 0.05$; **$P < 0.01$; ***$P < 0.001$.
