# Supplementary material for: Foxo3 regulates cortical and medullary thymic epithelial cell homeostasis with implications in T cell development
Source: Cell Death Dis. 2024 May 21;15(5):352. doi: 10.1038/s41419-024-06728-0 (PMC11109193; doi:10.1038/s41419-024-06728-0)
Supplement: Supplementary file 2 — Supplementary Figure Legends [file 41419_2024_6728_MOESM2_ESM.docx]

**Supplementary figure legends:**

**Supplementary figure 1. Targeting *Foxo3* expression in TECs - (A)** The gene list shows the transcription factor-coding genes found in *Mus musculus* annotated under the gene ontology term GO:0030330 “DNA damage response, signal transduction by p53 class mediator”. FPKM was obtained from RNA sequencing analysis of cTECs and mTECs from postnatal thymus. **(B)** Genomic structure of *Foxo3*^floxed^ and *Foxo3*^cKO^ null alleles. Primers used for PCR (a + b + d) are indicated by small arrows. Grey triangles represent the loxP sequences. Represented exon and intron sizes are not to scale. **(C)** Confirmation of Foxn1^Cre^-driven *Foxo3* exon 2 deletion by genomic PCR analysis of FACS-sorted thymocytes (CD45^+^) and TECs (EpCAM^+^) from Foxo3^Ctr^ (Foxo3^fl/fl^) and Foxo3^cKO^ (Foxn1^Cre^:Foxo3^fl/fl^) mice. **(D)** Total TEC numbers in thymi isolated at the indicated time points. **(E)** Frequency of cTECs and mTECs at the indicated time points. **(F)** cTEC^lo^ (MHCII^low^CD40^low^) and cTEC^hi^ (MHCII^high^CD40^high^) composition within total cTECs of the 10-week-old adult thymus. Data are representative of 2 or 3 independent experiments per time-point (n=6-9 independent samples). All data are represented as mean ± SEM. **(G)** Thymic sections from 10-week-old Foxo3^Ctr^ and Foxo3^cKO^ mice stained with hematoxylin and eosin (H&E). Bar graphs depict mean and SD of thymic lobe area, medulla/total area ratio and medullary islet number measured on 3 sections per thymus from 5 Foxo3^Ctr^ and 5 Foxo3^cKO^ mice. wk - weeks; mo - months. **P* < 0.05; ***P* < 0.01; ****P* < 0.001.

**Supplementary figure 2. Transcriptome analysis of Foxo3^cKO^ cTECs and mTECs - (A)** Expression of exon 2 of *Foxo3* gene obtained from RNA-Seq analysis of FACS-sorted cTECs and mTECs purified from 6-week-old Foxo3^Ctr^ and Foxo3^cKO^ mice. **(B)** Principal component analysis (PCA) obtained from RNA-Seq analysis of cTECs and mTECs isolated from Foxo3^Ctr^ and Foxo3^cKO^ thymus. **(C)** Venn diagram depicts the number of differentially expressed genes detected in cTECs, mTECs and commonly detected in both cTECs and mTECs. **(D)** Venn diagrams depict the specific and common DEGs resulting from *Trp53* or *Foxo3* deficiency in cTECs (left) and mTECs (right). **(E)** Expression of cTEC-specific genes in Foxo3^Ctr^ and Foxo3^cKO^ cTECs. **(F)** Expression of mTEC-specific genes in Foxo3^Ctr^ and Foxo3^cKO^ mTECs. All data are represented as mean ± SD. **P* < 0.05; ***P* < 0.01; ****P* < 0.001.

**Supplementary figure 3. Analysis of genetic signature of mTEC subsets in Foxo3^cKO^ mTECs - (A)** Relative expression level of genes specifically upregulated in mTEC I, mTEC II, mTEC III and mTEC IV (data from (12)) in Foxo3^Ctr^ and Foxo3^cKO^ mTECs. **(B)** Relative expression level of genes specifically upregulated in mimetic mTEC subsets (as defined by Michelson et al., 2022) in Foxo3^Ctr^ and Foxo3^cKO^ mTECs. The expression of subset-specific transcription factor genes was measured in Foxo3^Ctr^ and Foxo3^cKO^ mTECs and is depicted on the right of the corresponding heatmaps. Bar graphs show mean ± SD. **P* < 0.05; ***P* < 0.01; ****P* < 0.001. **(C)** Foxo3^Ctr^ and Foxo3^cKO^ mTEC samples were analysed for the relative expression of genes coding for pro-survival and pro-apoptotic Bcl2 family members. Statistically significant differentially expressed genes are marked as * (P< 0.05), ** (P< 0.01) or *** (P< 0.001). **(D)** Relative expression level of genes specifically upregulated in proliferating and transit-amplifying mTECs (as defined by Baran-Gale et al., 2020, and Michelson et al., 2022) in Foxo3^Ctr^ and Foxo3^cKO^ mTECs.

**Supplementary figure 4. Analysis of TEC cellularity recovery following SL-TBI -** 10-week-old Foxo3^Ctr^ and Foxo3^cKO^ mice were subjected to sublethal total-body irradiation (SLTBI) and analysed at day 3 and day 21 post-irradiation. 10-week-old Foxo3^Ctr^ and Foxo3^cKO^ untreated mice (Unt.) were also analysed. The cellularity of cTECs, mTEC^lo^ and mTEC^hi^ **(A)** and total thymic cellularity **(B)** were determined at the indicated time-points. Data are representative of 2 or 3 independent experiments (n=6-9 independent samples) and represented as mean ± SEM. **P* < 0.05; ***P* < 0.01; ****P* < 0.001.

**Supplementary figure 5. T cell development in the Foxo3^cKO^ thymus - (A)** Expression of Helios and PD-1 on CD4^+^ CD8^+^ DP thymocytes (left) and on CD4^+^ CD8^-^ FoxP3^-^ thymocytes (right). **(B)** Expression of CD24 and CD62L on TCRβ^+^ CD4^+^ CD8^-^ thymocytes. Bar graphs show absolute cell numbers and percentages. Data are representative of 3 independent experiments (n=9 independent samples). **(C)** CD25 and Foxp3 expression on non-recirculatory CD44^-/lo^ SP4 thymocytes in the 10-week-old thymus of Foxo3^Ctr^ and Foxo3^cKO^ mice. All data are represented as mean ± SD. **(D)** *In vitro* suppression assay measuring CFSE labelling in conventional T cells (Tconv) from Foxo3^Ctr^ thymus (WT) on day 3 of stimulation and co-culture with Foxo3^Ctr^ or Foxo3^cKO^-derived thymic regulatory T cells (Treg) at the indicated Treg:Tconv ratios. Graphs represent the average number of divisions and precursor frequency normalized relatively to the corresponding conditions with Foxo3^Ctr^ Tregs, which were set to 1. Results are presented as mean ± SEM of 4 independent experiments. **(E)** Expression of CD24, CD44 and NK1.1 on iNKTs for the quantification of stage 0 (CD24^+^), stage 1 (CD24^-^CD44^-^NK1.1^-^), stage 2 (CD24^-^CD44^+^NK1.1^-^) and stage 3 (CD24^-^CD44^+^NK1.1^+^). Bar graphs show absolute cell numbers and percentages. Data are representative of 2 to 4 independent experiments (n=6 to 12 independent samples). Data are represented as mean ± SD. **P* < 0.05; ***P* < 0.01; ****P* < 0.001.

**Supplementary figure 6. Effects of TEC-specific *Foxo3* deletion in Aire-independent mTEC lineages and in the autoimmune syndrome of Aire^KO^ mice- (A)** TECs from 10-week-old Aire^WT^, Aire^KO^ and Aire^KO^Foxo3^cKO^ thymus were analysed for cTEC and mTEC composition (top). Total mTECs were analysed for mTEC^lo^ and mTEC^hi^ composition (bottom). Bar graphs show absolute cell numbers. Data are representative of 4 independent experiments (n=5-10 independent samples). Data are represented as mean ± SEM. **(B)** TCRβ^+^ CD4^+^ thymocytes from Aire^WT^, Aire^KO^ and Aire^KO^Foxo3^cKO^ thymus were analysed for CD25 and Foxp3 expression **(C)** Total thymocytes from Aire^WT^, Aire^KO^ and Aire^KO^Foxo3^cKO^ thymus were analysed for expression of TCRβ and reactivity with PBS57-loaded CD1d tetramer. Bar graphs show absolute cell numbers and percentages. Data are representative of 4 independent experiments (n=5-10 biologically independent samples). Data are represented as mean ± SD. **(D)** Severity scores for inflammatory lymphocytic infiltration in salivary glands, lacrimal glands, kidney and colon of 6 months-old Aire^WT^, Aire^KO^ and Aire^KO^Foxo3^cKO^ mice. Pie charts represent absent, moderate and severe lesions as light grey, dark grey and black, respectively. Bar graphs represent absent, moderate and severe lesions scored as 0-2, respectively. Data are represented as mean ± SD. **P* < 0.05; ***P* < 0.01; ****P* < 0.001.
