# Supplementary material for: Foxo3 regulates cortical and medullary thymic epithelial cell homeostasis with implications in T cell development
Source: Cell Death Dis. 2024 May 21;15(5):352. doi: 10.1038/s41419-024-06728-0 (PMC11109193; doi:10.1038/s41419-024-06728-0)
Supplement: Supplementary file 3 — Supplementary Tables [file 41419_2024_6728_MOESM3_ESM.pdf]

Table 1. Downregulated in cKO cTEC

row	baseMean	log2FoldChai	lfcSE	stat	pvalue	padj	symbol	geneName
ENSMUSG0C	7282.85778	-0.6122422	0.16278323	-3.7610891	0.00016918	0.01465312	Ckmt1	creatine kinase, mitochondrial 1, ubiquitous
ENSMUSG0C	6124.00795	-0.5827758	0.17293936	-3.3698278	0.00075215	0.04337724	Elf3	E74-like factor 3
ENSMUSG0C	2470.29307	-1.3118527	0.22662416	-5.7886708	7.09E-09	2.43E-06	Hck	hemopoietic cell kinase
ENSMUSG0C	8257.53613	-0.3953925	0.12514059	-3.1595859	0.00157994	0.07226174	Etv3	ets variant 3
ENSMUSG0C	14866.6408	-0.3886103	0.09508952	-4.0867837	4.37E-05	0.00531191	Supt5	suppressor of Ty 5, DSIF elongation factor subunit
ENSMUSG0C	108.819687	-3.0558745	0.89218921	-3.4251417	0.00061448	0.03791434	Dll3	delta like canonical Notch ligand 3
ENSMUSG0C	9576.35535	-0.5135715	0.1305447	-3.934066	8.35E-05	0.0090323	Ptpn6	protein tyrosine phosphatase, non-receptor type 6
ENSMUSG0C	5967.71001	-0.5639461	0.13565634	-4.157167	3.22E-05	0.0042022	Igf1r	insulin-like growth factor I receptor
ENSMUSG0C	6636.81785	-1.2225246	0.18661133	-6.5511811	5.71E-11	4.37E-08	Cyp2a5	cytochrome P450, family 2, subfamily a, polypeptide 5
ENSMUSG0C	1265.77993	-0.9730666	0.31238553	-3.1149542	0.00183973	0.0799762	Atp4a	ATPase, H+/K+ exchanging, gastric, alpha polypeptide
ENSMUSG0C	3469.01995	-0.8236313	0.1686576	-4.8834519	1.04E-06	0.00023019	Kit	KIT proto-oncogene receptor tyrosine kinase
ENSMUSG0C	10425.3761	-0.3997963	0.12075535	-3.3107954	0.00093031	0.05024342	Htra1	HtrA serine peptidase 1
ENSMUSG0C	912.505756	-1.1378321	0.30798772	-3.6944075	0.0002204	0.01781283	Neurl1a	neuralized E3 ubiquitin protein ligase 1A
ENSMUSG0C	967.564501	-1.2328933	0.27600898	-4.4668593	7.94E-06	0.00137699	Odad4	outer dynein arm complex subunit 4
ENSMUSG0C	4023.8416	-0.8293157	0.17697931	-4.6859473	2.79E-06	0.00056108	Hap1	huntingtin-associated protein 1
ENSMUSG0C	9594.18845	-0.9559726	0.15313644	-6.2426201	4.30E-10	2.74E-07	Atp1a2	ATPase, Na+/K+ transporting, alpha 2 polypeptide
ENSMUSG0C	8078.38783	-0.57429	0.13714147	-4.1875736	2.82E-05	0.00378459	Spib	Spi-B transcription factor (Spi-1/PU.1 related)
ENSMUSG0C	12646.5627	-0.3473662	0.10444371	-3.3258701	0.00088143	0.04794185	Hipk1	homeodomain interacting protein kinase 1
ENSMUSG0C	10268.2758	-1.7578171	0.13843631	-12.697659	6.09E-37	1.40E-32	Trpm5	transient receptor potential cation channel, subfamily M, member 5
ENSMUSG0C	1053.24832	-1.0110605	0.31284356	-3.2318406	0.00122996	0.06207366	Kcnq1	potassium voltage-gated channel, subfamily Q, member 1
ENSMUSG0C	982.977697	-1.0435994	0.33169478	-3.1462643	0.00165371	0.07413573	Spire2	spire type actin nucleation factor 2
ENSMUSG0C	7068.20404	-0.5753438	0.11484847	-5.0095906	5.45E-07	0.00013042	Ehf	ets homologous factor
ENSMUSG0C	1464.44008	-0.9639245	0.28280682	-3.4084201	0.0006534	0.03895464	Castor2	cytosolic arginine sensor for mTORC1 subunit 2
ENSMUSG0C	745.344741	-1.5442397	0.48995169	-3.1518204	0.00162256	0.07331225	Wnt11	wingless-type MMTV integration site family, member 11
ENSMUSG0C	1223.8772	-1.0544194	0.27502438	-3.8339123	0.00012612	0.01196222	Il17rb	interleukin 17 receptor B
ENSMUSG0C	3842.4745	-0.7476674	0.14214429	-5.2599188	1.44E-07	3.99E-05	Chdh	choline dehydrogenase
ENSMUSG0C	2447.17681	-0.6819448	0.19096379	-3.5710687	0.00035553	0.02565361	Cadps2	Ca2+-dependent activator protein for secretion 2
ENSMUSG0C	2611.92556	-0.7807461	0.23955598	-3.2591383	0.00111751	0.05777082	Erbb3	erb-b2 receptor tyrosine kinase 3
ENSMUSG0C	3365.9577	-0.5945651	0.18215061	-3.2641401	0.00109797	0.05755335	Acsl1	acyl-CoA synthetase long-chain family member 1
ENSMUSG0C	16778.9627	-0.4488384	0.0923581	-4.8597616	1.18E-06	0.00025449	Calm3	calmodulin 3
ENSMUSG0C	4313.31557	-1.038206	0.21289603	-4.8765868	1.08E-06	0.00023595	Krt10	keratin 10
ENSMUSG0C	25137.1716	-0.3680064	0.07793313	-4.7220792	2.33E-06	0.00048273	Cd164	CD164 antigen
ENSMUSG0C	9172.74775	-0.5468961	0.13865107	-3.9444058	8.00E-05	0.00882787	Nudt4	nudix (nucleoside diphosphate linked moiety X)-type motif 4
ENSMUSG0C	7295.95689	-0.6090242	0.17377646	-3.5046418	0.00045722	0.03068601	Ascl1	achaete-scute family bHLH transcription factor 1
ENSMUSG0C	2822.46243	-0.51756	0.16191647	-3.1964632	0.00139124	0.06707247	Peli1	pellino 1
ENSMUSG0C	2334.78129	-1.4659245	0.24793871	-5.912447	3.37E-09	1.27E-06	Pik3cg	phosphatidylinositol-4,5-bisphosphate 3-kinase catalytic subunit gamma
ENSMUSG0C	10563.2281	-0.3746146	0.0966298	-3.8768015	0.00010584	0.01065489	Acox1	acyl-Coenzyme A oxidase 1, palmitoyl
ENSMUSG0C	14581.5063	-1.0332865	0.09645078	-10.713096	8.84E-27	1.01E-22	Atp2a3	ATPase, Ca++ transporting, ubiquitous
ENSMUSG0C	7542.52743	-0.6814565	0.11351336	-6.0033155	1.93E-09	8.07E-07	Sec14l1	SEC14-like lipid binding 1
ENSMUSG0C	5011.70619	-1.1360473	0.1297541	-8.7553866	2.03E-18	5.84E-15	Pik3r5	phosphoinositide-3-kinase regulatory subunit 5
ENSMUSG0C	13170.4073	-0.3959455	0.09723313	-4.0721254	4.66E-05	0.00559838	Acly	ATP citrate lyase
ENSMUSG0C	4873.41812	-0.4407997	0.14076979	-3.1313517	0.00174004	0.07710242	Pygl	liver glycogen phosphorylase
ENSMUSG0C	3870.81047	-1.1084881	0.178585	-6.207062	5.40E-10	3.26E-07	Clmn	calmin
ENSMUSG0C	23.6414867	-8.1644188	2.57105357	-3.1755149	0.00149571	0.07006326	Slc10a1	solute carrier family 10 (sodium/bile acid cotransporter family), member 1
ENSMUSG0C	5103.10157	-0.9472691	0.17373311	-5.4524389	4.97E-08	1.58E-05	Mctp1	multiple C2 domains, transmembrane 1
ENSMUSG0C	3273.2596	-0.5992035	0.1754876	-3.4145063	0.00063898	0.03839383	Txndc16	thioredoxin domain containing 16
ENSMUSG0C	3410.47833	-1.0697552	0.15954943	-6.7048513	2.02E-11	1.60E-08	Fyb	FYN binding protein
ENSMUSG0C	23.1177063	-8.1336689	2.26461065	-3.5916412	0.0003286	0.02433033	C9	complement component 9
ENSMUSG0C	944.606761	-1.1974749	0.3146336	-3.8059346	0.00014127	0.012766	Slc7a8	solute carrier family 7 (cationic amino acid transporter, y+ system), member 8
ENSMUSG0C	1738.56793	-1.2920922	0.30236792	-4.2732449	1.93E-05	0.00279865	Dnah5	dynein, axonemal, heavy chain 5
ENSMUSG0C	7155.9501	-0.5969019	0.16663453	-3.5821023	0.00034084	0.0250747	Enpp2	ectonucleotide pyrophosphatase/phosphodiesterase 2
ENSMUSG0C	33.7160918	-8.6770783	2.01571029	-4.3047249	1.67E-05	0.00250043	Ribc2	RIB43A domain with coiled-coils 2
ENSMUSG0C	3883.9238	-0.4814488	0.14075201	-3.4205466	0.00062495	0.03798041	Vdr	vitamin D (1,25-dihydroxyvitamin D3) receptor
ENSMUSG0C	328.676531	-2.1859082	0.57661723	-3.7909172	0.00015009	0.01345727	Spag6l	sperm associated antigen 6-like
ENSMUSG0C	3149.60568	-0.8073511	0.21019154	-3.8410258	0.00012252	0.01179003	Muc13	mucin 13, epithelial transmembrane
ENSMUSG0C	2485.90429	-0.9526648	0.18672702	-5.1019119	3.36E-07	8.48E-05	Adcy5	adenylate cyclase 5
ENSMUSG0C	23977.187	-0.7395761	0.10772335	-6.8655136	6.63E-12	6.58E-09	Ivns1abp	influenza virus NS1A binding protein
ENSMUSG0C	3661.36852	-1.0567313	0.1571566	-6.7240655	1.77E-11	1.45E-08	Gnb3	guanine nucleotide binding protein (G protein), beta 3
ENSMUSG0C	7822.80523	-0.3783797	0.10883462	-3.4766481	0.00050772	0.03339193	Igf2r	insulin-like growth factor 2 receptor
ENSMUSG0C	6144.72232	-0.4682571	0.13985765	-3.3480977	0.00081368	0.04598981	Abca3	ATP-binding cassette, sub-family A (ABC1), member 3
ENSMUSG0C	14280.2828	-1.2241245	0.13307071	-9.1990529	3.61E-20	1.38E-16	Avil	advillin
ENSMUSG0C	2975.04371	-0.8643261	0.20476719	-4.2210183	2.43E-05	0.00335341	Cdhr5	cadherin-related family member 5
ENSMUSG0C	5094.73596	-0.7456097	0.16699088	-4.4649726	8.01E-06	0.00137699	Alox5	arachidonate 5-lipoxygenase
ENSMUSG0C	4926.01095	-0.4602497	0.1477077	-3.1159493	0.00183354	0.07985805	Xiap	X-linked inhibitor of apoptosis
ENSMUSG0C	3712.49995	-0.6484808	0.15695535	-4.131626	3.60E-05	0.00456785	Nrp2	neuropilin 2
ENSMUSG0C	5747.52636	-0.4475281	0.12986732	-3.4460408	0.00056886	0.03626985	Nabp1	nucleic acid binding protein 1
ENSMUSG0C	3680.46264	-1.1469571	0.20296814	-5.6509216	1.60E-08	5.31E-06	Ptpn18	protein tyrosine phosphatase, non-receptor type 18
ENSMUSG0C	2064.31338	-0.8398816	0.20632887	-4.0705965	4.69E-05	0.0056059	Vil1	villin 1
ENSMUSG0C	3436.17574	-0.8754838	0.18970819	-4.6148975	3.93E-06	0.00074605	Inpp5d	inositol polyphosphate-5-phosphatase D
ENSMUSG0C	13198.1888	-0.4823132	0.11515756	-4.1882899	2.81E-05	0.00378459	Pam	peptidylglycine alpha-amidating monooxygenase
ENSMUSG0C	2210.96301	-0.9269834	0.26140205	-3.5461979	0.00039083	0.02743358	Lypd1	Ly6/Plaur domain containing 1
ENSMUSG0C	3979.02656	-0.5114615	0.14873616	-3.4387168	0.00058448	0.03675487	Tfcp2l1	transcription factor CP2-like 1
ENSMUSG0C	434.375782	-1.2067966	0.39175064	-3.0805224	0.00206638	0.08734729	Dusp27	dual specificity phosphatase 27 (putative)
ENSMUSG0C	30081.9946	-0.5028203	0.10719341	-4.6907764	2.72E-06	0.00055284	Atp1b1	ATPase, Na+/K+ transporting, beta 1 polypeptide
ENSMUSG0C	3412.3073	-0.6339256	0.17082089	-3.7110541	0.0002064	0.01685926	Plxna2	plexin A2
ENSMUSG0C	1561.82178	-1.2759898	0.35738717	-3.5703291	0.00035653	0.02565361	Chgb	chromogranin B
ENSMUSG0C	1865.18461	-1.5412491	0.31316298	-4.9215558	8.59E-07	0.00019707	Pck1	phosphoenolpyruvate carboxykinase 1, cytosolic
ENSMUSG0C	11104.8317	-0.6567966	0.15462893	-4.2475663	2.16E-05	0.00310016	Postn	periostin, osteoblast specific factor
ENSMUSG0C	6126.6818	-1.0412576	0.14699192	-7.0837745	1.40E-12	1.61E-09	Dclk1	doublecortin-like kinase 1
ENSMUSG0C	716.095696	-0.9816232	0.32105788	-3.0574648	0.00223218	0.09132832	Chrnb2	cholinergic receptor, nicotinic, beta polypeptide 2 (neuronal)
ENSMUSG0C	150.58501	-2.0933629	0.68130773	-3.0725658	0.00212227	0.08889137	Fga	fibrinogen alpha chain
ENSMUSG0C	2978.47347	-0.7330827	0.21947992	-3.34009	0.00083751	0.04645296	Rragd	Ras-related GTP binding D
ENSMUSG0C	5641.8903	-1.2094721	0.19738917	-6.1273477	8.94E-10	4.83E-07	Col15a1	collagen, type XV, alpha 1
ENSMUSG0C	6748.17083	-0.7366064	0.17439615	-4.2237537	2.40E-05	0.00334233	Slc5a9	solute carrier family 5 (sodium/glucose cotransporter), member 9
ENSMUSG0C	2877.92782	-0.9607469	0.2367528	-4.0580169	4.95E-05	0.00586594	Tspan1	tetraspanin 1
ENSMUSG0C	2630.32247	-0.9036973	0.18892063	-4.7834762	1.72E-06	0.0003628	Gnat3	guanine nucleotide binding protein, alpha transducing 3
ENSMUSG0C	1119.55873	-0.889133	0.24935992	-3.5656612	0.00036294	0.02603301	Adgrb2	adhesion G protein-coupled receptor B2
ENSMUSG0C	3395.26643	-0.5020878	0.15037911	-3.3388137	0.00084137	0.04653485	Inpp5b	inositol polyphosphate-5-phosphatase B
ENSMUSG0C	3540.4327	-0.6064982	0.1834192	-3.3066234	0.00094428	0.05075878	Espn	espin
ENSMUSG0C	2501.98291	-0.7484979	0.19045153	-3.9301229	8.49E-05	0.00910638	Fam126a	family with sequence similarity 126, member A
ENSMUSG0C	4954.8338	-0.6869089	0.15392735	-4.4625529	8.10E-06	0.00137699	Sel1l3	sel-1 suppressor of lin-12-like 3 (C. elegans)
ENSMUSG0C	37.9454034	-8.8469528	2.27090521	-3.8957825	9.79E-05	0.01012022	Klb	klotho beta
ENSMUSG0C	7343.79361	-0.6860227	0.20454812	-3.3538451	0.00079697	0.04527932	Sparcl1	SPARC-like 1
ENSMUSG0C	2501.27408	-0.6061201	0.1943834	-3.1181681	0.00181979	0.07956121	Bmp3	bone morphogenetic protein 3
ENSMUSG0C	19756.4926	-0.3636957	0.087044	-4.1782977	2.94E-05	0.00389669	Atp2a2	ATPase, Ca++ transporting, cardiac muscle, slow twitch 2
ENSMUSG0C	31.0080387	-8.5553218	2.20418333	-3.8814021	0.00010386	0.01050135	Gngt1	guanine nucleotide binding protein (G protein), gamma transducing activity polypeptide 1
ENSMUSG0C	23525.7647	-0.6909217	0.08489499	-8.1385445	4.00E-16	9.18E-13	Ahcyl2	S-adenosylhomocysteine hydrolase-like 2
ENSMUSG0C	7750.03093	-0.4761828	0.130885	-3.6381771	0.00027457	0.0211487	Aoc1	amine oxidase, copper-containing 1
ENSMUSG0C	1908.91044	-1.2000635	0.2260673	-5.3084347	1.11E-07	3.20E-05	Hpgds	hematopoietic prostaglandin D synthase
ENSMUSG0C	17686.867	-0.3948547	0.09008661	-4.3830568	1.17E-05	0.00187838	Anxa4	annexin A4
ENSMUSG0C	137.700902	-2.2790117	0.70850772	-3.2166364	0.00129703	0.06471892	Clec4e	C-type lectin domain family 4, member e
ENSMUSG0C	2540.4603	-0.759917	0.19897405	-3.8191764	0.0001339	0.01231137	Bcl2l14	BCL2-like 14 (apoptosis facilitator)
ENSMUSG0C	2182.74937	-0.9990138	0.24132227	-4.1397495	3.48E-05	0.00443357	Emp1	epithelial membrane protein 1
ENSMUSG0C	34.0581754	-8.6909987	1.96354401	-4.4261797	9.59E-06	0.00158387	Plcz1	phospholipase C, zeta 1
ENSMUSG0C	427.001128	-1.7780527	0.45951392	-3.8694207	0.00010909	0.01093468	Abcc9	ATP-binding cassette, sub-family C (CFTR/MRP), member 9
ENSMUSG0C	10633.3196	-1.095696	0.15330845	-7.1470032	8.87E-13	1.07E-09	Irag2	inositol 1,4,5-triphosphate receptor associated 2
ENSMUSG0C	7668.61344	-0.5087725	0.12935293	-3.9332121	8.38E-05	0.0090323	Itpr2	inositol 1,4,5-triphosphate receptor 2
ENSMUSG0C	9354.86304	-0.4693863	0.11468953	-4.0926686	4.26E-05	0.00523423	Cyfip1	cytoplasmic FMR1 interacting protein 1
ENSMUSG0C	12180.6862	-0.587067	0.15551302	-3.7750345	0.00015999	0.01401583	Herc2	HECT and RLD domain containing E3 ubiquitin protein ligase 2

ENSMUSG0(	2812.00821	-0.6471891	0.16562468	-3.9075642	9.32E-05	0.00977643	Psd3	pleckstrin and Sec7 domain containing 3
ENSMUSG0(	1950.2786	-0.8213354	0.24477787	-3.3554314	0.00079241	0.04524442	Apba2	amyloid beta (A4) precursor protein-binding, family A, member 2
ENSMUSG0(	5559.67997	-0.4513748	0.13664383	-3.3032944	0.00095556	0.05124526	Furin	furin (paired basic amino acid cleaving enzyme)
ENSMUSG0(	24912.2424	-0.4822766	0.10017822	-4.8141858	1.48E-06	0.00031705	Iqgap1	IQ motif containing GTPase activating protein 1
ENSMUSG0(	36.5421725	-7.7827289	2.17745205	-3.5742366	0.00035125	0.0255945	Plin1	perilipin 1
ENSMUSG0(	4715.4181	-0.858363	0.17158529	-5.0025442	5.66E-07	0.00013388	Ctsc	cathepsin C
ENSMUSG0(	2910.38895	-0.9295574	0.17523314	-5.3046896	1.13E-07	3.20E-05	Xylt1	xylosyltransferase 1
ENSMUSG0(	14610.1102	-0.2759457	0.08804988	-3.1339698	0.00172459	0.07671401	Mvp	major vault protein
ENSMUSG0(	883.051472	-1.2169971	0.36280183	-3.3544403	0.00079526	0.04527932	Klk7	kallikrein related-peptidase 7 (chymotryptic, stratum corneum)
ENSMUSG0(	12565.9399	-0.3316647	0.09127626	-3.6336359	0.00027946	0.02131008	Il4ra	interleukin 4 receptor, alpha
ENSMUSG0(	212.678834	-1.9361829	0.63608745	-3.0438942	0.00233537	0.09354939	Tead2	TEA domain family member 2
ENSMUSG0(	20021.3945	-0.3973811	0.09267101	-4.288084	1.80E-05	0.00263478	Nucb1	nucleobindin 1
ENSMUSG0(	414.154565	-2.3003401	0.57477932	-4.0021275	6.28E-05	0.00709796	Vwa3a	von Willebrand factor A domain containing 3A
ENSMUSG0(	3763.84688	-0.5586274	0.14372899	-3.8866714	0.00010163	0.01032155	Trim30a	tripartite motif-containing 30A
ENSMUSG0(	8264.49087	-1.1994945	0.1712632	-7.0038074	2.49E-12	2.72E-09	Gp2	glycoprotein 2 (zymogen granule membrane)
ENSMUSG0(	5298.18191	-0.519382	0.1565459	-3.3177619	0.00090742	0.04923869	Swap70	SWA-70 protein
ENSMUSG0(	464.625804	-1.6966434	0.53111918	-3.1944684	0.00140089	0.06712849	Trim66	tripartite motif-containing 66
ENSMUSG0(	4246.57634	-0.4821197	0.14651743	-3.2905282	0.00099999	0.05337879	Efnb1	ephrin B1
ENSMUSG0(	8917.24459	-0.3376306	0.1092927	-3.0892323	0.00200674	0.08567975	Sh3bgrl	SH3-binding domain glutamic acid-rich protein like
ENSMUSG0(	601.82955	-2.1580242	0.35354814	-6.1039048	1.04E-09	5.05E-07	Srpx2	sushi-repeat-containing protein, X-linked 2
ENSMUSG0(	437.819671	-1.5229976	0.46673836	-3.2630649	0.00110214	0.05755335	Chrdl1	chordin-like 1
ENSMUSG0(	16320.3406	-0.3528137	0.08948019	-3.9429257	8.05E-05	0.00884005	Rbbp7	retinoblastoma binding protein 7, chromatin remodeling factor
ENSMUSG0(	3710.37325	-1.2273922	0.17832307	-6.882969	5.86E-12	6.12E-09	Bmx	BMX non-receptor tyrosine kinase
ENSMUSG0(	2547.57264	-0.7145724	0.18643438	-3.832836	0.00012667	0.01196526	L1cam	L1 cell adhesion molecule
ENSMUSG0(	4536.52734	-0.4704396	0.15217198	-3.0914998	0.00199148	0.08544013	Mcf2l	mcf.2 transforming sequence-like
ENSMUSG0(	659.973827	-1.9101364	0.61203501	-3.1209593	0.00180263	0.07931883	Car7	carbonic anhydrase 7
ENSMUSG0(	1271.15695	-1.6646533	0.24514119	-6.7905902	1.12E-11	9.86E-09	Agt	angiotensinogen (serpin peptidase inhibitor, clade A, member 8)
ENSMUSG0(	2662.16697	-0.755459	0.16577565	-4.5571165	5.19E-06	0.00095229	Pou2f3	POU domain, class 2, transcription factor 3
ENSMUSG0(	5633.95598	-0.6221484	0.18391956	-3.3827203	0.00071772	0.04191796	Apoa4	apolipoprotein A-IV
ENSMUSG0(	1202.63329	-0.8810083	0.28014557	-3.1448233	0.00166187	0.07421195	Cln6	ceroid-lipofuscinosis, neuronal 6
ENSMUSG0(	10365.6124	-0.5008633	0.14992534	-3.3407514	0.00083552	0.04645296	Ptgs2	prostaglandin-endoperoxide synthase 2
ENSMUSG0(	10428.7376	-0.4766128	0.10499769	-4.5392699	5.64E-06	0.00102022	Kdm4a	lysine (K)-specific demethylase 4A
ENSMUSG0(	785.639446	-1.1645933	0.32474375	-3.5861915	0.00033554	0.02476434	Adra2a	adrenergic receptor, alpha 2a
ENSMUSG0(	1590.06264	-1.0041592	0.2638087	-3.8063916	0.00014101	0.012766	Ano7	anoctamin 7
ENSMUSG0(	4392.58737	-1.2066935	0.14984441	-8.0529767	8.08E-16	1.69E-12	Vav1	vav 1 oncogene
ENSMUSG0(	2383.80055	-0.6566275	0.17970904	-3.6538365	0.00025835	0.02010144	Faah	fatty acid amide hydrolase
ENSMUSG0(	4049.96189	-0.6381277	0.16598106	-3.8445813	0.00012076	0.01179003	Plcg2	phospholipase C, gamma 2
ENSMUSG0(	4532.89384	-0.8385038	0.14009061	-5.9854388	2.16E-09	8.85E-07	Myo15b	myosin XVB
ENSMUSG0(	1504.7115	-0.97719	0.24502024	-3.9882012	6.66E-05	0.0074908	Inpp5j	inositol polyphosphate 5-phosphatase J
ENSMUSG0(	43.2052656	-9.0348812	1.84063582	-4.9085654	9.17E-07	0.0002085	B3galt1	UDP-Gal:betaGlcNAc beta 1,3-galactosyltransferase, polypeptide 1
ENSMUSG0(	1768.55713	-1.3270205	0.21214614	-6.2552186	3.97E-10	2.68E-07	Cdhr2	cadherin-related family member 2
ENSMUSG0(	2165.15353	-0.6736613	0.1881908	-3.5796718	0.00034403	0.02522821	Egln3	egl-9 family hypoxia-inducible factor 3
ENSMUSG0(	30.8491896	-8.5481412	2.44214643	-3.5002575	0.00046481	0.03101384	Plk5	polo like kinase 5
ENSMUSG0(	1843.16581	-0.7356678	0.24302659	-3.0271084	0.00246905	0.09771068	Pcdh17	protocadherin 17
ENSMUSG0(	8021.87344	-0.5552454	0.15118714	-3.6725707	0.00024012	0.01907106	Syt1	synaptotagmin I
ENSMUSG0(	1166.69933	-0.9456277	0.29967385	-3.1555229	0.00160211	0.07267423	1810046K07I	RIKEN cDNA 1810046K07 gene
ENSMUSG0(	1607.34586	-1.0595479	0.27467015	-3.8575284	0.00011454	0.01138105	Lum	lumican
ENSMUSG0(	468.845149	-1.6584821	0.41341293	-4.0116842	6.03E-05	0.00685035	Mag	myelin-associated glycoprotein
ENSMUSG0(	15.0926872	-20.934337	3.36844983	-6.2148283	5.14E-10	3.19E-07	Stpg3	sperm tail PG rich repeat containing 3
ENSMUSG0(	3184.90209	-0.6377875	0.20224105	-3.1536006	0.0016127	0.07301031	Lypd8l	LY6/PLAUR domain containing 8 like
ENSMUSG0(	16146.9671	-0.3347865	0.10741902	-3.116641	0.00182924	0.07982241	Clic4	chloride intracellular channel 4 (mitochondrial)
ENSMUSG0(	597.032817	-1.1989996	0.36503878	-3.2845814	0.00102134	0.05428835	Rims2	regulating synaptic membrane exocytosis 2
ENSMUSG0(	6937.21472	-1.379159	0.18388649	-7.5000561	6.38E-14	9.15E-11	Cdkn1c	cyclin-dependent kinase inhibitor 1C (P57)
ENSMUSG0(	1218.34745	-1.3862087	0.40356696	-3.4348915	0.00059279	0.03712007	Muc5ac	mucin 5, subtypes A and C, tracheobronchial/gastric
ENSMUSG0(	5841.97158	-0.4890175	0.14115839	-3.4643178	0.00053158	0.03457869	Atp6v0a2	ATPase, H+ transporting, lysosomal V0 subunit A2
ENSMUSG0(	817.344901	-1.3001023	0.29157348	-4.4589181	8.24E-06	0.00139025	AW551984	expressed sequence AW551984
ENSMUSG0(	1457.99545	-0.7204729	0.23551046	-3.059197	0.00221931	0.09129006	Spon1	spondin 1, (f-spondin) extracellular matrix protein
ENSMUSG0(	8593.9695	-0.3788152	0.1243106	-3.0473285	0.00230885	0.09297382	Trp53inp2	transformation related protein 53 inducible nuclear protein 2
ENSMUSG0(	5452.61962	-0.4540549	0.15009067	-3.0252042	0.00248465	0.09774125	Atf5	activating transcription factor 5
ENSMUSG0(	6753.33228	-0.6015471	0.12999206	-4.6275677	3.70E-06	0.00071364	Ascc3	activating signal cointegrator 1 complex subunit 3
ENSMUSG0(	965.615225	-0.9531594	0.28031127	-3.4003606	0.00067297	0.03990504	Klk1b16	kallikrein 1-related peptidase b16
ENSMUSG0(	3435.40872	-1.2160071	0.16069738	-7.5670623	3.82E-14	6.26E-11	Adgrg6	adhesion G protein-coupled receptor G6
ENSMUSG0(	14675.6133	-0.6615388	0.11097847	-5.9609656	2.51E-09	9.92E-07	Abhd2	abhydrolase domain containing 2
ENSMUSG0(	427.901875	-1.3478624	0.41343791	-3.2601325	0.0011136	0.05777082	Gimap3	GTPase, IMAP family member 3
ENSMUSG0(	5867.77387	-0.4432445	0.14366156	-3.0853382	0.00203321	0.08658298	Megf9	multiple EGF-like-domains 9
ENSMUSG0(	361.80218	-1.9253355	0.49358663	-3.9007043	9.59E-05	0.00996153	Ccdc81	coiled-coil domain containing 81
ENSMUSG0(	4615.18589	-0.643967	0.16824338	-3.827592	0.0001294	0.01207393	Strip2	striatin interacting protein 2
ENSMUSG0(	10568.5417	-0.3425736	0.09726244	-3.5221567	0.00042805	0.02915506	Hnrnpu	heterogeneous nuclear ribonucleoprotein U
ENSMUSG0(	678.107722	-1.5201484	0.47025047	-3.2326357	0.00122654	0.06207366	Ccdc40	coiled-coil domain containing 40
ENSMUSG0(	5178.94448	-0.9175031	0.15730105	-5.8327848	5.45E-09	1.99E-06	Plcb2	phospholipase C, beta 2
ENSMUSG0(	1622.12974	-0.9028704	0.29522672	-3.0582274	0.00222651	0.09132832	Cacna2d1	calcium channel, voltage-dependent, alpha2/delta subunit 1
ENSMUSG0(	266.771631	-1.9862863	0.56000505	-3.5469079	0.00038978	0.02743358	Hmx3	H6 homeobox 3
ENSMUSG0(	13998.4179	-0.5310548	0.10636746	-4.9926433	5.96E-07	0.00013949	Ltbp4	latent transforming growth factor beta binding protein 4
ENSMUSG0(	943.99358	-0.9458597	0.29238315	-3.2350008	0.00121642	0.06190816	Cyp2s1	cytochrome P450, family 2, subfamily s, polypeptide 1
ENSMUSG0(	1450.27585	-1.4165071	0.24426498	-5.7990593	6.67E-09	2.32E-06	Entpd3	ectonucleoside triphosphate diphosphohydrolase 3
ENSMUSG0(	505.692151	-1.4548267	0.39449506	-3.6878197	0.00022618	0.01808919	Coro2b	coronin, actin binding protein, 2B
ENSMUSG0(	880.001408	-1.4936185	0.35213412	-4.2416182	2.22E-05	0.00314419	Tmem45b	transmembrane protein 45b
ENSMUSG0(	11218.7485	-0.7570789	0.17078567	-4.4329182	9.30E-06	0.00154627	Rptn	repetin
ENSMUSG0(	2554.62501	-0.5700184	0.18875036	-3.0199595	0.00252808	0.09851805	Rassf4	Ras association (RalGDS/AF-6) domain family member 4
ENSMUSG0(	2650.91739	-0.776485	0.20239336	-3.8365141	0.00012479	0.01193489	Osbpl6	oxysterol binding protein-like 6
ENSMUSG0(	36.6370743	-8.796745	1.88327784	-4.6709757	3.00E-06	0.00059316	Dnaaf6b	dynein axonemal assembly factor 6B
ENSMUSG0(	477.475383	-1.575718	0.51765007	-3.043983	0.00233468	0.09354939	Dnali1	dynein, axonemal, light intermediate polypeptide 1
ENSMUSG0(	825.389841	-1.0163728	0.30011576	-3.3866026	0.00070764	0.04143473	Fgf11	fibroblast growth factor 11
ENSMUSG0(	2073.0661	-1.2344749	0.22899675	-5.3907966	7.01E-08	2.15E-05	Hepacam2	HEPACAM family member 2
ENSMUSG0(	1690.90318	-0.7147163	0.23092637	-3.0949966	0.00196815	0.08459736	Krt84	keratin 84
ENSMUSG0(	2343.6798	-0.6745825	0.20156206	-3.3467731	0.00081758	0.04599495	Fads6	fatty acid desaturase domain family, member 6
ENSMUSG0(	14.2656906	-20.854088	3.42935205	-6.0810578	1.19E-09	5.40E-07	Krtap5-4	keratin associated protein 5-4
ENSMUSG0(	4311.42431	-1.0890845	0.18333359	-5.9404527	2.84E-09	1.09E-06	Fndc7	fibronectin type III domain containing 7
ENSMUSG0(	1336.91695	-1.0568949	0.23834493	-4.4343082	9.24E-06	0.00154627	Slitrk6	SLIT and NTRK-like family, member 6
ENSMUSG0(	368.869408	-2.5028558	0.59538473	-4.2037621	2.63E-05	0.00358661	Kcnrg	potassium channel regulator
ENSMUSG0(	6264.6907	-0.6774903	0.12991507	-5.2148711	1.84E-07	4.91E-05	Gjb2	gap junction protein, beta 2
ENSMUSG0(	1199.23029	-0.8835865	0.25466142	-3.4696519	0.00052113	0.03409253	Camk2n1	calcium/calmodulin-dependent protein kinase II inhibitor 1
ENSMUSG0(	2656.79323	-1.9209904	0.20822195	-9.2256861	2.82E-20	1.29E-16	Sh2d7	SH2 domain containing 7
ENSMUSG0(	1145.78306	-1.337119	0.358058	-3.7343644	0.00018819	0.01586517	Cfap65	cilia and flagella associated protein 65
ENSMUSG0(	2448.05801	-1.2814317	0.23586701	-5.4328568	5.55E-08	1.74E-05	Ptgs1	prostaglandin-endoperoxide synthase 1
ENSMUSG0(	863.84219	-1.2353546	0.38328566	-3.2230649	0.00126827	0.06342169	Dmbt1	deleted in malignant brain tumors 1
ENSMUSG0(	268551.43	-0.6440433	0.08255223	-7.801647	6.11E-15	1.17E-11	Fcgbp	Fc fragment of IgG binding protein
ENSMUSG0(	449.937393	-1.3398617	0.42805011	-3.1301516	0.00174716	0.07726896	Cd300lf	CD300 molecule like family member F
ENSMUSG0(	2318.53869	-0.5877635	0.19428877	-3.0252058	0.00248464	0.09774125	Ccr4	chemokine (C-C motif) receptor 4
ENSMUSG0(	3561.15404	-0.9942783	0.22637764	-4.3921222	1.12E-05	0.00182728	Col6a3	collagen, type VI, alpha 3
ENSMUSG0(	8248.13124	-0.4669113	0.10749735	-4.3434683	1.40E-05	0.00220491	Sephs2	selenophosphate synthetase 2
ENSMUSG0(	195.060583	-2.3487236	0.67159473	-3.4972336	0.00047011	0.03127663	Rtl5	retrotransposon Gag like 5
ENSMUSG0(	1570.15195	-2.0997256	0.27807041	-7.5510576	4.32E-14	6.61E-11	Cyp2g1	cytochrome P450, family 2, subfamily g, polypeptide 1
ENSMUSG0(	5253.34653	-0.4900397	0.13693331	-3.5786739	0.00034534	0.02524406	Prkca	protein kinase C, alpha
ENSMUSG0(	3038.72342	-1.9088656	0.20800588	-9.1769791	4.43E-20	1.45E-16	Rgs13	regulator of G-protein signaling 13
ENSMUSG0(	2268.75964	-0.8691018	0.24776085	-3.5078253	0.00045179	0.03041007	Pcdh1	protocadherin 1
ENSMUSG0(	16.8011205	-7.6717212	2.48355553	-3.0890073	0.00200826	0.08567975	Kctd16	potassium channel tetramerisation domain containing 16
ENSMUSG0(	3759.89232	-0.6042399	0.16339788	-3.6979663	0.00021733	0.01762707	Klf13	Kruppel-like factor 13
ENSMUSG0(	2028.41698	-2.1016711	0.35684391	-5.8896088	3.87E-09	1.43E-06	Cd177	CD177 antigen
ENSMUSG0(	465.043055	-2.5168564	0.73905254	-3.4055176	0.00066039	0.03926911	Dnah3	dynein, axonemal, heavy chain 3
ENSMUSG0(	10331.9186	-0.4612753	0.10656308	-4.3286591	1.50E-05	0.00234245	Ppp6r1	protein phosphatase 6, regulatory subunit 1

ENSMUSG0(	55.3283424	-5.8381436	1.54235483	-3.7852143	0.00015358	0.01371608	Doc2a	double C2, alpha
ENSMUSG0(	1489.31977	-0.698169	0.22736977	-3.0706323	0.00213606	0.0891473	Cemip	cell migration inducing protein, hyaluronan binding
ENSMUSG0(	4454.3063	-0.7019703	0.18417535	-3.8114238	0.00013817	0.012635	Tchh	trichohyalin
ENSMUSG0(	2147.06359	-0.7777763	0.22011943	-3.5334286	0.00041021	0.02841029	Robo2	roundabout guidance receptor 2
ENSMUSG0(	4885.2727	-1.6760625	0.16683902	-10.045986	9.57E-24	5.49E-20	Sh2d6	SH2 domain containing 6
ENSMUSG0(	5769.6783	-0.5427151	0.13025466	-4.1665699	3.09E-05	0.00405569	Sae1	SUMO1 activating enzyme subunit 1
ENSMUSG0(	986.573101	-1.4899859	0.43446958	-3.4294366	0.00060484	0.03758911	Dnah6	dynein, axonemal, heavy chain 6
ENSMUSG0(	1135.6124	-0.827174	0.24194083	-3.4189102	0.00062872	0.03798041	Glis3	GLIS family zinc finger 3
ENSMUSG0(	29.0376582	-7.5233374	2.34949815	-3.202104	0.00136428	0.06645301	Dspp	dentin sialophosphoprotein
ENSMUSG0(	941.142464	-2.3283262	0.38293375	-6.0802324	1.20E-09	5.40E-07	Nrgn	neurogranin
ENSMUSG0(	1500.85067	-0.9305676	0.21951348	-4.2392275	2.24E-05	0.00315837	Klk1b26	kallikrein 1-related petidase b26
ENSMUSG0(	2604.89666	-0.9378009	0.23119042	-4.0564002	4.98E-05	0.00586594	Mill1	MHC I like leukocyte 1
ENSMUSG0(	3783.97637	-0.8578751	0.15257636	-5.6225951	1.88E-08	6.08E-06	Spock3	sparc/osteonectin, cwcv and kazal-like domains proteoglycan 3
ENSMUSG0(	1986.72823	-1.0440717	0.24173757	-4.3190295	1.57E-05	0.00239348	Dedd2	death effector domain-containing DNA binding protein 2
ENSMUSG0(	222.007294	-2.5019967	0.5885777	-4.2509199	2.13E-05	0.00307331	Sec14l3	SEC14-like lipid binding 3
ENSMUSG0(	382.383601	-1.531932	0.47918132	-3.1969776	0.00138876	0.06707247	Smyd1	SET and MYND domain containing 1
ENSMUSG0(	27.4223819	-8.3783585	2.48178783	-3.3759367	0.00073565	0.0427477	Them7	thioesterase superfamily member 7
ENSMUSG0(	21.8667718	-8.0516349	2.63186874	-3.0592844	0.00221866	0.09129006	Apobec4	apolipoprotein B mRNA editing enzyme, catalytic polypeptide-like 4 (putative)
ENSMUSG0(	1739.97706	-1.2747559	0.21902503	-5.8201379	5.88E-09	2.08E-06	Fut2	fucosyltransferase 2
ENSMUSG0(	1485.23192	-0.8233052	0.23398892	-3.5185651	0.00043389	0.02946455	B4galt6	UDP-Gal:betaGlcNAc beta 1,4-galactosyltransferase, polypeptide 6
ENSMUSG0(	5650.76309	-0.5056463	0.13721329	-3.6851114	0.0002286	0.01821915	Cebpg	CCAAT/enhancer binding protein (C/EBP), gamma
ENSMUSG0(	2494.58526	-0.6350878	0.19973478	-3.1796555	0.0014745	0.06921115	Myh4	myosin, heavy polypeptide 4, skeletal muscle
ENSMUSG0(	7683.90579	-0.4562365	0.13344605	-3.4188831	0.00062879	0.03798041	Ero1b	endoplasmic reticulum oxidoreductase 1 beta
ENSMUSG0(	4341.01368	-0.4722353	0.15504342	-3.045826	0.00232042	0.09327605	Trim12c	tripartite motif-containing 12C
ENSMUSG0(	3545.72663	-0.4928727	0.16127591	-3.0560836	0.00224249	0.09158686	Sphk2	sphingosine kinase 2
ENSMUSG0(	713.355372	-1.3298008	0.35183273	-3.7796393	0.00015706	0.01382302	Saa2	serum amyloid A 2
ENSMUSG0(	23.8592186	-8.178005	2.56237219	-3.1915758	0.00141499	0.06752237	Zscan5b	zinc finger and SCAN domain containing 5B
ENSMUSG0(	5318.35877	-0.4239199	0.13781866	-3.0759249	0.00209851	0.08854233	Skap2	src family associated phosphoprotein 2
ENSMUSG0(	42.1734818	-8.9993325	2.23299232	-4.0301672	5.57E-05	0.00639668	Tas2r125	taste receptor, type 2, member 125
ENSMUSG0(	656.393051	-1.1824738	0.36153497	-3.2707038	0.0010728	0.05660696	Hydin	HYDIN, axonemal central pair apparatus protein
ENSMUSG0(	2400.75045	-1.306016	0.25939488	-5.0348565	4.78E-07	0.00011554	Alox5ap	arachidonate 5-lipoxygenase activating protein
ENSMUSG0(	4636.05349	-0.8358225	0.21402781	-3.9052051	9.41E-05	0.00982237	Dmkn	dermokine
ENSMUSG0(	2247.44135	-0.7271535	0.200015	-3.635495	0.00027745	0.0212275	Prcp	prolylcarboxypeptidase (angiotensinase C)
ENSMUSG0(	566.880591	-1.8469642	0.35941521	-5.1388036	2.76E-07	7.21E-05	Tmem236	transmembrane protein 236
ENSMUSG0(	37.7509846	-5.6011803	1.74400577	-3.2116754	0.00131963	0.06542022	Cfd	complement factor D (adipsin)
ENSMUSG0(	23.3519195	-8.1476083	2.56228874	-3.1798166	0.00147368	0.06921115	Cd200r4	CD200 receptor 4
ENSMUSG0(	2760.75605	-0.75638	0.18269316	-4.140166	3.47E-05	0.00443357	Nectin2	nectin cell adhesion molecule 2
ENSMUSG0(	6357.00419	-0.8320271	0.16368606	-5.0830666	3.71E-07	9.27E-05	Serpinb2	serine (or cysteine) peptidase inhibitor, clade B, member 2
ENSMUSG0(	13357.1082	-0.7250933	0.10775336	-6.7291937	1.71E-11	1.45E-08	Fabp4	fatty acid binding protein 4, adipocyte
ENSMUSG0(	113.242441	-3.7265427	1.1183869	-3.3320693	0.00086203	0.04744874	Prss1	protease, serine 1 (trypsin 1)
ENSMUSG0(	2795.35354	-1.2120762	0.23960685	-5.0586042	4.22E-07	0.00010416	Chil4	chitinase-like 4
ENSMUSG0(	2640.65961	-1.6039788	0.23610483	-6.7935026	1.09E-11	9.86E-09	Klk1	kallikrein 1
ENSMUSG0(	21.1303638	-8.0023453	2.64591228	-3.0244182	0.00249112	0.09774125	NA	NA
ENSMUSG0(	1719.15005	-0.7147772	0.21908364	-3.2625765	0.00110404	0.05755335	Ethe1	ethylmalonic encephalopathy 1
ENSMUSG0(	1316.83508	-1.2371932	0.23399615	-5.2872372	1.24E-07	3.48E-05	Gimap8	GTPase, IMAP family member 8
ENSMUSG0(	783.267082	-0.9378539	0.30062997	-3.1196289	0.00181079	0.07931883	Zfp428	zinc finger protein 428
ENSMUSG0(	244.751712	-3.25413	0.93345194	-3.4861248	0.00049007	0.03232365	Ccdc146	coiled-coil domain containing 146
ENSMUSG0(	14.4724398	-20.873332	3.41360406	-6.1147491	9.67E-10	4.83E-07	NA	NA
ENSMUSG0(	18.2801807	-7.7930292	2.42349286	-3.2156188	0.00130164	0.06476828	Mir421	microRNA 421
ENSMUSG0(	4238.47298	-0.4791126	0.15603121	-3.07062	0.00213615	0.0891473	Trim12a	tripartite motif-containing 12A
ENSMUSG0(	19.965549	-7.9211288	2.31455167	-3.4223167	0.0006209	0.03798041	Serpina1a	serine (or cysteine) peptidase inhibitor, clade A, member 1A
ENSMUSG0(	5666.25058	-0.5760324	0.13340299	-4.3179871	1.57E-05	0.00239348	Akap13	A kinase (PRKA) anchor protein 13
ENSMUSG0(	1994.57208	-1.0830552	0.19222108	-5.6344246	1.76E-08	5.76E-06	Fxyd6	FXYD domain-containing ion transport regulator 6
ENSMUSG0(	7240.71326	-0.9159735	0.12622558	-7.2566396	3.97E-13	5.06E-10	Rnasel	ribonuclease L (2', 5'-oligoisoadenylate synthetase-dependent)
ENSMUSG0(	881.908061	-0.9032195	0.29602184	-3.0511921	0.00227935	0.09227135	Serpinb7	serine (or cysteine) peptidase inhibitor, clade B, member 7
ENSMUSG0(	2253.56234	-0.860417	0.19135598	-4.4964211	6.91E-06	0.00121085	Tspan6	tetraspanin 6
ENSMUSG0(	2337.67625	-0.7033236	0.1982112	-3.5483547	0.00038765	0.02737734	Krt77	keratin 77
ENSMUSG0(	24.3969235	-8.2102575	2.13301647	-3.8491299	0.00011854	0.01167728	4930444P10l	RIKEN cDNA 4930444P10 gene
ENSMUSG0(	2409.57517	-0.7888104	0.20097463	-3.9249254	8.68E-05	0.0092191	Sptbn2	spectrin beta, non-erythrocytic 2
ENSMUSG0(	4398.75177	-0.7274628	0.19352269	-3.759057	0.00017055	0.01465387	Lyz1	lysozyme 1
ENSMUSG0(	545.434306	-1.3188378	0.38110389	-3.460573	0.00053903	0.03494997	Cntnap5a	contactin associated protein-like 5A
ENSMUSG0(	2689.60587	-0.817568	0.25500456	-3.2060919	0.00134551	0.06606295	Pnma8b	PNMA family member 8B
ENSMUSG0(	579.368178	-1.9479946	0.48530288	-4.013977	5.97E-05	0.00681786	Gm4952	predicted gene 4952
ENSMUSG0(	5390.13332	-0.5827706	0.12792957	-4.555402	5.23E-06	0.00095247	Csf2rb	colony stimulating factor 2 receptor, beta, low-affinity (granulocyte-macrophage)
ENSMUSG0(	3434.2984	-0.5754587	0.15278084	-3.7665633	0.00016551	0.0144447	Csf2rb2	colony stimulating factor 2 receptor, beta 2, low-affinity (granulocyte-macrophage)
ENSMUSG0(	616.891918	-1.2599657	0.40136737	-3.1391832	0.00169419	0.07550846	Gm94	predicted gene 94
ENSMUSG0(	14.885938	-20.917479	3.38316547	-6.1828128	6.30E-10	3.61E-07	Nutm2	NUT family member 2
ENSMUSG0(	73.7077308	-4.6192818	1.2627657	-3.6580672	0.00025412	0.01983985	Prr16	proline rich 16
ENSMUSG0(	1316.64509	-1.1019916	0.28821428	-3.823515	0.00013156	0.01217643	Omp	olfactory marker protein
ENSMUSG0(	5650.19286	-0.4998199	0.12702576	-3.9347914	8.33E-05	0.0090323	Rbm15b	RNA binding motif protein 15B
ENSMUSG0(	4327.26825	-0.9367714	0.22887211	-4.0929906	4.26E-05	0.00523423	Saa1	serum amyloid A 1
ENSMUSG0(	720.395537	-1.6308131	0.44474386	-3.6668591	0.00024555	0.01936792	Malrd1	MAM and LDL receptor class A domain containing 1
ENSMUSG0(	643.563499	-12.931599	3.90683554	-3.3099932	0.00093298	0.05026936	Igkv4-91	immunoglobulin kappa chain variable 4-91
ENSMUSG0(	556.601782	-12.722164	3.90684746	-3.256376	0.00112844	0.05819156	Igkv8-27	immunoglobulin kappa chain variable 8-27
ENSMUSG0(	25.4042109	-6.7077156	2.01153409	-3.3346268	0.00085414	0.04712755	NA	NA
ENSMUSG0(	27.5148509	-8.3833351	2.44988339	-3.4219323	0.00062178	0.03798041	Snord89	small nucleolar RNA, C/D box 89
ENSMUSG0(	9962.35606	-0.4104727	0.10964828	-3.7435402	0.00018145	0.01536797	Ctdsp2	CTD (carboxy-terminal domain, RNA polymerase II, polypeptide A) small phosphatase 2
ENSMUSG0(	994.327484	-0.9328145	0.30758141	-3.0327401	0.00242344	0.09632184	Smim24	small integral membrane protein 24
ENSMUSG0(	16.5846306	-7.6531549	2.45937269	-3.1118321	0.0018593	0.08067403	Nkain1	Na+/K+ transporting ATPase interacting 1
ENSMUSG0(	2493.39776	-0.6597421	0.21710359	-3.0388357	0.00237494	0.0948036	Serpina3j	serine (or cysteine) peptidase inhibitor, clade A (alpha-1 antiproteinase, antitrypsin), member 3J
ENSMUSG0(	316.001518	-1.7341761	0.47931751	-3.6180111	0.00029688	0.02239124	Nhsl2	NHS-like 2
ENSMUSG0(	23.8482359	-8.1769786	2.56495927	-3.1879565	0.00143282	0.06801149	NA	NA
ENSMUSG0(	17.5448492	-7.7347978	2.3997563	-3.2231597	0.00126785	0.06342169	NA	NA
ENSMUSG0(	14.4724398	-20.873332	3.41360406	-6.1147491	9.67E-10	4.83E-07	NA	NA
ENSMUSG0(	19.5012492	-7.8865448	2.34124189	-3.3685305	0.0007557	0.04347265	NA	NA
ENSMUSG0(	18.9842093	-7.8490505	2.34659852	-3.344863	0.00082323	0.04616976	Skint6	selection and upkeep of intraepithelial T cells 6
ENSMUSG0(	21.7572936	-8.0454278	2.63882932	-3.0488625	0.0022971	0.09282613	Gm16537	predicted gene 16537
ENSMUSG0(	178.417263	-5.0449358	0.98633591	-5.1148253	3.14E-07	8.10E-05	Abhd12b	abhydrolase domain containing 12B
ENSMUSG0(	14.4724398	-20.873332	3.41360406	-6.1147491	9.67E-10	4.83E-07	S100a7l2	S100 calcium binding protein A7 like 2
ENSMUSG0(	25.0585975	-8.2483065	2.52145755	-3.2712454	0.00107075	0.05660696	NA	NA
ENSMUSG0(	6657.55752	-0.4095012	0.13020981	-3.1449333	0.00166125	0.07421195	Itpripl2	inositol 1,4,5-triphosphate receptor interacting protein-like 2
ENSMUSG0(	41.8308425	-8.05087	2.14218023	-3.7582599	0.0001711	0.01465387	NA	NA
ENSMUSG0(	391.544727	-1.5924186	0.46453522	-3.4279825	0.00060808	0.03762094	Kcnj11	potassium inwardly rectifying channel, subfamily J, member 11
ENSMUSG0(	17.6025885	-7.7396696	2.43917511	-3.1730685	0.00150837	0.07050785	Prickle4	prickle planar cell polarity protein 4
ENSMUSG0(	13117.1034	-0.6678028	0.12773369	-5.2280867	1.71E-07	4.62E-05	Kctd12	potassium channel tetramerisation domain containing 12
ENSMUSG0(	122.537666	-2.5561535	0.7912203	-3.230647	0.0012351	0.0621696	NA	NA
ENSMUSG0(	831.955475	-1.308688	0.40105785	-3.2630905	0.00110204	0.05755335	6030407O03	RIKEN cDNA 6030407O03 gene
ENSMUSG0(	26.2941473	-7.3767032	2.09184049	-3.5264176	0.00042122	0.02886063	Gm5049	glutamic pyruvic transaminase, soluble pseudogene
ENSMUSG0(	22.7376639	-8.1089808	2.18422466	-3.7125214	0.0002052	0.01685926	NA	NA
ENSMUSG0(	23.106431	-8.1312629	2.58232649	-3.1488129	0.00163935	0.07378046	Gm32200	predicted gene, 32200
ENSMUSG0(	19.7551178	-7.9054024	2.41974668	-3.2670372	0.00108679	0.05721372	NA	NA
ENSMUSG0(	24.6719884	-8.2263003	2.13114246	-3.8600424	0.00011337	0.01131357	NA	NA
ENSMUSG0(	20.6427202	-7.9700398	2.30647274	-3.4555101	0.00054925	0.03541291	NA	NA
ENSMUSG0(	23.474336	-8.1549753	2.59085116	-3.1476047	0.00164614	0.07394107	Mirlet7e	microRNA let7e
ENSMUSG0(	21.8108459	-8.0480122	2.64519645	-3.0425008	0.00234621	0.09381988	NA	NA
ENSMUSG0(	15.587274	-7.5638724	2.49944141	-3.0262251	0.00247628	0.09774125	NA	NA
ENSMUSG0(	15.9182967	-7.5943936	2.47721862	-3.0656938	0.00217166	0.08997478	NA	NA
ENSMUSG0(	37.3963034	-7.829689	1.81028794	-4.325107	1.52E-05	0.00236444	NA	NA
ENSMUSG0(	18.9317046	-7.8442006	2.34549771	-3.3443651	0.00082471	0.04616976	NA	NA
ENSMUSG0(	16.8232394	-7.6736875	2.53895193	-3.0223839	0.00250792	0.09823267	NA	NA
ENSMUSG0(	49.7707876	-9.2388507	1.72641168	-5.351476	8.72E-08	2.60E-05	4933406B17	RIKEN cDNA 4933406B17 gene

ENSMUSG0C	14.0589415	-20.83672	3.4454703	-6.0475691	1.47E-09	6.25E-07	NA	NA
ENSMUSG0C	14.885938	-20.917479	3.38316547	-6.1828128	6.30E-10	3.61E-07	NA	NA
ENSMUSG0C	32.3533807	-8.6166667	2.05992194	-4.1830064	2.88E-05	0.00383901	NA	NA
ENSMUSG0C	14.6791889	-20.894937	3.39821281	-6.1488017	7.81E-10	4.37E-07	NA	NA
ENSMUSG0C	22.9008569	-8.1188104	2.21582388	-3.6640143	0.00024829	0.01951735	NA	NA
ENSMUSG0C	14.2656906	-20.854088	3.42935205	-6.0810578	1.19E-09	5.40E-07	Gm5655	ubiquitin specific peptidase 1 pseudogene
ENSMUSG0C	17.0976108	-7.6975897	2.41324588	-3.1897246	0.00142408	0.06781538	A930002C04	RIKEN cDNA A930002C04 gene
ENSMUSG0C	29.4590466	-8.4816677	2.0999557	-4.0389746	5.37E-05	0.00625503	NA	NA
ENSMUSG0C	14.0589415	-20.83672	3.4454703	-6.0475691	1.47E-09	6.25E-07	Gm5209	predicted gene 5209
ENSMUSG0C	27.8961988	-8.4043993	2.42606634	-3.4642084	0.00053179	0.03457869	Gm35019	predicted gene, 35019
ENSMUSG0C	14.4724398	-20.873332	3.41360406	-6.1147491	9.67E-10	4.83E-07	NA	NA
ENSMUSG0C	25.9069877	-8.2964664	2.49092633	-3.3306751	0.00086636	0.04757292	NA	NA

Table 2. Upregulated in cKO cTEC

row	baseMean	log2FoldChai	lfcSE	stat	pvalue	padj	symbol	geneName
ENSMUSG0C	16763.9215	0.47996712	0.12606474	3.80730653	0.00014049	0.012766	Rpl13	ribosomal protein L13
ENSMUSG0C	10708.127	0.57934247	0.15126643	3.82994739	0.00012817	0.01205697	S100a6	S100 calcium binding protein A6 (calcyclin)
ENSMUSG0C	2342.94167	0.61831072	0.20163353	3.06650738	0.00216575	0.08989252	Mfsd10	major facilitator superfamily domain containing 10
ENSMUSG0C	4616.65878	5.15150395	0.48919833	10.530502	6.25E-26	4.78E-22	Slc13a2	solute carrier family 13 (sodium-dependent dicarboxylate transporter), member 2
ENSMUSG0C	6219.41497	0.43743756	0.12790369	3.42005415	0.00062609	0.03798041	Mcm3ap	minichromosome maintenance complex component 3 associated protein
ENSMUSG0C	429.831063	1.25423997	0.39534793	3.17249661	0.00151134	0.07050785	Rnd2	Rho family GTPase 2
ENSMUSG0C	4603.48337	0.54426267	0.13055028	4.16898893	3.06E-05	0.00403595	Ergic1	endoplasmic reticulum-golgi intermediate compartment (ERGIC) 1
ENSMUSG0C	2478.39246	0.89133276	0.20305445	4.38962432	1.14E-05	0.00183538	Gstt1	glutathione S-transferase, theta 1
ENSMUSG0C	8166.34642	0.48972388	0.1514626	3.23329904	0.00122369	0.06207366	Foxn1	forkhead box N1
ENSMUSG0C	6456.25127	0.5771446	0.15403374	3.74687122	0.00017905	0.01522158	Ndufa11	NADH:ubiquinone oxidoreductase subunit A11
ENSMUSG0C	481.917976	1.45310157	0.40198706	3.61479693	0.00030058	0.02254668	Gpx6	glutathione peroxidase 6
ENSMUSG0C	7711.36143	0.3651991	0.1119511	3.26213041	0.00110578	0.05755335	Grap	GRB2-related adaptor protein
ENSMUSG0C	4092.76439	0.44429736	0.1445937	3.07272965	0.00212111	0.08889137	Rsl1d1	ribosomal L1 domain containing 1
ENSMUSG0C	2199.08716	0.59080836	0.1738536	3.39830968	0.00067804	0.03990504	Cpt1c	carnitine palmitoyltransferase 1c
ENSMUSG0C	38971.5646	0.43822359	0.10143393	4.32028604	1.56E-05	0.00239348	Rplp1	ribosomal protein, large, P1
ENSMUSG0C	3218.84829	0.76802214	0.16784965	4.57565542	4.75E-06	0.00088536	Fbxw9	F-box and WD-40 domain protein 9
ENSMUSG0C	17873.4055	0.26975586	0.08609864	3.1331023	0.00172969	0.07679228	Ubc	ubiquitin C
ENSMUSG0C	22853.2705	0.38655803	0.10065204	3.84053865	0.00012276	0.01179003	Rps18	ribosomal protein S18
ENSMUSG0C	52.1349085	5.80130294	1.50699353	3.84958716	0.00011832	0.01167728	Gstt4	glutathione S-transferase, theta 4
ENSMUSG0C	8320.50122	0.46771654	0.10872255	4.30192753	1.69E-05	0.00250734	Rps25	ribosomal protein S25
ENSMUSG0C	2502.14832	0.60008774	0.19652661	3.05346816	0.00226213	0.09206136	Mrpl52	mitochondrial ribosomal protein L52
ENSMUSG0C	3892.78384	0.51848688	0.16454388	3.15105545	0.00162682	0.07336013	Ndufa2	NADH:ubiquinone oxidoreductase subunit A2
ENSMUSG0C	8019.18706	0.46440496	0.11392401	4.0764451	4.57E-05	0.00552436	Cox6c	cytochrome c oxidase subunit 6C
ENSMUSG0C	47849.7587	0.25494435	0.06676004	3.81881695	0.00013409	0.01231137	H3f3b	H3.3 histone B
ENSMUSG0C	15160.7344	0.44391828	0.09294887	4.77594046	1.79E-06	0.00037324	Rpl19	ribosomal protein L19
ENSMUSG0C	35080.5173	0.27008279	0.0880706	3.06666241	0.00216463	0.08989252	Igfbp4	insulin-like growth factor binding protein 4
ENSMUSG0C	6969.26759	0.58180587	0.15997459	3.63686432	0.00027598	0.02118564	Dalrd3	DALR anticodon binding domain containing 3
ENSMUSG0C	13216.7278	0.53944884	0.09969831	5.41081219	6.27E-08	1.95E-05	Ccng1	cyclin G1
ENSMUSG0C	14914.7273	0.40246245	0.09263382	4.34465989	1.39E-05	0.00220491	Rps27a	ribosomal protein S27A
ENSMUSG0C	2008.76237	0.721078	0.22147469	3.25580324	0.00113072	0.05819156	Mrps24	mitochondrial ribosomal protein S24
ENSMUSG0C	1078.737	1.02947531	0.31916438	3.22553327	0.00125738	0.0631525	Snhg16	small nucleolar RNA host gene 16
ENSMUSG0C	3179.64221	0.98536249	0.25649829	3.84159478	0.00012224	0.01179003	Nr1d1	nuclear receptor subfamily 1, group D, member 1
ENSMUSG0C	1349.65404	2.10520919	0.27375337	7.69016732	1.47E-14	2.59E-11	Krt24	keratin 24
ENSMUSG0C	2817.97923	0.58240404	0.17424911	3.3423644	0.00083068	0.04639071	Gstz1	glutathione transferase zeta 1 (maleylacetoacetate isomerase)
ENSMUSG0C	2042.52861	0.82554885	0.23438767	3.52215132	0.00042806	0.02915506	Dglucy	D-glutamate cyclase
ENSMUSG0C	1951.86277	0.67484465	0.21945546	3.07508705	0.00210441	0.08862853	Pcbd2	pterin 4 alpha carbinolamine dehydratase/dimerization cofactor of hepatocyte nuclear factor 1 alpha (TCF1) 2
ENSMUSG0C	5182.43667	0.54371286	0.16951941	3.20737819	0.00133951	0.06598771	Txndc15	thioredoxin domain containing 15
ENSMUSG0C	1497.99183	0.8522092	0.26617979	3.20163003	0.00136652	0.06645301	Thbs4	thrombospondin 4
ENSMUSG0C	30.8820778	8.25251411	2.03414305	4.0569979	4.97E-05	0.00586594	Dusp13	dual specificity phosphatase 13
ENSMUSG0C	6277.14667	0.53004148	0.16347648	3.24231028	0.00118565	0.06047598	Olfm4	olfactomedin 4
ENSMUSG0C	29.9861173	8.20980788	2.19884839	3.73368529	0.0001887	0.01586517	Rem2	rad and gem related GTP binding protein 2
ENSMUSG0C	3501.57015	0.46889932	0.15385796	3.04761163	0.00230668	0.09297382	Slc22a17	solute carrier family 22 (organic cation transporter), member 17
ENSMUSG0C	6626.66375	0.6124546	0.11978954	5.11275522	3.17E-07	8.10E-05	Carmil3	capping protein regulator and myosin 1 linker 3
ENSMUSG0C	3198.45416	0.52092729	0.15440837	3.37369852	0.00074166	0.04287964	Ntan1	N-terminal Asn amidase
ENSMUSG0C	13043.7233	0.74742521	0.15253426	4.90004825	9.58E-07	0.00021561	Sncg	synuclein, gamma
ENSMUSG0C	10701.2833	0.61198761	0.13898508	4.40326104	1.07E-05	0.00174829	Cdkn1a	cyclin-dependent kinase inhibitor 1A (P21)
ENSMUSG0C	50507.4726	0.39068736	0.1146224	3.40847311	0.00065328	0.03895464	Ccl25	chemokine (C-C motif) ligand 25
ENSMUSG0C	6123.62775	0.54132735	0.14523505	3.72725001	0.00019358	0.01604062	Nfkbie	nuclear factor of kappa light polypeptide gene enhancer in B cells inhibitor, epsilon
ENSMUSG0C	19270.1341	0.57922238	0.12083845	4.79336148	1.64E-06	0.00034857	Crip3	cysteine-rich protein 3
ENSMUSG0C	5280.65718	0.4099319	0.13582215	3.01815209	0.00254321	0.09893958	Clip4	CAP-GLY domain containing linker protein family, member 4
ENSMUSG0C	12204.9715	0.38874695	0.10509991	3.69883251	0.00021659	0.01762707	Hagh	hydroxyacyl glutathione hydrolase
ENSMUSG0C	3514.16857	0.50961751	0.14377572	3.54453105	0.00039331	0.02752346	Fibp	fibroblast growth factor (acidic) intracellular binding protein
ENSMUSG0C	734.444088	1.14631394	0.333887	3.43323919	0.00059642	0.03719981	Slit1	slit guidance ligand 1
ENSMUSG0C	18218.3575	0.39779624	0.10392321	3.82779021	0.0001293	0.01207393	Rps24	ribosomal protein S24
ENSMUSG0C	22389.0733	0.32962702	0.09332155	3.53216386	0.00041217	0.02841029	Rpl14	ribosomal protein L14
ENSMUSG0C	7811.4973	0.44493486	0.10542749	4.22029276	2.44E-05	0.00335341	Lancl1	LanC (bacterial lantibiotic synthetase component C)-like 1
ENSMUSG0C	852.974207	0.96670701	0.28080392	3.44264075	0.00057606	0.03652596	Plcd4	phospholipase C, delta 4
ENSMUSG0C	3163.33494	0.70092144	0.1854565	3.77943854	0.00015718	0.01382302	Smyd2	SET and MYND domain containing 2
ENSMUSG0C	3084.33958	0.58441884	0.15548848	3.75859901	0.00017087	0.01465387	Il2ra	interleukin 2 receptor, alpha chain
ENSMUSG0C	13725.3815	0.37646298	0.10091153	3.7306239	0.00019101	0.01594242	Notch1	notch 1
ENSMUSG0C	5271.09035	0.66988499	0.16841193	3.97765755	6.96E-05	0.00779254	Stk39	serine/threonine kinase 39
ENSMUSG0C	910.676992	4.35596089	0.50389448	8.64458949	5.40E-18	1.38E-14	Abcb11	ATP-binding cassette, sub-family B (MDR/TAP), member 11
ENSMUSG0C	2628.07368	0.5236763	0.1690691	3.09740989	0.0019522	0.08406902	Emc4	ER membrane protein complex subunit 4
ENSMUSG0C	3892.5662	0.61078963	0.15604278	3.91424481	9.07E-05	0.00959241	Pacsin3	protein kinase C and casein kinase substrate in neurons 3
ENSMUSG0C	12049.3446	0.31756611	0.10511676	3.02107966	0.00251875	0.09848874	Psma7	proteasome subunit alpha 7
ENSMUSG0C	170.189489	3.6683129	0.94259392	3.89172135	9.95E-05	0.01017437	1810062G17	RIKEN cDNA 1810062G17 gene
ENSMUSG0C	37.6515391	8.5403777	2.25840259	3.78160109	0.00015582	0.01382302	Veph1	ventricular zone expressed PH domain-containing 1
ENSMUSG0C	1220.20801	0.84218347	0.24446313	3.44503272	0.00057099	0.03630454	Pmf1	polyamine-modulated factor 1
ENSMUSG0C	26.2231871	8.01702197	2.09034661	3.83525962	0.00012543	0.0119462	Lrrc7	leucine rich repeat containing 7
ENSMUSG0C	14566.4022	0.38619423	0.09920919	3.89272651	9.91E-05	0.01017437	Rps20	ribosomal protein S20
ENSMUSG0C	24514.9978	0.33393632	0.09424259	3.54336937	0.00039505	0.02756097	Txn1	thioredoxin 1
ENSMUSG0C	3259.8594	0.65226111	0.18892233	3.45253577	0.00055534	0.03570534	Echdc2	enoyl Coenzyme A hydratase domain containing 2
ENSMUSG0C	4604.06991	0.66413564	0.19141884	3.46954175	0.00052135	0.03409253	Plb1	phospholipase B1
ENSMUSG0C	8379.45048	0.51214828	0.13799719	3.71129491	0.0002062	0.01685926	Ndufa4	Ndufa4, mitochondrial complex associated
ENSMUSG0C	295.78074	1.49990495	0.4730981	3.17038886	0.00152235	0.07073383	Timp4	tissue inhibitor of metalloproteinase 4
ENSMUSG0C	7199.58818	0.48612648	0.13325817	3.64800513	0.00026428	0.02049365	Cpxm2	carboxypeptidase X 2 (M14 family)
ENSMUSG0C	23.6839919	7.87076329	2.15994821	3.64395927	0.00026848	0.02074858	Iqcm	IQ motif containing M
ENSMUSG0C	30406.8469	0.30125587	0.08999471	3.34748431	0.00081549	0.04598981	Tagln	transgelin
ENSMUSG0C	4178.06756	1.47909086	0.20320581	7.27878226	3.37E-13	4.55E-10	Mcam	melanoma cell adhesion molecule
ENSMUSG0C	2641.34249	0.70110123	0.22968098	3.05250021	0.00226944	0.09219532	Stra6	stimulated by retinoic acid gene 6
ENSMUSG0C	5374.98656	0.44332899	0.12929021	3.42894484	0.00060593	0.03758911	Cox7a2	cytochrome c oxidase subunit 7A2
ENSMUSG0C	75515.2631	0.278675	0.0672527	4.14370002	3.42E-05	0.0044068	Rpl4	ribosomal protein L4
ENSMUSG0C	4227.57976	0.48206032	0.14557158	3.31150017	0.00092797	0.05023523	Mras	muscle and microspikes RAS
ENSMUSG0C	38.4983692	8.5717036	1.89605643	4.52080617	6.16E-06	0.0011047	Kbtbd12	kelch repeat and BTB (POZ) domain containing 12
ENSMUSG0C	8932.29165	0.37521717	0.11704412	3.20577556	0.00134699	0.06606295	Atp6v0b	ATPase, H+ transporting, lysosomal V0 subunit B
ENSMUSG0C	3660.13785	0.54392536	0.15223521	3.57292756	0.00035301	0.02564144	Eri3	exoribonuclease 3
ENSMUSG0C	2275.17093	0.61617528	0.19520532	3.15654962	0.00159648	0.07261604	Dph5	diphthamide biosynthesis 5
ENSMUSG0C	3307.4047	0.79220864	0.19631599	4.03537508	5.45E-05	0.00628787	Cpne2	copine II
ENSMUSG0C	192.308414	2.54436683	0.68941099	3.69063866	0.00022369	0.01795243	Celf5	CUGBP, Elav-like family member 5
ENSMUSG0C	2591.12149	0.79245154	0.18478506	4.28850444	1.80E-05	0.00263478	Ttc21b	tetratricopeptide repeat domain 21B
ENSMUSG0C	12965.0483	0.38493729	0.12075734	3.18769264	0.00143413	0.06801149	Rps29	ribosomal protein S29
ENSMUSG0C	4893.96826	0.94021861	0.17719119	5.30623788	1.12E-07	3.20E-05	Cobll1	Cobl-like 1
ENSMUSG0C	1767.20054	0.78231971	0.22738149	3.44056024	0.00058051	0.03660878	Mrpl54	mitochondrial ribosomal protein L54
ENSMUSG0C	11495.2833	0.66921653	0.10617579	6.3029108	2.92E-10	2.10E-07	Oma1	OMA1 zinc metallopeptidase
ENSMUSG0C	46129.0462	0.31213299	0.09576895	3.25922962	0.00111715	0.05777082	Ubd	ubiquitin D
ENSMUSG0C	1425.08361	0.78802602	0.2456949	3.20733563	0.00133971	0.06598771	Thyn1	thymocyte nuclear protein 1
ENSMUSG0C	17296.1743	0.36953256	0.10215316	3.61743646	0.00029754	0.02239124	Acta2	actin alpha 2, smooth muscle, aorta
ENSMUSG0C	7915.80602	0.46059971	0.13836688	3.32882934	0.00087212	0.04766126	Atp13a2	ATPase type 13A2
ENSMUSG0C	5239.69988	0.45668191	0.13341311	3.42306616	0.00061919	0.03798041	Micall2	MICAL-like 2
ENSMUSG0C	4558.94334	0.55373841	0.15090098	3.66954823	0.00024298	0.01923141	Rps27l	ribosomal protein S27-like
ENSMUSG0C	26.1545274	8.0152895	2.12044854	3.77999718	0.00015683	0.01382302	Prodh2	proline dehydrogenase (oxidase) 2
ENSMUSG0C	11107.8359	0.39474423	0.09780984	4.03583357	5.44E-05	0.00628787	Rps16	ribosomal protein S16
ENSMUSG0C	8466.69649	0.46194079	0.15205228	3.03803928	0.00238123	0.0948895	Inf2	inverted formin, FH2 and WH2 domain containing
ENSMUSG0C	7261.72516	0.47397864	0.12744298	3.71914275	0.0001999	0.0165047	Cd81	CD81 antigen
ENSMUSG0C	8947.98733	0.65095429	0.10674572	6.09817671	1.07E-09	5.13E-07	Rpl10a	ribosomal protein L10A
ENSMUSG0C	23.8690247	7.88023562	2.18875096	3.60033451	0.00031781	0.02368392	Gstp2	glutathione S-transferase, pi 2
ENSMUSG0C	11250.4855	0.43122281	0.12505061	3.44838615	0.00056395	0.0361572	Fau	Finkel-Biskis-Reilly murine sarcoma virus (FBR-MuSV) ubiquitously expressed (fox derived)
ENSMUSG0C	1158.20757	1.25888231	0.24021512	5.24064568	1.60E-07	4.37E-05	Afg1l	AFG1 like ATPase
ENSMUSG0C	10345.5624	0.46618367	0.11507014	4.05130001	5.09E-05	0.00596472	Ephx1	epoxide hydrolase 1, microsomal
ENSMUSG0C	26162.6342	0.38827168	0.0946259	4.10322856	4.07E-05	0.00505493	Rpl12	ribosomal protein L12
ENSMUSG0C	18769.0224	0.48205023	0.10684105	4.51184474	6.43E-06	0.00113469	Rps21	ribosomal protein S21
ENSMUSG0C	363.568147	1.52981113	0.48977482	3.12349895	0.00178715	0.07888527	Oxld1	oxidoreductase like domain containing 1
ENSMUSG0C	132.911807	2.68878248	0.73412484	3.66256844	0.0002497	0.01956089	Tspan10	tetraspanin 10
ENSMUSG0C	1174.08856	0.84384077	0.24569119	3.43455848	0.00059352	0.03712007	Dnm3	dynamin 3

ENSMUSG0(	5188.59031	0.5696592	0.14895473	3.82437794	0.0001311	0.01217643	Large2	LARGE xylosyl- and glucuronyltransferase 2
ENSMUSG0(	724.404253	1.29114066	0.33043136	3.90743979	9.33E-05	0.00977643	Matn1	matrilin 1, cartilage matrix protein
ENSMUSG0(	24.9587984	7.9455438	2.12902733	3.73200648	0.00018996	0.01591301	Tmco5b	transmembrane and coiled-coil domains 5B
ENSMUSG0(	18200.7993	0.30404446	0.08663645	3.50942899	0.00044907	0.03031618	Fmod	fibromodulin
ENSMUSG0(	7230.751	0.49560809	0.15884855	3.12000393	0.00180849	0.07931883	Tspo	translocator protein
ENSMUSG0(	12809.3512	0.4139075	0.11690611	3.54051203	0.00039935	0.02777671	Phlda3	pleckstrin homology like domain, family A, member 3
ENSMUSG0(	12315.2149	0.4410144	0.11103711	3.97177487	7.13E-05	0.00794877	Rpl37	ribosomal protein L37
ENSMUSG0(	4543.45708	0.56028488	0.16605346	3.37412348	0.00074051	0.04287964	Ndufa7	NADH:ubiquinone oxidoreductase subunit A7
ENSMUSG0(	926.147673	1.14257851	0.29362884	3.89123398	9.97E-05	0.01017437	Metap1d	methionyl aminopeptidase type 1D (mitochondrial)
ENSMUSG0(	13182.4348	0.46577979	0.12282295	3.79228622	0.00014927	0.01343576	S100a10	S100 calcium binding protein A10 (calpactin)
ENSMUSG0(	1416.67222	1.43902336	0.29467979	4.88334598	1.04E-06	0.00023019	Dusp15	dual specificity phosphatase-like 15
ENSMUSG0(	1307.84652	0.7940875	0.25094973	3.16432898	0.00155441	0.07178746	Sptssb	serine palmitoyltransferase, small subunit B
ENSMUSG0(	31761.0162	0.26799855	0.07846344	3.41558486	0.00063645	0.03834247	Rpl7	ribosomal protein L7
ENSMUSG0(	24.6352216	7.92578016	2.55569902	3.10121815	0.00192726	0.08315123	Stk-ps2	serine/threonine kinase 2
ENSMUSG0(	26.1864142	8.01538949	2.48022718	3.23171586	0.00123049	0.06207366	NA	NA
ENSMUSG0(	575.411437	0.98590596	0.32597481	3.02448511	0.00249057	0.09774125	D630023F18	RIKEN cDNA D630023F18 gene
ENSMUSG0(	9017.4721	0.76321204	0.15093096	5.05669639	4.27E-07	0.00010416	Col27a1	collagen, type XXVII, alpha 1
ENSMUSG0(	15975.164	0.58079012	0.12862174	4.5154894	6.32E-06	0.001124	Rpl37a	ribosomal protein L37a
ENSMUSG0(	7157.08935	0.46362034	0.11697349	3.96346504	7.39E-05	0.00819097	Rbp1	retinol binding protein 1, cellular
ENSMUSG0(	923.007052	1.0426525	0.34569162	3.0161347	0.00256019	0.09943173	Ltb4r1	leukotriene B4 receptor 1
ENSMUSG0(	25.5484539	7.97895254	2.12691016	3.75142903	0.00017583	0.01500303	Gjd3	gap junction protein, delta 3
ENSMUSG0(	29018.4878	0.37438381	0.0916038	4.08699016	4.37E-05	0.00531191	Rpl9	ribosomal protein L9
ENSMUSG0(	24100.7796	0.3578479	0.09598453	3.72818322	0.00019287	0.01603925	Rps8	ribosomal protein S8
ENSMUSG0(	6890.36851	0.54052146	0.12428745	4.34896231	1.37E-05	0.00218027	Foxo3	forkhead box O3
ENSMUSG0(	32.0127528	8.30497941	2.01266371	4.12636217	3.69E-05	0.00462254	Ifnb1	interferon beta 1, fibroblast
ENSMUSG0(	12194.8831	0.37571882	0.106946	3.51316395	0.0004428	0.02998137	Rps23	ribosomal protein S23
ENSMUSG0(	32.4193982	8.32447079	1.97019847	4.22519402	2.39E-05	0.00334127	Enthd1	ENTH domain containing 1
ENSMUSG0(	3157.85327	0.707768	0.1715116	4.12664806	3.68E-05	0.00462254	Commd3	COMM domain containing 3
ENSMUSG0(	42.1621314	4.74594994	1.4995131	3.16499398	0.00155086	0.071768	Tarm1	T cell-interacting, activating receptor on myeloid cells 1
ENSMUSG0(	10622.4756	0.4390571	0.12831667	3.42166834	0.00062238	0.03798041	Pdlim1	PDZ and LIM domain 1 (elfin)
ENSMUSG0(	16.259284	21.4543772	3.31568947	6.47056288	9.76E-11	7.23E-08	Ctsr	cathepsin R
ENSMUSG0(	20.1316099	7.6368348	2.27921538	3.35064201	0.00080624	0.04569316	Zfp981	zinc finger protein 981
ENSMUSG0(	6011.88064	0.46405299	0.13286845	3.49257475	0.00047839	0.0316439	Rpl38	ribosomal protein L38
ENSMUSG0(	15875.7895	0.48803947	0.1133928	4.30397229	1.68E-05	0.00250043	Rpl32	ribosomal protein L32
ENSMUSG0(	6958.0291	0.71500807	0.1537219	4.65130917	3.30E-06	0.00064707	Rpl36	ribosomal protein L36
ENSMUSG0(	26.1014191	8.01073405	2.08504533	3.84199515	0.00012204	0.01179003	Tdpoz3	TD and POZ domain containing 3
ENSMUSG0(	20050.4208	0.25575789	0.07992572	3.19994459	0.00137454	0.06670152	Rpl5	ribosomal protein L5
ENSMUSG0(	927.192263	1.23968983	0.36843905	3.36470803	0.00076625	0.04396917	Fcer1g	Fc receptor, IgE, high affinity I, gamma polypeptide
ENSMUSG0(	27888.6802	0.43610296	0.09534207	4.57408758	4.78E-06	0.00088536	Rpl11	ribosomal protein L11
ENSMUSG0(	4722.21831	0.4818419	0.15210319	3.16786184	0.00153564	0.07120738	Uqcr10	ubiquinol-cytochrome c reductase, complex III subunit X
ENSMUSG0(	1918.88959	0.60515819	0.19776475	3.05999013	0.00221344	0.09129006	Zfp637	zinc finger protein 637
ENSMUSG0(	20.9917887	7.69668785	2.3044162	3.33997298	0.00083787	0.04645296	Slc10a4-ps	solute carrier family 10 (sodium/bile acid cotransporter family), pseudogene
ENSMUSG0(	6199.60738	0.46610982	0.13092762	3.56005719	0.00037077	0.02651208	Rpl35a	ribosomal protein L35A
ENSMUSG0(	13383.5331	0.4446031	0.09960732	4.46355862	8.06E-06	0.00137699	Rpl26	ribosomal protein L26
ENSMUSG0(	13656.2895	0.42946035	0.10917601	3.93365119	8.37E-05	0.0090323	Rps7	ribosomal protein S7
ENSMUSG0(	29307.3024	0.37556332	0.09762674	3.84693101	0.00011961	0.01173218	Rps12	ribosomal protein S12
ENSMUSG0(	44.1540007	8.76963124	1.77573432	4.93859423	7.87E-07	0.00018244	Tex33	testis expressed 33
ENSMUSG0(	1459.31326	0.74937966	0.23090288	3.24543228	0.00117272	0.05999893	Ifnlr1	interferon lambda receptor 1
ENSMUSG0(	21149.8711	0.38811481	0.0829381	4.6795722	2.87E-06	0.00057377	Rpl17	ribosomal protein L17
ENSMUSG0(	5776.49319	0.57250374	0.14574172	3.92820772	8.56E-05	0.0091365	Rpl35	ribosomal protein L35
ENSMUSG0(	31539.3466	0.54689975	0.09195658	5.94736976	2.72E-09	1.06E-06	Rps15	ribosomal protein S15
ENSMUSG0(	17599.6903	0.63714276	0.0928769	6.86007746	6.88E-12	6.58E-09	Ifi27	interferon, alpha-inducible protein 27
ENSMUSG0(	6541.07529	0.6815243	0.16051566	4.24584294	2.18E-05	0.00310469	Scn1a	sodium channel, voltage-gated, type I, alpha
ENSMUSG0(	152829.91	0.35660604	0.11160287	3.19531248	0.0013968	0.06707247	ND1	NADH dehydrogenase subunit 1
ENSMUSG0(	113987.976	0.46091258	0.10057097	4.58295854	4.58E-06	0.00086251	ND2	NADH dehydrogenase subunit 2
ENSMUSG0(	462413.588	0.52319957	0.089859	5.82245013	5.80E-09	2.08E-06	COX1	cytochrome c oxidase subunit I
ENSMUSG0(	128665.229	0.58045148	0.09562813	6.06988206	1.28E-09	5.65E-07	ND4	NADH dehydrogenase subunit 4
ENSMUSG0(	139457.481	0.33994219	0.10475231	3.24519992	0.00117368	0.05999893	ND5	NADH dehydrogenase subunit 5
ENSMUSG0(	2401.4739	0.57451367	0.17973551	3.19643931	0.00139135	0.06707247	ND6	NADH dehydrogenase subunit 6
ENSMUSG0(	425078	0.52132624	0.09692408	5.37870734	7.50E-08	2.27E-05	CYTB	cytochrome b
ENSMUSG0(	1067.14228	0.84593833	0.26310822	3.2151725	0.00130366	0.06476828	Zfp810	zinc finger protein 810
ENSMUSG0(	9207.39345	0.3835609	0.11416192	3.3597971	0.00078	0.04464658	Rps28	ribosomal protein S28
ENSMUSG0(	20.430002	7.65776192	2.26476031	3.38126816	0.00072152	0.04203317	Krt74	keratin 74
ENSMUSG0(	11159.2849	0.53352564	0.14689895	3.63192269	0.00028132	0.02131049	Myl9	myosin, light polypeptide 9, regulatory
ENSMUSG0(	752.039159	1.28294605	0.37226074	3.44636414	0.00056818	0.03626985	Psrc1	proline/serine-rich coiled-coil 1
ENSMUSG0(	2559.994	0.75719731	0.23796732	3.18193825	0.00146293	0.06900439	Nr2f1	nuclear receptor subfamily 2, group F, member 1
ENSMUSG0(	3571.89677	0.49923	0.1619033	3.08350718	0.00204576	0.08695624	Psmb3	proteasome (prosome, macropain) subunit, beta type 3
ENSMUSG0(	24.6103532	6.95326302	2.2029557	3.15633356	0.00159766	0.07261604	Trcg1	taste receptor cell gene 1
ENSMUSG0(	100.878531	3.42760732	1.07256957	3.19569696	0.00139494	0.06707247	Hsf5	heat shock transcription factor family member 5
ENSMUSG0(	28.6025609	5.78893294	1.87785533	3.08273638	0.00205107	0.08702062	Npw	neuropeptide W
ENSMUSG0(	27.3289663	8.07582409	2.48455136	3.25041543	0.00115237	0.0591728	1700010D01	RIKEN cDNA 1700010D01 gene
ENSMUSG0(	32.8256996	8.34155989	1.9342836	4.31248027	1.61E-05	0.00243775	Mroh5	maestro heat-like repeat family member 5
ENSMUSG0(	3676.34011	0.50071741	0.16287992	3.07415064	0.00211103	0.08874437	Cops9	COP9 signalosome subunit 9
ENSMUSG0(	26.1730928	8.01498558	2.12976298	3.7633228	0.00016767	0.01457781	NA	NA
ENSMUSG0(	17224.9073	0.34515397	0.10845093	3.18258192	0.00145968	0.06900439	S100a16	S100 calcium binding protein A16
ENSMUSG0(	16499.8707	0.36623408	0.1158465	3.16137375	0.00157027	0.07226174	Nrbp2	nuclear receptor binding protein 2
ENSMUSG0(	985.181916	0.89501674	0.28791995	3.10856108	0.00188001	0.0812652	Fyb2	FYN binding protein 2
ENSMUSG0(	66.3446984	4.86325767	1.41351685	3.44053747	0.00058056	0.03660878	Serpina3m	serine (or cysteine) peptidase inhibitor, clade A, member 3M
ENSMUSG0(	839.648638	1.47541233	0.27618755	5.34206683	9.19E-08	2.70E-05	Col22a1	collagen, type XXII, alpha 1
ENSMUSG0(	6306.31975	0.55338624	0.15389806	3.59579731	0.0003234	0.02402262	Pglyrp2	peptidoglycan recognition protein 2
ENSMUSG0(	8100.55741	0.34610265	0.10877883	3.1817097	0.00146408	0.06900439	Rpl39	ribosomal protein L39
ENSMUSG0(	17.4052903	7.42784852	2.42219372	3.06657905	0.00216524	0.08989252	NA	NA
ENSMUSG0(	20.6661203	6.69815628	2.1924499	3.05510119	0.00224985	0.09172418	Gm15710	ribosomal protein L13 pseudogene
ENSMUSG0(	19.9427926	7.62426475	2.3155805	3.29259326	0.00099268	0.05311184	NA	NA
ENSMUSG0(	2839.7399	1.28502647	0.22606188	5.68440148	1.31E-08	4.43E-06	Slc13a2os	solute carrier family 13 (sodium-dependent dicarboxylate transporter), member 2, opposite strand
ENSMUSG0(	14.818588	21.3275925	3.41209478	6.2505862	4.09E-10	2.68E-07	NA	NA
ENSMUSG0(	95.5704953	4.57060797	1.28629819	3.55330359	0.00038043	0.02703374	Gm15462	predicted gene 15462
ENSMUSG0(	39.4261338	8.60475061	1.85374352	4.64182371	3.45E-06	0.00067176	Ubap1l	ubiquitin-associated protein 1-like
ENSMUSG0(	2190.46716	0.56044658	0.18186263	3.08170282	0.0020582	0.0871622	Snhg12	small nucleolar RNA host gene 12
ENSMUSG0(	32.4676844	5.56265793	1.78303015	3.11977783	0.00180987	0.07931883	NA	NA
ENSMUSG0(	1432.30935	0.86033645	0.24394522	3.52676085	0.00042068	0.02886063	Snhg20	small nucleolar RNA host gene 20
ENSMUSG0(	25.6239946	7.98268518	2.52656886	3.15949638	0.00158042	0.07226174	NA	NA
ENSMUSG0(	353.648338	1.32718619	0.43486569	3.05194506	0.00227364	0.09220281	NA	NA
ENSMUSG0(	25.2799254	7.96362759	2.52048323	3.15956381	0.00158005	0.07226174	NA	NA
ENSMUSG0(	38.7742493	5.49672761	1.65250492	3.3263003	0.00088007	0.04794185	NA	NA
ENSMUSG0(	53.0520073	9.03549648	1.73650253	5.20327286	1.96E-07	5.17E-05	Gm1604a	predicted gene 1604A
ENSMUSG0(	15977.056	0.37683493	0.11767095	3.20244667	0.00136266	0.06645301	Rps27	ribosomal protein S27
ENSMUSG0(	27819.535	0.40636876	0.08620135	4.71418084	2.43E-06	0.00049735	Myl6	myosin, light polypeptide 6, alkali, smooth muscle and non-muscle
ENSMUSG0(	19.5768692	7.59544292	2.40610555	3.1567372	0.00159545	0.07261604	Gm20611	predicted gene 20611
ENSMUSG0(	18640.0407	0.66404192	0.11128745	5.96690776	2.42E-09	9.74E-07	Rpl41	ribosomal protein L41
ENSMUSG0(	22.0429184	7.76689325	2.21675272	3.50372559	0.0004588	0.030702	Mir5623	microRNA 5623
ENSMUSG0(	3813.39745	0.51646996	0.14939428	3.45709329	0.00054604	0.03530467	Tomm20	translocase of outer mitochondrial membrane 20
ENSMUSG0(	1484.77136	1.2123473	0.26241465	4.61996815	3.84E-06	0.00073411	S100a2	S100 calcium binding protein A2
ENSMUSG0(	14.818588	21.3275925	3.41209478	6.2505862	4.09E-10	2.68E-07	NA	NA
ENSMUSG0(	21.037796	7.69915767	2.25657543	3.41187694	0.00064517	0.03866486	4833422M21	RIKEN cDNA 4833422M21 gene
ENSMUSG0(	24.8021982	7.93869509	2.27052841	3.49640861	0.00047157	0.03128282	NA	NA
ENSMUSG0(	24.1007526	7.89767554	2.19357554	3.60036635	0.00031777	0.02368392	Gm26644	predicted gene, 26644
ENSMUSG0(	738.521702	1.01733734	0.29925544	3.39956178	0.00067494	0.03990504	Snhg6	small nucleolar RNA host gene 6
ENSMUSG0(	590.457413	12.510217	3.90683557	3.20213554	0.00136413	0.06645301	Igkv19-93	immunoglobulin kappa chain variable 19-93
ENSMUSG0(	16.754462	7.37081544	2.43104467	3.03195393	0.00242976	0.09632184	Gm5575	heat shock protein 8 pseudogene
ENSMUSG0(	2109.46933	0.72461875	0.20510348	3.53294221	0.00041096	0.02841029	NA	NA
ENSMUSG0(	1144.56602	0.84297562	0.25314576	3.33000096	0.00086846	0.04757444	NA	NA
ENSMUSG0(	26.2066724	8.01512706	2.51091153	3.19211847	0.00141233	0.06752237	NA	NA
ENSMUSG0(	19.0295327	7.55758403	2.47162581	3.05773794	0.00223015	0.09132832	NA	NA
ENSMUSG0(	21.3847409	7.72136817	2.2717635	3.39884331	0.00067671	0.03990504	NA	NA
ENSMUSG0(	168.183156	1.91699467	0.63476686	3.01999801	0.00252776	0.09851805	NA	NA

ENSMUSG0C	20.2462493	7.64457156	2.47384333	3.09015994	0.00200049	0.08566639	NA	NA
ENSMUSG0C	19.4486195	7.58736879	2.36481724	3.2084377	0.00133458	0.06598771	NA	NA
ENSMUSG0C	18.4227575	7.50714056	2.37512298	3.16073763	0.0015737	0.07226174	2900060B14	RIKEN cDNA 2900060B14 gene
ENSMUSG0C	19.3525868	7.57938528	2.30764739	3.28446422	0.00102176	0.05428835	NA	NA
ENSMUSG0C	20.3953626	7.65684027	2.33198698	3.28339751	0.00102564	0.05436836	Lnp1	leukemia NUP98 fusion partner 1
ENSMUSG0C	58.5317524	3.95793083	1.27276969	3.10969915	0.00187278	0.0811055	NA	NA
ENSMUSG0C	23.7394398	7.87197651	2.20380065	3.57200027	0.00035427	0.02565125	NA	NA
ENSMUSG0C	33.2714988	8.35962245	2.01661991	4.14536345	3.39E-05	0.00439965	1600027J07F	RIKEN cDNA 1600027J07 gene
ENSMUSG0C	24.7662515	7.93754581	2.18532899	3.63219719	0.00028102	0.02131049	NA	NA
ENSMUSG0C	25.9622284	8.00247886	2.16750179	3.69202872	0.00022247	0.01791722	4933433G08	RIKEN cDNA 4933433G08 gene
ENSMUSG0C	23.2303073	7.84125026	2.20351519	3.55851881	0.00037295	0.026585	NA	NA
ENSMUSG0C	29.8755927	8.20508135	1.99534377	4.11211415	3.92E-05	0.00489064	Gm33340	predicted gene, 33340
ENSMUSG0C	1748.86654	0.8316157	0.23434425	3.54869265	0.00038715	0.02737734	NA	NA
ENSMUSG0C	4081.0768	0.61923793	0.19529486	3.17078461	0.00152028	0.07073383	NA	NA
ENSMUSG0C	23.1312746	7.83783633	2.55871379	3.06319384	0.00218988	0.0905664	NA	NA
ENSMUSG0C	4407.28923	0.77563455	0.22885829	3.38914771	0.0007011	0.04115704	NA	NA
ENSMUSG0C	4390.81662	0.91262081	0.2179131	4.18800335	2.81E-05	0.00378459	Bc1	brain cytoplasmic RNA 1
ENSMUSG0C	23.5255617	7.85961608	2.59206546	3.03218272	0.00242792	0.09632184	NA	NA

Table 3. Downregulated in cKO mTEC

row	baseMean	log2FoldChar	lfcSE	stat	pvalue	padj	symbol	geneName
ENSMUSG0C	1600.81614	-0.7070154	0.16941932	-4.1731688	3.00E-05	0.00230429	H19	H19, imprinted maternally expressed transcript
ENSMUSG0C	1311.07349	-0.3756973	0.11983613	-3.1350917	0.001718	0.04683633	Cdh4	cadherin 4
ENSMUSG0C	1421.29775	-0.4778123	0.13227496	-3.6122659	0.00030353	0.01413214	Gm2a	GM2 ganglioside activator protein
ENSMUSG0C	1421.76529	-0.4344665	0.14790294	-2.9375107	0.00330859	0.07219604	Sez6	seizure related gene 6
ENSMUSG0C	1299.4911	-0.4723119	0.13257868	-3.5625029	0.00036734	0.01611798	Ckb	creatine kinase, brain
ENSMUSG0C	1662.09463	-0.5607984	0.12771024	-4.3911783	1.13E-05	0.00108459	Peg3	paternally expressed 3
ENSMUSG0C	6673.47419	-0.3087839	0.10339214	-2.9865319	0.00282161	0.06586162	Jag2	jagged 2
ENSMUSG0C	186.134532	-0.7772669	0.2575738	-3.0176473	0.00254745	0.06056476	Prkar2b	protein kinase, cAMP dependent regulatory, type II beta
ENSMUSG0C	982.473998	-0.6953374	0.11641265	-5.97304	2.33E-09	6.54E-07	Hck	hemopoietic cell kinase
ENSMUSG0C	3029.08884	-0.3158784	0.09051964	-3.4896115	0.00048372	0.01972617	Pld3	phospholipase D family, member 3
ENSMUSG0C	529.912357	-0.5717633	0.16827541	-3.3977829	0.00067934	0.02506229	St8sia6	ST8 alpha-N-acetyl-neuraminide alpha-2,8-sialyltransferase 6
ENSMUSG0C	1145.07205	-0.3144156	0.10080283	-3.1191149	0.00181395	0.04877782	Coq8b	coenzyme Q8B
ENSMUSG0C	101.45256	-1.0041516	0.30930772	-3.2464484	0.00116855	0.03685519	Hlf	hepatic leukemia factor
ENSMUSG0C	5313.13126	-0.2829519	0.08460933	-3.3442165	0.00082515	0.02902705	Ptpn6	protein tyrosine phosphatase, non-receptor type 6
ENSMUSG0C	867.60374	-0.5505607	0.12109286	-4.5465993	5.45E-06	0.00059334	Gab2	growth factor receptor bound protein 2-associated protein 2
ENSMUSG0C	357.029227	-1.2780237	0.20554551	-6.2177164	5.04E-10	1.63E-07	Chn2	chimerin 2
ENSMUSG0C	1135.26156	-0.5260657	0.14091782	-3.7331384	0.00018911	0.00974007	Pgf	placental growth factor
ENSMUSG0C	412.433402	-0.4762462	0.16411017	-2.901991	0.00370799	0.07854898	Sh2b2	SH2B adaptor protein 2
ENSMUSG0C	1306.01053	-0.3802054	0.12204321	-3.1153344	0.00183737	0.04907061	Klf5	Kruppel-like factor 5
ENSMUSG0C	10304.1181	-0.2773428	0.09923188	-2.7948959	0.00519164	0.09861739	Cic	capicua transcriptional repressor
ENSMUSG0C	5353.94366	-0.3897009	0.08823452	-4.4166487	1.00E-05	0.00098618	Igf1r	insulin-like growth factor I receptor
ENSMUSG0C	5620.6393	-0.6375779	0.12132878	-5.2549602	1.48E-07	2.53E-05	Cyp2a5	cytochrome P450, family 2, subfamily a, polypeptide 5
ENSMUSG0C	1705.60819	-0.6558202	0.10547273	-6.2179128	5.04E-10	1.63E-07	Atp4a	ATPase, H+/K+ exchanging, gastric, alpha polypeptide
ENSMUSG0C	5008.57636	-0.2566496	0.08162128	-3.1443956	0.0016643	0.04629473	Kit	KIT proto-oncogene receptor tyrosine kinase
ENSMUSG0C	937.679799	-0.828628	0.14177103	-5.8448331	5.07E-09	1.28E-06	Neurl1a	neuralized E3 ubiquitin protein ligase 1A
ENSMUSG0C	1984.58561	-0.5397713	0.16532294	-3.2649511	0.00109483	0.03570088	Krt23	keratin 23
ENSMUSG0C	592.634183	-0.4950679	0.17469749	-2.8338584	0.00459897	0.0905755	Chrd	chordin
ENSMUSG0C	6462.12969	-0.4446486	0.11373492	-3.9095168	9.25E-05	0.00551497	Atp1a2	ATPase, Na+/K+ transporting, alpha 2 polypeptide
ENSMUSG0C	2507.52286	-0.4656794	0.11681621	-3.986428	6.71E-05	0.00442419	Id3	inhibitor of DNA binding 3
ENSMUSG0C	584.872068	-0.5354345	0.1743598	-3.0708598	0.00213443	0.05382006	Fzd3	frizzled class receptor 3
ENSMUSG0C	1340.44817	-0.3470099	0.12056065	-2.8783018	0.00399822	0.08270165	Rbfox1	RNA binding protein, fox-1 homolog (C. elegans) 1
ENSMUSG0C	4009.01518	-1.1443795	0.14318959	-7.9920581	1.33E-15	1.44E-12	Trpm5	transient receptor potential cation channel, subfamily M, member 5
ENSMUSG0C	781.274962	-0.4228057	0.12963498	-3.2615092	0.00110821	0.03586846	Otog	otogelin
ENSMUSG0C	1855.4424	-0.3005035	0.1009197	-2.9776496	0.00290468	0.06690366	Kcnq1	potassium voltage-gated channel, subfamily Q, member 1
ENSMUSG0C	679.057807	-0.484393	0.15269505	-3.1722898	0.00151242	0.04338676	Pou6f1	POU domain, class 6, transcription factor 1
ENSMUSG0C	2145.122	-0.3514804	0.09969934	-3.5254031	0.00042284	0.0179975	Cacna2d2	calcium channel, voltage-dependent, alpha 2/delta subunit 2
ENSMUSG0C	579.932084	-0.5302707	0.16595548	-3.1952585	0.00139706	0.04116234	Spire2	spire type actin nucleation factor 2
ENSMUSG0C	459.241816	-0.8069683	0.1720583	-4.6900864	2.73E-06	0.00033488	Prox1	prospero homeobox 1
ENSMUSG0C	599.981113	-0.4967501	0.16809944	-2.955097	0.00312571	0.0694234	Iglon5	IgLON family member 5
ENSMUSG0C	572.658032	-0.390348	0.13427836	-2.907006	0.00364906	0.07758379	Clip3	CAP-GLY domain containing linker protein 3
ENSMUSG0C	4797.32191	-0.2568188	0.08049758	-3.1903909	0.0014208	0.04159741	Arnt2	aryl hydrocarbon receptor nuclear translocator 2
ENSMUSG0C	1469.01247	-0.3294746	0.10497568	-3.1385806	0.00169768	0.0467749	Eps8	epidermal growth factor receptor pathway substrate 8
ENSMUSG0C	604.005976	-0.5832885	0.13895899	-4.1975585	2.70E-05	0.0021068	Il17rb	interleukin 17 receptor B
ENSMUSG0C	2121.66728	-0.3300881	0.11229659	-2.939431	0.00328815	0.07193045	Chdh	choline dehydrogenase
ENSMUSG0C	286.325416	-0.6519379	0.18882536	-3.4525973	0.00055522	0.02182392	Syt14	synaptotagmin XIV
ENSMUSG0C	684.972643	-0.59736	0.15555167	-3.8402673	0.0001229	0.0069407	Sertad4	SERTA domain containing 4
ENSMUSG0C	625.191802	-0.5959008	0.17034103	-3.4982811	0.00046827	0.01934153	Eef1a2	eukaryotic translation elongation factor 1 alpha 2
ENSMUSG0C	248.048904	-0.7829588	0.21564021	-3.6308571	0.00028248	0.0134763	Il10	interleukin 10
ENSMUSG0C	8252.12673	-0.280491	0.07854849	-3.5709283	0.00035572	0.01591028	Cntnap1	contactin associated protein-like 1
ENSMUSG0C	2203.62046	-0.2465192	0.08767849	-2.8116272	0.00492916	0.09473669	Nlk	nemo like kinase
ENSMUSG0C	443.847512	-0.5547547	0.17933257	-3.0934409	0.0019785	0.05139883	Hnf4a	hepatic nuclear factor 4, alpha
ENSMUSG0C	2884.55921	-0.3337303	0.10643434	-3.135551	0.00171532	0.04683633	Cadps2	Ca2+-dependent activator protein for secretion 2
ENSMUSG0C	2087.32142	-0.3578406	0.12458379	-2.8722887	0.00407511	0.08376493	Zbtb4	zinc finger and BTB domain containing 4
ENSMUSG0C	2355.77959	-0.3398754	0.10487154	-3.2408739	0.00119164	0.03725315	Acsl1	acyl-CoA synthetase long-chain family member 1
ENSMUSG0C	1569.10332	-0.3284107	0.1116477	-2.9414913	0.00326636	0.07163367	Syne4	spectrin repeat containing, nuclear envelope family member 4
ENSMUSG0C	16202.1203	-0.2259079	0.06939196	-3.2555341	0.00113179	0.03636146	Cd164	CD164 antigen
ENSMUSG0C	197.225407	-0.7273731	0.20650993	-3.5222187	0.00042795	0.01813117	Mgat4c	MGAT4 family, member C
ENSMUSG0C	3286.8058	-0.3344127	0.11345409	-2.9475594	0.00320293	0.07077749	Arid5b	AT rich interactive domain 5B (MRF1-like)
ENSMUSG0C	2684.8151	-0.4407416	0.13227633	-3.3319759	0.00086232	0.03004725	Sgk1	serum/glucocorticoid regulated kinase 1
ENSMUSG0C	1384.65856	-0.3784221	0.12297068	-3.0773359	0.0020886	0.05301569	Unc5b	unc-5 netrin receptor B
ENSMUSG0C	935.204363	-0.4684963	0.12390909	-3.7809683	0.00015622	0.00838512	Cpm	carboxypeptidase M
ENSMUSG0C	541.85196	-0.5108671	0.17933778	-2.8486308	0.00439078	0.08796053	Ltc4s	leukotriene C4 synthase
ENSMUSG0C	954.630063	-0.5431683	0.18185417	-2.9868344	0.00281882	0.06586162	Aebp1	AE binding protein 1
ENSMUSG0C	781.460658	-1.1484627	0.1955001	-5.8744868	4.24E-09	1.12E-06	Pik3cg	phosphatidylinositol-4,5-bisphosphate 3-kinase catalytic subunit gamma
ENSMUSG0C	678.19052	-0.4594351	0.16126393	-2.8489638	0.00438619	0.08796053	Rgs9	regulator of G-protein signaling 9
ENSMUSG0C	202.842952	-0.9642039	0.21163707	-4.5559314	5.22E-06	0.00057117	Fam20a	FAM20A, golgi associated secretory pathway pseudokinase
ENSMUSG0C	2234.09472	-0.457044	0.10769123	-4.2440224	2.20E-05	0.0018033	Id2	inhibitor of DNA binding 2
ENSMUSG0C	240.042507	-0.5847811	0.18432351	-3.1725802	0.00151091	0.04338676	Mboat2	membrane bound O-acyltransferase domain containing 2
ENSMUSG0C	213.890063	-0.6532786	0.18976926	-3.4424891	0.00057639	0.02235329	Rab37	RAB37, member RAS oncogene family
ENSMUSG0C	14700.0997	-0.40516	0.09412574	-4.304455	1.67E-05	0.00144677	Atp2a3	ATPase, Ca++ transporting, ubiquitous
ENSMUSG0C	4538.54688	-0.3576553	0.10338781	-3.4593562	0.00054147	0.02142861	Sec14l1	SEC14-like lipid binding 1
ENSMUSG0C	279.806336	-0.7333214	0.25520909	-2.8734142	0.00406061	0.08357856	Doc2b	double C2, beta
ENSMUSG0C	2604.36212	-0.8383857	0.10890326	-7.6984439	1.38E-14	1.33E-11	Pik3r5	phosphoinositide-3-kinase regulatory subunit 5
ENSMUSG0C	1984.76973	-0.3254739	0.1029435	-3.1616749	0.00156865	0.04461724	Sptb	spectrin beta, erythrocytic
ENSMUSG0C	3250.75548	-0.4007767	0.13117892	-3.0551912	0.00224917	0.05594973	Clmn	calmin
ENSMUSG0C	104.616641	-1.3274573	0.37244263	-3.5641926	0.00036498	0.01611798	Meg3	maternally expressed 3
ENSMUSG0C	1523.87113	-0.493688	0.15435624	-3.1983675	0.00138208	0.04099858	Id4	inhibitor of DNA binding 4
ENSMUSG0C	502.788952	-0.7902594	0.14112119	-5.5998631	2.15E-08	4.50E-06	Barx1	BarH-like homeobox 1
ENSMUSG0C	1642.99016	-1.3246875	0.12646574	-10.474674	1.13E-25	3.93E-22	Cxcl14	chemokine (C-X-C motif) ligand 14
ENSMUSG0C	1535.78503	-0.5073561	0.16814309	-3.017407	0.00254947	0.06056476	Adcy2	adenylate cyclase 2
ENSMUSG0C	2327.16637	-0.9689638	0.13580362	-7.1350367	9.68E-13	6.02E-10	Mctp1	multiple C2 domains, transmembrane 1
ENSMUSG0C	111.533057	-0.8938733	0.31564554	-2.8318897	0.00462738	0.09094424	Acot12	acyl-CoA thioesterase 12
ENSMUSG0C	1496.96057	-0.3395689	0.11981545	-2.8340993	0.00459551	0.0905755	Iqgap2	IQ motif containing GTPase activating protein 2
ENSMUSG0C	183.184468	-0.7094307	0.24122704	-2.9409251	0.00327234	0.07167447	Rgs7bp	regulator of G-protein signalling 7 binding protein
ENSMUSG0C	3419.73652	-0.3640684	0.09754124	-3.732456	0.00018962	0.00974007	Emb	embigin
ENSMUSG0C	1359.70188	-0.5049154	0.17301526	-2.9183289	0.00351913	0.07555931	Fst	follistatin
ENSMUSG0C	1407.48926	-0.3465582	0.11712488	-2.9588775	0.00308762	0.06896803	Txndc16	thioredoxin domain containing 16
ENSMUSG0C	332.867037	-0.9811583	0.21173367	-4.6339269	3.59E-06	0.00041931	Chat	choline acetyltransferase
ENSMUSG0C	1813.86455	-0.3993428	0.12431905	-3.2122411	0.00131704	0.03974624	Nefm	neurofilament, medium polypeptide
ENSMUSG0C	990.86245	-0.5187006	0.15259106	-3.3992855	0.00067562	0.02497792	Nefl	neurofilament, light polypeptide
ENSMUSG0C	1914.5618	-0.6082739	0.10201827	-5.9624013	2.49E-09	6.87E-07	Fyb	FYN binding protein
ENSMUSG0C	500.074412	-0.6189653	0.17422225	-3.5527341	0.00038125	0.01662242	Sema5a	sema domain, seven thrombospondin repeats (type 1 and type 1-like), transmembrane domain (TM) and short cytoplasmic domain, (semaphorin) 5A
ENSMUSG0C	409.895029	-0.6053806	0.20556436	-2.9449686	0.00322987	0.07110214	Adcy8	adenylate cyclase 8
ENSMUSG0C	271.741746	-0.773279	0.20751127	-3.7264434	0.0001942	0.00988915	Syngr1	synaptogyrin 1
ENSMUSG0C	1710.44001	-0.3782672	0.11369799	-3.3269473	0.00087803	0.03039589	Deptor	DEP domain containing MTOR-interacting protein
ENSMUSG0C	2371.37665	-0.4793986	0.13942772	-3.4383303	0.00058531	0.02258073	Col2a1	collagen, type II, alpha 1
ENSMUSG0C	1376.57456	-0.4904806	0.15637711	-3.1365246	0.00170963	0.04683633	Psca	prostate stem cell antigen
ENSMUSG0C	8146.97905	-0.3446646	0.0770888	-4.4710078	7.79E-06	0.00079743	Alcam	activated leukocyte cell adhesion molecule
ENSMUSG0C	520.976905	-0.4937765	0.1518107	-3.2525805	0.00114362	0.03667383	Aifm3	apoptosis-inducing factor, mitochondrion-associated 3
ENSMUSG0C	355.335271	-1.0069204	0.20592523	-4.8897378	1.01E-06	0.00014179	Clic6	chloride intracellular channel 6
ENSMUSG0C	1294.32761	-0.5365963	0.15423308	-3.4791256	0.00050305	0.02032403	Runx1	runt related transcription factor 1
ENSMUSG0C	19598.5565	-0.3038633	0.07917936	-3.8376576	0.00012421	0.00697719	Ivns1abp	influenza virus NS1A binding protein
ENSMUSG0C	409.604395	-0.7449667	0.2060587	-3.6153131	0.00029998	0.01404203	Kcnk5	potassium channel, subfamily K, member 5
ENSMUSG0C	3044.52381	-0.9861361	0.15341595	-6.4278589	1.29E-10	5.12E-08	Gnb3	guanine nucleotide binding protein (G protein), beta 3
ENSMUSG0C	1253.08445	-0.4489482	0.14862114	-3.0207563	0.00252144	0.06026117	Thbs2	thrombospondin 2
ENSMUSG0C	6042.38251	-0.2977615	0.09783493	-3.0435092	0.00233836	0.05737458	Lbh	limb-bud and heart
ENSMUSG0C	1028.6178	-0.7655876	0.12727984	-6.014995	1.80E-09	5.22E-07	Nrxn1	neurexin I
ENSMUSG0C	300.43358	-0.7433326	0.23159864	-3.2095724	0.00132933	0.03990957	Cidea	cell death-inducing DNA fragmentation factor, alpha subunit-like effector A
ENSMUSG0C	955.084072	-0.528853	0.12634871	-4.1856621	2.84E-05	0.00219961	Setbp1	SET binding protein 1
ENSMUSG0C	8589.29091	-0.2384749	0.07670969	-3.1087981	0.0018785	0.04983062	Pip5k1b	phosphatidylinositol-4-phosphate 5-kinase, type 1 beta
ENSMUSG0C	181.10086	-0.7203608	0.23261282	-3.0968235	0.00195606	0.05106586	Cnnm1	cyclin M1
ENSMUSG0C	5842.32125	-0.9986143	0.13464346	-7.4167305	1.20E-13	1.05E-10	Avil	advillin
ENSMUSG0C	636.261793	-0.7299535	0.21378122	-3.4144884	0.00063902	0.02414943	Prap1	proline-rich acidic protein 1
ENSMUSG0C	3674.53071	-0.2793108	0.08343187	-3.3477707	0.00081464	0.02880715	Ric8a	RIC8 guanine nucleotide exchange factor A
ENSMUSG0C	2566.64152	-0.4599385	0.13838828	-3.3235369	0.00088884	0.03058758	Cdhr5	cadherin-related family member 5
ENSMUSG0C	5693.55011	-0.3924586	0.08896075	-4.4115925	1.03E-05	0.00100382	Eps8l2	EPS8-like 2
ENSMUSG0C	624.450633	-0.4110036	0.14723025	-2.7915702	0.0052453	0.09891409	Tmem80	transmembrane protein 80
ENSMUSG0C	236.641427	-0.6370661	0.20920764	-3.045138	0.00232574	0.05720062	Pde8a	phosphodiesterase 8A
ENSMUSG0C	1857.01309	-0.6357842	0.14655076	-4.3383206	1.44E-05	0.00130511	Alox5	arachidonate 5-lipoxygenase
ENSMUSG0C	319.834368	-0.6534999	0.22024381	-2.9671659	0.00300559	0.06774365	Fbxl16	F-box and leucine-rich repeat protein 16
ENSMUSG0C	650.192695	-0.5848536	0.18102909	-3.2307163	0.0012348	0.03798878	Gng13	guanine nucleotide binding protein (G protein), gamma 13
ENSMUSG0C	2744.88141	-0.4233408	0.10394016	-4.072928	4.64E-05	0.00324663	Cplx2	complexin 2
ENSMUSG0C	4833.75839	-0.4820143	0.11626128	-4.1459576	3.38E-05	0.00253987	Tfap2b	transcription factor AP-2 beta
ENSMUSG0C	1575.028	-0.3132494	0.09273794	-3.3777914	0.0007307	0.02652754	Klf7	Kruppel-like factor 7 (ubiquitous)
ENSMUSG0C	8719.86936	-0.3716991	0.1067269	-3.4827127	0.00049636	0.02014715	Il18r1	interleukin 18 receptor 1
ENSMUSG0C	1133.01434	-0.4275431	0.11997254	-3.5636745	0.0003657	0.01611798	Cracdl	capping protein inhibiting regulator of actin like
ENSMUSG0C	1245.11988	-0.5408002	0.14058453	-3.8467972	0.00011967	0.00680996	Ptpn18	protein tyrosine phosphatase, non-receptor type 18
ENSMUSG0C	1035.87686	-0.3399128	0.11619168	-2.9254492	0.00343959	0.07440203	Col9a1	collagen, type IX, alpha 1
ENSMUSG0C	75.8678426	-1.1461637	0.39427381	-2.9070246	0.00364884	0.07758379	Tmem169	transmembrane protein 169
ENSMUSG0C	1864.81197	-0.559666	0.166358	-3.3642263	0.00076759	0.027502	Lypd1	Ly6/Plaur domain containing 1
ENSMUSG0C	212.397163	-0.6435566	0.2064804	-3.1167926	0.0018283	0.04897879	Tmem163	transmembrane protein 163
ENSMUSG0C	1494.45001	-0.3493213	0.10889701	-3.2078137	0.00133748	0.0400853	Rgs2	regulator of G-protein signaling 2
ENSMUSG0C	2673.27037	-0.5209219	0.09437562	-5.5196661	3.40E-08	6.88E-06	Tfcp2l1	transcription factor CP2-like 1

ENSMUSG0C	6840.71832	-0.414484	0.08706424	-4.7606687	1.93E-06	0.00024525	Nfasc	neurofascin
ENSMUSG0C	840.104365	-0.8542541	0.18939917	-4.5103371	6.47E-06	0.00068306	Myog	myogenin
ENSMUSG0C	314.326853	-0.6445543	0.20410873	-3.1578966	0.00158912	0.04486155	Dusp27	dual specificity phosphatase 27 (putative)
ENSMUSG0C	13774.456	-0.3181488	0.11102808	-2.8654807	0.00416377	0.08499844	Atp1b1	ATPase, Na+/K+ transporting, beta 1 polypeptide
ENSMUSG0C	875.469967	-0.452439	0.12403365	-3.6477119	0.00026459	0.01283353	Itga8	integrin alpha 8
ENSMUSG0C	482.843262	-0.8279997	0.26149961	-3.1663516	0.00154364	0.04406462	Eng	endoglin
ENSMUSG0C	385.658433	-0.5527055	0.1752423	-3.15395	0.00161077	0.04538557	Galnt5	polypeptide N-acetylgalactosaminyltransferase 5
ENSMUSG0C	2458.9062	-0.5844573	0.11900007	-4.9114029	9.04E-07	0.00012802	Neb	nebulin
ENSMUSG0C	724.892696	-0.4593618	0.16191029	-2.8371379	0.00455199	0.09007259	Slc43a1	solute carrier family 43, member 1
ENSMUSG0C	909.658965	-0.8020044	0.18634003	-4.3039836	1.68E-05	0.00144677	Gfra4	glial cell line derived neurotrophic factor family receptor alpha 4
ENSMUSG0C	2904.43608	-0.3589951	0.11109601	-3.2313951	0.00123188	0.03796574	Pdyn	prodynorphin
ENSMUSG0C	1402.31948	-0.4189405	0.14055563	-2.980603	0.00287681	0.06650074	Tgm3	transglutaminase 3, E polypeptide
ENSMUSG0C	1608.37076	-1.1629933	0.26797704	-4.3398993	1.43E-05	0.00130511	Pck1	phosphoenolpyruvate carboxykinase 1, cytosolic
ENSMUSG0C	720.405213	-0.5747341	0.146792	-3.9152959	9.03E-05	0.00542165	Car2	carbonic anhydrase 2
ENSMUSG0C	1133.95087	-0.4701356	0.14366192	-3.2725136	0.00106596	0.03503542	Epb41l1	erythrocyte membrane protein band 4.1 like 1
ENSMUSG0C	106.594833	-0.9862643	0.31464767	-3.1345038	0.00172145	0.04683633	Olfml3	olfactomedin-like 3
ENSMUSG0C	989.857851	-0.3148723	0.11112294	-2.8335495	0.00460342	0.0905755	Tspan2	tetraspanin 2
ENSMUSG0C	186.900552	-0.7909453	0.27384602	-2.8882848	0.00387349	0.08097129	Kcnc4	potassium voltage gated channel, Shaw-related subfamily, member 4
ENSMUSG0C	1321.7688	-0.5594418	0.15169773	-3.6878718	0.00022614	0.01118674	Enpep	glutamyl aminopeptidase
ENSMUSG0C	460.573108	-0.7113789	0.20862424	-3.4098573	0.00064997	0.02441999	Dkk2	dickkopf WNT signaling pathway inhibitor 2
ENSMUSG0C	724.070466	-0.4838255	0.12802283	-3.7792129	0.00015732	0.00838512	Calb1	calbindin 1
ENSMUSG0C	1057.19767	-0.3447751	0.11683636	-2.9509231	0.00316826	0.07012041	Brinp1	bone morphogenic protein/retinoic acid inducible neural specific 1
ENSMUSG0C	852.814518	-0.3884955	0.1306647	-2.9732247	0.00294689	0.06715766	Fmn2	formin 2
ENSMUSG0C	752.098695	-0.5084801	0.17108461	-2.9720973	0.00295773	0.06715766	Kif12	kinesin family member 12
ENSMUSG0C	1046.22349	-0.4652612	0.15403887	-3.0204141	0.00252429	0.06026117	Zfp618	zinc finger protein 618
ENSMUSG0C	2918.08009	-0.3343191	0.11526297	-2.9004904	0.00372579	0.07861012	Snx30	sorting nexin family member 30
ENSMUSG0C	3717.88345	-0.2980677	0.08591824	-3.4692018	0.00052201	0.02080025	Dyrk2	dual-specificity tyrosine-(Y)-phosphorylation regulated kinase 2
ENSMUSG0C	374.957201	-0.8960192	0.20859954	-4.2954035	1.74E-05	0.00148985	Kcnq4	potassium voltage-gated channel, subfamily Q, member 4
ENSMUSG0C	1743.44845	-0.4894777	0.12608125	-3.8822397	0.0001035	0.00608859	Tspan1	tetraspanin 1
ENSMUSG0C	2716.17223	-0.3620117	0.09596407	-3.7723669	0.00016171	0.00850692	Pink1	PTEN induced putative kinase 1
ENSMUSG0C	2020.30973	-1.2028438	0.14010715	-8.5851707	9.07E-18	1.44E-14	Gnat3	guanine nucleotide binding protein, alpha transducing 3
ENSMUSG0C	132.481305	-0.6830782	0.24417596	-2.7974833	0.00515024	0.09816838	A3galt2	alpha 1,3-galactosyltransferase 2 (isoglobotriaosylceramide synthase)
ENSMUSG0C	3028.208	-0.5575102	0.11199394	-4.9780393	6.42E-07	9.48E-05	Espn	espin
ENSMUSG0C	322.743249	-0.6674489	0.21186938	-3.1502847	0.00163111	0.04559307	Tas1r3	taste receptor, type 1, member 3
ENSMUSG0C	6234.54723	-0.2467008	0.07557534	-3.2643036	0.00109734	0.0357157	Sel1l3	sel-1 suppressor of lin-12-like 3 (C. elegans)
ENSMUSG0C	3081.28511	-0.2450754	0.08321498	-2.9450875	0.00322863	0.07110214	D5Ertd579e	DNA segment, Chr 5, ERATO Doi 579, expressed
ENSMUSG0C	232.314972	-0.6536832	0.21721836	-3.0093367	0.00261819	0.06186769	Cckar	cholecystokinin A receptor
ENSMUSG0C	258.537409	-0.6191238	0.21345884	-2.9004364	0.00372643	0.07861012	Chrna9	cholinergic receptor, nicotinic, alpha polypeptide 9
ENSMUSG0C	228.549037	-0.6513995	0.22615042	-2.8803818	0.00397194	0.08243547	Sult1d1	sulfotransferase family 1D, member 1
ENSMUSG0C	976.882864	-0.6016083	0.16953651	-3.5485472	0.00038736	0.01677897	Nos1	nitric oxide synthase 1, neuronal
ENSMUSG0C	787.934804	-0.5835311	0.17087782	-3.4149026	0.00063805	0.02414943	Rimbp2	RIMS binding protein 2
ENSMUSG0C	200.59491	-0.8485617	0.24614313	-3.4474319	0.00056594	0.02209589	Fezf1	Fez family zinc finger 1
ENSMUSG0C	18846.3274	-0.3005307	0.07482547	-4.0164223	5.91E-05	0.00395733	Ahcyl2	S-adenosylhomocysteine hydrolase-like 2
ENSMUSG0C	1092.01505	-0.5500731	0.11431712	-4.8118173	1.50E-06	0.00020034	Hpgds	hematopoietic prostaglandin D synthase
ENSMUSG0C	17953.9328	-0.4718966	0.13042801	-3.618062	0.00029682	0.01400672	Reg3g	regenerating islet-derived 3 gamma
ENSMUSG0C	1036.41105	-0.4205385	0.13530705	-3.1080308	0.00188338	0.049841	Gxylt2	glucoside xylosyltransferase 2
ENSMUSG0C	4278.19278	-0.9612208	0.13647532	-7.0431835	1.88E-12	1.13E-09	Irag2	inositol 1,4,5-triphosphate receptor associated 2
ENSMUSG0C	1190.10997	-0.5370854	0.15194252	-3.5347933	0.00040809	0.01745982	Tmtc1	transmembrane and tetratricopeptide repeat containing 1
ENSMUSG0C	1908.17234	-0.3929252	0.11885513	-3.3059166	0.00094666	0.03200823	Slc8a2	solute carrier family 8 (sodium/calcium exchanger), member 2
ENSMUSG0C	503.673023	-0.543312	0.17337562	-3.1337277	0.00172601	0.04683633	Ckm	creatine kinase, muscle
ENSMUSG0C	3288.9634	-0.4268368	0.09302835	-4.5882443	4.47E-06	0.00050542	Psd3	pleckstrin and Sec7 domain containing 3
ENSMUSG0C	4714.18966	-0.267634	0.09003245	-2.9726394	0.00295251	0.06715766	Kctd15	potassium channel tetramerisation domain containing 15
ENSMUSG0C	767.516216	-0.5498077	0.15041582	-3.6552515	0.00025693	0.01249699	Pcsk6	proprotein convertase subtilisin/kexin type 6
ENSMUSG0C	4435.53601	-0.3435204	0.10702131	-3.2098314	0.00132813	0.03990957	Tjp1	tight junction protein 1
ENSMUSG0C	1601.39355	-0.3379646	0.10928015	-3.0926436	0.00198382	0.05140521	Crtc3	CREB regulated transcription coactivator 3
ENSMUSG0C	6951.65352	-0.3188484	0.09375016	-3.4010438	0.00067129	0.02487061	Furin	furin (paired basic amino acid cleaving enzyme)
ENSMUSG0C	5235.56519	-0.3363068	0.08209553	-4.0965306	4.19E-05	0.00299657	Ctsc	cathepsin C
ENSMUSG0C	591.664306	-0.4439468	0.1469822	-3.0204123	0.00252431	0.06026117	Sytl2	synaptotagmin-like 2
ENSMUSG0C	569.392933	-0.4133718	0.13654438	-3.0273808	0.00246683	0.05957685	Tmc5	transmembrane channel-like gene family 5
ENSMUSG0C	354.756562	-0.6534675	0.17483787	-3.7375626	0.00018581	0.00960106	Pde3b	phosphodiesterase 3B, cGMP-inhibited
ENSMUSG0C	710.921858	-0.4758607	0.15069328	-3.1578097	0.00158959	0.04486155	Mylpf	myosin light chain, phosphorylatable, fast skeletal muscle
ENSMUSG0C	2725.39177	-0.3855246	0.09745969	-3.9557337	7.63E-05	0.00484896	Sez6l2	seizure related 6 homolog like 2
ENSMUSG0C	492.647769	-0.699924	0.17721973	-3.94947	7.83E-05	0.0049595	Pgm2l1	phosphoglucomutase 2-like 1
ENSMUSG0C	727.559795	-0.5893171	0.18641628	-3.1612962	0.00157069	0.04461724	Slco2b1	solute carrier organic anion transporter family, member 2b1
ENSMUSG0C	3721.06938	-0.4095178	0.14337814	-2.8562083	0.00428734	0.08670779	Myh14	myosin, heavy polypeptide 14
ENSMUSG0C	2025.48721	-0.3726141	0.10453956	-3.5643359	0.00036478	0.01611798	Arhgap17	Rho GTPase activating protein 17
ENSMUSG0C	1790.91281	-0.4162913	0.11232031	-3.7062868	0.00021032	0.0105847	Pak1	p21 (RAC1) activated kinase 1
ENSMUSG0C	706.295756	-0.5722905	0.17256118	-3.3164498	0.00091169	0.0311891	Ptpn5	protein tyrosine phosphatase, non-receptor type 5
ENSMUSG0C	1045.06471	-0.3966424	0.12434997	-3.1897268	0.00142407	0.04159741	Cdr2	cerebellar degeneration-related 2
ENSMUSG0C	461.881156	-0.4223412	0.1492882	-2.8290324	0.0046689	0.09155349	Hpx	hemopexin
ENSMUSG0C	930.395539	-0.4433563	0.153213	-2.8937248	0.00380702	0.07996572	Crym	crystallin, mu
ENSMUSG0C	1529.29104	-0.3854668	0.12711068	-3.0325289	0.00242514	0.05881468	Anks4b	ankyrin repeat and sterile alpha motif domain containing 4B
ENSMUSG0C	1634.30961	-0.3610562	0.12922225	-2.7940714	0.0052049	0.09861739	Stk33	serine/threonine kinase 33
ENSMUSG0C	367.15463	-1.1691364	0.27116414	-4.311545	1.62E-05	0.00143297	Mrgprf	MAS-related GPR, member F
ENSMUSG0C	235.630184	-0.7206488	0.2216993	-3.2505688	0.00115174	0.03668296	Srpx2	sushi-repeat-containing protein, X-linked 2
ENSMUSG0C	234.269213	-1.4358762	0.27344954	-5.2509731	1.51E-07	2.56E-05	Dcx	doublecortin
ENSMUSG0C	1080.72618	-1.2384206	0.17357044	-7.1349744	9.68E-13	6.02E-10	Bmx	BMX non-receptor tyrosine kinase
ENSMUSG0C	1326.41919	-0.3513082	0.11583648	-3.0327942	0.00242301	0.05881468	Abcd1	ATP-binding cassette, sub-family D (ALD), member 1
ENSMUSG0C	1561.77335	-0.6115124	0.11510985	-5.312425	1.08E-07	1.94E-05	Arhgap4	Rho GTPase activating protein 4
ENSMUSG0C	1646.11793	-0.3933412	0.1233156	-3.1897113	0.00142415	0.04159741	L1cam	L1 cell adhesion molecule
ENSMUSG0C	477.928005	-0.5691447	0.20058471	-2.8374282	0.00454786	0.09007259	F10	coagulation factor X
ENSMUSG0C	575.69206	-0.8825771	0.16880694	-5.2283221	1.71E-07	2.84E-05	Slit2	slit guidance ligand 2
ENSMUSG0C	4943.96767	-0.5043447	0.13620308	-3.7028878	0.00021316	0.01069666	Hpgd	hydroxyprostaglandin dehydrogenase 15 (NAD)
ENSMUSG0C	326.929979	-0.5554292	0.19936515	-2.7859895	0.00533646	0.09959676	Nr3c2	nuclear receptor subfamily 3, group C, member 2
ENSMUSG0C	626.578022	-0.5231	0.17627432	-2.9675335	0.003002	0.06774365	Sorbs2	sorbin and SH3 domain containing 2
ENSMUSG0C	989.815126	-0.5132131	0.13124095	-3.9104646	9.21E-05	0.00551225	Crispld2	cysteine-rich secretory protein LCCL domain containing 2
ENSMUSG0C	342.904353	-0.9737226	0.21547063	-4.5190502	6.21E-06	0.00065955	Cdh13	cadherin 13
ENSMUSG0C	378.612398	-0.8585462	0.18412817	-4.662764	3.12E-06	0.00036957	Car7	carbonic anhydrase 7
ENSMUSG0C	452.324455	-1.1134197	0.25387393	-4.3857188	1.16E-05	0.0011	Acta1	actin alpha 1, skeletal muscle
ENSMUSG0C	756.507792	-0.7430285	0.13908582	-5.3422307	9.18E-08	1.70E-05	Agt	angiotensinogen (serpin peptidase inhibitor, clade A, member 8)
ENSMUSG0C	301.415086	-0.5120504	0.17959654	-2.8511154	0.00435662	0.08770145	Usp2	ubiquitin specific peptidase 2
ENSMUSG0C	2106.75787	-0.4201942	0.10622336	-3.9557612	7.63E-05	0.00484896	Pou2f3	POU domain, class 2, transcription factor 3
ENSMUSG0C	742.019951	-0.4428429	0.13629548	-3.2491388	0.00115755	0.03672836	Kirrel3	kirre like nephrin family adhesion molecule 3
ENSMUSG0C	780.495795	-0.5915278	0.18402294	-3.2144245	0.00130706	0.03967499	Lipc	lipase, hepatic
ENSMUSG0C	2713.68747	-0.5572513	0.13237274	-4.2097131	2.56E-05	0.00203307	Ltf	lactotransferrin
ENSMUSG0C	547.61057	-0.7737251	0.13206352	-5.8587348	4.66E-09	1.21E-06	Rab6b	RAB6B, member RAS oncogene family
ENSMUSG0C	2132.39609	-0.2868106	0.09435351	-3.0397452	0.00236778	0.05798906	Prkar2a	protein kinase, cAMP dependent regulatory, type II alpha
ENSMUSG0C	501.725525	-0.8735156	0.17481072	-4.9969221	5.83E-07	8.82E-05	Lmo2	LIM domain only 2
ENSMUSG0C	5095.84101	-0.2482777	0.07493761	-3.3131255	0.0009226	0.03143867	Inppl1	inositol polyphosphate phosphatase-like 1
ENSMUSG0C	1556.41035	-0.3752827	0.11829451	-3.172444	0.00151162	0.04338676	Tpcn1	two pore channel 1
ENSMUSG0C	2039.70868	-0.3384757	0.10158865	-3.3318259	0.00086278	0.03004725	Heyl	hairy/enhancer-of-split related with YRPW motif-like
ENSMUSG0C	130.31879	-1.0952105	0.31856526	-3.4379472	0.00058614	0.02258073	P2ry2	purinergic receptor P2Y, G-protein coupled 2
ENSMUSG0C	1897.33824	-0.3358132	0.09086042	-3.695924	0.00021909	0.01089997	Nfatc1	nuclear factor of activated T cells, cytoplasmic, calcineurin dependent 1
ENSMUSG0C	2325.69787	-0.4287373	0.13609561	-3.1502653	0.00163122	0.04559307	Cdo1	cysteine dioxygenase 1, cytosolic
ENSMUSG0C	3264.69442	-0.3222962	0.09464008	-3.4054938	0.00066045	0.02467882	Lmo7	LIM domain only 7
ENSMUSG0C	2198.64527	-0.5273377	0.15962454	-3.3036128	0.00095448	0.03211497	Resp18	regulated endocrine-specific protein 18
ENSMUSG0C	1399.5036	-0.3928094	0.11213256	-3.5030803	0.00045991	0.01915892	Lpcat2	lysophosphatidylcholine acyltransferase 2
ENSMUSG0C	742.543231	-0.5280692	0.14536117	-3.6328076	0.00028035	0.01341155	Shf	Src homology 2 domain containing F
ENSMUSG0C	492.95726	-0.5837246	0.16720055	-3.4911646	0.00048092	0.0197035	Strc	stereocilin
ENSMUSG0C	195.978915	-0.8174419	0.26508472	-3.0837007	0.00204443	0.05250692	Fa2h	fatty acid 2-hydroxylase
ENSMUSG0C	292.603675	-0.7497305	0.19521203	-3.8405957	0.00012274	0.0069407	Adra2a	adrenergic receptor, alpha 2a
ENSMUSG0C	113.432159	-1.1498422	0.32849	-3.5003873	0.00046458	0.01930735	Emx1	empty spiracles homeobox 1
ENSMUSG0C	87.5515319	-0.9163769	0.30812664	-2.9740269	0.00293919	0.06715766	Fndc3c1	fibronectin type III domain containing 3C1
ENSMUSG0C	407.546733	-0.6950519	0.22401383	-3.1027187	0.00191752	0.05043767	St18	suppression of tumorigenicity 18
ENSMUSG0C	188.98458	-0.6095883	0.21842126	-2.7908833	0.00525644	0.09891409	Zswim5	zinc finger SWIM-type containing 5
ENSMUSG0C	933.883906	-0.8996978	0.14202288	-6.3348791	2.38E-10	8.27E-08	Ano7	anoctamin 7
ENSMUSG0C	146.145375	-0.8113197	0.24035304	-3.3755332	0.00073673	0.02667078	Scn11a	sodium channel, voltage-gated, type XI, alpha
ENSMUSG0C	1612.6752	-0.843345	0.14703319	-5.7357459	9.71E-09	2.32E-06	Vav1	vav 1 oncogene
ENSMUSG0C	849.097233	-0.7665532	0.12723211	-6.0248402	1.69E-09	5.08E-07	Scx	scleraxis
ENSMUSG0C	2935.80701	-0.3920353	0.12572259	-3.118257	0.00181924	0.04881117	Myo15b	myosin XVB
ENSMUSG0C	612.485478	-1.0075026	0.15037624	-6.6998792	2.09E-11	9.56E-09	Lrrn1	leucine rich repeat protein 1, neuronal
ENSMUSG0C	224.433188	-0.7772832	0.22669943	-3.428695	0.00060649	0.02331308	Fam171a2	family with sequence similarity 171, member A2
ENSMUSG0C	1613.80393	-0.3351765	0.12011547	-2.7904525	0.00526344	0.09891409	Adgrb1	adhesion G protein-coupled receptor B1
ENSMUSG0C	677.460584	-0.5188546	0.15737598	-3.2969112	0.00097754	0.03261922	Gpsm3	G-protein signalling modulator 3 (AGS3-like, C. elegans)
ENSMUSG0C	1220.62041	-0.5798096	0.14233108	-4.0736681	4.63E-05	0.00324663	Cdhr2	cadherin-related family member 2
ENSMUSG0C	2322.72485	-0.3056034	0.10035488	-3.0452269	0.00232505	0.05720062	Cog7	component of oligomeric golgi complex 7
ENSMUSG0C	6578.72985	-0.3437473	0.09444204	-3.6397698	0.00027288	0.01315231	Cebpa	CCAAT/enhancer binding protein (C/EBP), alpha
ENSMUSG0C	2307.98441	-0.5324279	0.1111435	-4.790455	1.66E-06	0.00021989	Vat1	vesicle amine transport 1
ENSMUSG0C	1638.41308	-0.3818072	0.11558163	-3.3033553	0.00095535	0.03211497	Eef2k	eukaryotic elongation factor-2 kinase
ENSMUSG0C	309.974276	-1.0367556	0.20085549	-5.161699	2.45E-07	3.98E-05	Pdzrn3	PDZ domain containing RING finger 3
ENSMUSG0C	2279.68681	-0.4511987	0.10733575	-4.2036198	2.63E-05	0.0020697	Ints6l	integrator complex subunit 6 like
ENSMUSG0C	570.889915	-0.9334376	0.14920962	-6.2558809	3.95E-10	1.32E-07	1810046K07I	RIKEN cDNA 1810046K07 gene
ENSMUSG0C	252.751112	-0.5784317	0.19602311	-2.9508341	0.00316917	0.07012041	Ccdc38	coiled-coil domain containing 38
ENSMUSG0C	1895.65986	-0.6163228	0.10665355	-5.7787367	7.53E-09	1.85E-06	Cracd	capping protein inhibiting regulator of actin
ENSMUSG0C	490.1479	-0.6494838	0.15090659	-4.3038795	1.68E-05	0.00144677	Ppfibp2	PTPRF interacting protein, binding protein 2 (liprin beta 2)

ENSMUSG0C	353.050462	-0.7043212	0.20881584	-3.3729301	0.00074373	0.02686834	Sh3tc1	SH3 domain and tetratricopeptide repeats 1
ENSMUSG0C	392.463221	-0.8262849	0.18870908	-4.3786177	1.19E-05	0.00113028	Mag	myelin-associated glycoprotein
ENSMUSG0C	247.181247	-0.6745657	0.21908795	-3.078972	0.00207716	0.05294725	Dchs1	dachsous cadherin related 1
ENSMUSG0C	632.1005	-0.7281447	0.23042042	-3.1600702	0.00157731	0.04473245	C1qa	complement component 1, q subcomponent, alpha polypeptide
ENSMUSG0C	1118.39219	-0.4811012	0.17227172	-2.7926881	0.00522721	0.09882883	C1qb	complement component 1, q subcomponent, beta polypeptide
ENSMUSG0C	778.825676	-0.5809374	0.18140024	-3.2025173	0.00136232	0.04060441	Tns2	tensin 2
ENSMUSG0C	1157.27311	-0.4728455	0.11543371	-4.0962509	4.20E-05	0.00299657	Apbb1	amyloid beta (A4) precursor protein-binding, family B, member 1
ENSMUSG0C	5361.32169	-0.6357571	0.12271872	-5.1806036	2.21E-07	3.63E-05	Clca3b	chloride channel accessory 3B
ENSMUSG0C	5628.39923	-0.2874463	0.08993789	-3.1960537	0.00139321	0.04111863	Pax1	paired box 1
ENSMUSG0C	108.559627	-1.3098046	0.33942342	-3.8589105	0.00011389	0.00656699	Tas2r108	taste receptor, type 2, member 108
ENSMUSG0C	388.94735	-0.8066243	0.18595163	-4.3378177	1.44E-05	0.00130511	Wee2	WEE1 homolog 2 (S. pombe)
ENSMUSG0C	3282.36634	-0.2838065	0.10049068	-2.8242068	0.00473978	0.09243113	Dennd11	DENN domain containing 11
ENSMUSG0C	573.394319	-0.5622421	0.17312498	-3.2476081	0.00116379	0.03684573	Man1c1	mannosidase, alpha, class 1C, member 1
ENSMUSG0C	1064.19559	-0.5264945	0.12511379	-4.2081257	2.57E-05	0.00203809	Ccn3	cellular communication network factor 3
ENSMUSG0C	929.458619	-0.55979	0.15998645	-3.498984	0.00046703	0.01934153	Shank2	SH3 and multiple ankyrin repeat domains 2
ENSMUSG0C	404.263221	-0.7242177	0.1678347	-4.3150657	1.60E-05	0.00141752	Adgrl3	adhesion G protein-coupled receptor L3
ENSMUSG0C	5849.94211	-1.1020279	0.11227746	-9.8152191	9.68E-23	1.87E-19	Cdkn1c	cyclin-dependent kinase inhibitor 1C (P57)
ENSMUSG0C	1170.08976	-0.8764223	0.15952087	-5.494092	3.93E-08	7.86E-06	D630003M21	RIKEN cDNA D630003M21 gene
ENSMUSG0C	1813.31514	-0.3043156	0.09441839	-3.2230544	0.00126831	0.03881399	Ano10	anoctamin 10
ENSMUSG0C	459.903562	-0.5447915	0.17525628	-3.1085417	0.00188013	0.04983062	Card19	caspase recruitment domain family, member 19
ENSMUSG0C	239.705797	-0.8979068	0.2969058	-3.0242144	0.0024928	0.05980575	Itprid1	ITPR interacting domain containing 1
ENSMUSG0C	2750.3134	-0.6452146	0.15087115	-4.276594	1.90E-05	0.0015964	Muc5ac	mucin 5, subtypes A and C, tracheobronchial/gastric
ENSMUSG0C	345.30867	-0.4719604	0.15724948	-3.001348	0.00268787	0.06316318	Mturn	maturin, neural progenitor differentiation regulator homolog (Xenopus)
ENSMUSG0C	1555.28829	-0.3874145	0.13001793	-2.9797009	0.0028853	0.06654533	Aox4	aldehyde oxidase 4
ENSMUSG0C	579.723899	-0.6793985	0.13263041	-5.1224943	3.02E-07	4.82E-05	Kcnh2	potassium voltage-gated channel, subfamily H (eag-related), member 2
ENSMUSG0C	516.588961	-0.4870306	0.15468402	-3.1485516	0.00164082	0.0457145	Satb2	special AT-rich sequence binding protein 2
ENSMUSG0C	6283.70584	-0.295634	0.0915302	-3.229907	0.0012383	0.03802928	Sbf2	SET binding factor 2
ENSMUSG0C	83.8432174	-1.0582866	0.33931267	-3.1189127	0.0018152	0.04877782	Hjv	hemojuvelin BMP co-receptor
ENSMUSG0C	160.395382	-0.7170468	0.23694748	-3.0261846	0.00247661	0.05964761	Grtp1	GH regulated TBC protein 1
ENSMUSG0C	5000.65839	-0.502155	0.14162844	-3.5455806	0.00039175	0.01692687	Rgs4	regulator of G-protein signaling 4
ENSMUSG0C	384.28006	-0.7254124	0.190034	-3.8172767	0.00013493	0.00748275	Pln	phospholamban
ENSMUSG0C	755.593547	-0.3959632	0.13561407	-2.9197798	0.00350279	0.07530129	Lrrc56	leucine rich repeat containing 56
ENSMUSG0C	1378.16853	-0.5441832	0.12387426	-4.3930288	1.12E-05	0.00108137	Fsd2	fibronectin type III and SPRY domain containing 2
ENSMUSG0C	423.817502	-0.7279436	0.19291364	-3.7734171	0.00016103	0.00850692	Dgki	diacylglycerol kinase, iota
ENSMUSG0C	323.601475	-0.7917265	0.19314055	-4.0992248	4.15E-05	0.00298278	Shank1	SH3 and multiple ankyrin repeat domains 1
ENSMUSG0C	2028.05707	-0.2689674	0.09515253	-2.826697	0.00470308	0.0919133	Mvb12b	multivesicular body subunit 12B
ENSMUSG0C	809.437898	-0.4672005	0.13767564	-3.393487	0.00069009	0.02535128	Sema6c	sema domain, transmembrane domain (TM), and cytoplasmic domain, (semaphorin) 6C
ENSMUSG0C	344.447632	-0.7299523	0.2044416	-3.5704684	0.00035634	0.01591028	Lefty1	left right determination factor 1
ENSMUSG0C	2107.24812	-0.4265751	0.10854967	-3.9297689	8.50E-05	0.00523175	Rap1gap2	RAP1 GTPase activating protein 2
ENSMUSG0C	892.531474	-0.5224985	0.16155842	-3.2341151	0.0012202	0.03780674	Slco4a1	solute carrier organic anion transporter family, member 4a1
ENSMUSG0C	565.412865	-0.9641071	0.24119447	-3.9972188	6.41E-05	0.00427593	Klk1b16	kallikrein 1-related peptidase b16
ENSMUSG0C	1516.81374	-0.3258968	0.10292088	-3.1664794	0.00154296	0.04406462	Cables2	CDK5 and Abl enzyme substrate 2
ENSMUSG0C	853.21125	-0.5876311	0.15650015	-3.7548276	0.00017346	0.00903242	Zfp503	zinc finger protein 503
ENSMUSG0C	12707.561	-0.2864176	0.08845904	-3.2378553	0.00120432	0.03744786	Abhd2	abhydrolase domain containing 2
ENSMUSG0C	567.904734	-0.4960553	0.16551959	-2.9969584	0.00272688	0.06390733	Ttc36	tetratricopeptide repeat domain 36
ENSMUSG0C	537.552456	-0.8846937	0.16490857	-5.3647526	8.11E-08	1.52E-05	Ajap1	adherens junction associated protein 1
ENSMUSG0C	2210.24878	-0.2775301	0.09713182	-2.857252	0.00427326	0.0866244	Lonrf1	LON peptidase N-terminal domain and ring finger 1
ENSMUSG0C	1419.06836	-0.4694451	0.12318526	-3.8108868	0.00013847	0.00763026	Plekhg5	pleckstrin homology domain containing, family G (with RhoGef domain) member 5
ENSMUSG0C	2364.70017	-0.5737821	0.10640082	-5.3926473	6.94E-08	1.34E-05	Cited2	Cbp/p300-interacting transactivator, with Glu/Asp-rich carboxy-terminal domain, 2
ENSMUSG0C	790.050209	-0.6806004	0.14122064	-4.8194115	1.44E-06	0.00019435	Hip1	huntingtin interacting protein 1
ENSMUSG0C	3183.04073	-0.9711356	0.23489723	-4.1342997	3.56E-05	0.00264944	Plcb2	phospholipase C, beta 2
ENSMUSG0C	1560.87789	-0.5591766	0.11362454	-4.921266	8.60E-07	0.00012273	Cacna2d1	calcium channel, voltage-dependent, alpha2/delta subunit 1
ENSMUSG0C	1393.42256	-0.3897684	0.13849183	-2.8143785	0.00488717	0.09434613	Rgl3	ral guanine nucleotide dissociation stimulator-like 3
ENSMUSG0C	480.004472	-1.0068242	0.17909732	-5.6216596	1.89E-08	4.07E-06	Hmx3	H6 homeobox 3
ENSMUSG0C	1690.38659	-0.5327262	0.13455568	-3.9591511	7.52E-05	0.00484114	Fmo2	flavin containing monooxygenase 2
ENSMUSG0C	2584.14871	-0.256126	0.09087743	-2.8183675	0.00482685	0.09380578	Nsmce4a	NSE4 homolog A, SMC5-SMC6 complex component
ENSMUSG0C	9546.99694	-0.3623391	0.08694023	-4.1676802	3.08E-05	0.00235011	Dgkz	diacylglycerol kinase zeta
ENSMUSG0C	507.444229	-0.612057	0.19716334	-3.1043144	0.00190721	0.05031843	Cyp2s1	cytochrome P450, family 2, subfamily s, polypeptide 1
ENSMUSG0C	1078.82885	-0.5261359	0.13642693	-3.8565399	0.000115	0.00660909	Zfhx2	zinc finger homeobox 2
ENSMUSG0C	518.714291	-0.457849	0.15907256	-2.8782397	0.00399901	0.08270165	Dlk1	delta like non-canonical Notch ligand 1
ENSMUSG0C	100.631699	-1.0300539	0.32805584	-3.1398736	0.00169021	0.04664276	Foxa3	forkhead box A3
ENSMUSG0C	715.723794	-0.6466841	0.15248784	-4.2408893	2.23E-05	0.00182007	Atp1a3	ATPase, Na+/K+ transporting, alpha 3 polypeptide
ENSMUSG0C	311.484884	-0.8359266	0.2132174	-3.9205366	8.84E-05	0.00537928	Arhgef38	Rho guanine nucleotide exchange factor (GEF) 38
ENSMUSG0C	1199.21902	-0.3448143	0.12218486	-2.8220707	0.00477147	0.092833	Fam117b	family with sequence similarity 117, member B
ENSMUSG0C	649.16872	-0.477668	0.16106947	-2.9656022	0.00302091	0.06787497	Pklr	pyruvate kinase liver and red blood cell
ENSMUSG0C	1546.4763	-0.7638628	0.17805945	-4.2899313	1.79E-05	0.00151815	Car8	carbonic anhydrase 8
ENSMUSG0C	985.780347	-0.3850804	0.12585942	-3.0596075	0.00221627	0.05536865	Cftr	cystic fibrosis transmembrane conductance regulator
ENSMUSG0C	150.440321	-1.0511734	0.3354532	-3.1335918	0.00172681	0.04683633	Cldn5	claudin 5
ENSMUSG0C	1362.55077	-0.605944	0.15397573	-3.9353217	8.31E-05	0.00516702	Entpd3	ectonucleoside triphosphate diphosphohydrolase 3
ENSMUSG0C	135.948365	-1.2502337	0.27786884	-4.4993662	6.82E-06	0.00071494	Pnpla3	patatin-like phospholipase domain containing 3
ENSMUSG0C	1334.04068	-0.5288112	0.15542436	-3.4023703	0.00066804	0.02480297	Iapp	islet amyloid polypeptide
ENSMUSG0C	518.926644	-0.5053819	0.16765842	-3.0143547	0.00257527	0.06109414	Coro2b	coronin, actin binding protein, 2B
ENSMUSG0C	542.124784	-0.5213307	0.17492487	-2.9803122	0.00287955	0.06650074	Tmem45b	transmembrane protein 45b
ENSMUSG0C	7678.58323	-0.438025	0.12486966	-3.5078575	0.00045173	0.01886328	Rptn	repetin
ENSMUSG0C	1223.53901	-0.5183783	0.14047018	-3.6903089	0.00022398	0.01111167	Osbpl6	oxysterol binding protein-like 6
ENSMUSG0C	460.846366	-0.5028299	0.18057617	-2.7845863	0.00535961	0.09992165	Mfap4	microfibrillar-associated protein 4
ENSMUSG0C	1532.69202	-0.8565229	0.10488814	-8.1660603	3.19E-16	3.70E-13	Ypel3	yippee like 3
ENSMUSG0C	2087.45544	-0.5502244	0.10925505	-5.0361464	4.75E-07	7.32E-05	Id1	inhibitor of DNA binding 1, HLH protein
ENSMUSG0C	749.430942	-0.427072	0.1379054	-3.0968479	0.0019559	0.05106586	Bex2	brain expressed X-linked 2
ENSMUSG0C	1773.4632	-0.3433744	0.10979138	-3.1275171	0.0017629	0.04766662	Foxo4	forkhead box O4
ENSMUSG0C	1300.60518	-0.6301048	0.13224407	-4.764711	1.89E-06	0.00024215	Egflam	EGF-like, fibronectin type III and laminin G domains
ENSMUSG0C	527.903586	-0.5213772	0.14743707	-3.5362692	0.00040582	0.0174483	Mei4	meiotic double-stranded break formation protein 4
ENSMUSG0C	226.667087	-0.9414092	0.24904705	-3.7800455	0.0001568	0.00838512	Ubxn10	UBX domain protein 10
ENSMUSG0C	452.895273	-1.034356	0.19438175	-5.3212608	1.03E-07	1.88E-05	Tox3	TOX high mobility group box family member 3
ENSMUSG0C	2691.86624	-0.4372585	0.15057978	-2.9038327	0.00368625	0.07818354	Mgat5b	mannoside acetylglucosaminyltransferase 5, isoenzyme B
ENSMUSG0C	70.2978876	-1.0966561	0.37880646	-2.8950301	0.00379122	0.07982655	Tas2r118	taste receptor, type 2, member 118
ENSMUSG0C	856.530364	-0.3401051	0.11613294	-2.9285842	0.0034051	0.07374744	Zrsr1	zinc finger (CCCH type), RNA binding motif and serine/arginine rich 1
ENSMUSG0C	55.43428	-1.2212132	0.41021599	-2.9770004	0.00291084	0.06692217	Prss53	protease, serine 53
ENSMUSG0C	1692.91015	-0.6439848	0.14247392	-4.5200188	6.18E-06	0.00065955	Hepacam2	HEPACAM family member 2
ENSMUSG0C	1383.02124	-0.3503193	0.1006372	-3.4810117	0.00049952	0.02022839	Krt84	keratin 84
ENSMUSG0C	6751.44514	-0.4077122	0.12616923	-3.2314708	0.00123155	0.03796574	Shisa2	shisa family member 2
ENSMUSG0C	791.549244	-0.4352789	0.14595541	-2.9822733	0.00286116	0.06634016	Dact1	dishevelled-binding antagonist of beta-catenin 1
ENSMUSG0C	1516.80428	-0.391533	0.12485682	-3.1358557	0.00171353	0.04683633	Fads6	fatty acid desaturase domain family, member 6
ENSMUSG0C	230.431582	-0.6953019	0.2180436	-3.1888206	0.00142854	0.04159741	AI467606	expressed sequence AI467606
ENSMUSG0C	3186.98388	-0.5708141	0.11432676	-4.9928305	5.95E-07	8.93E-05	Fndc7	fibronectin type III domain containing 7
ENSMUSG0C	3339.33653	-0.3062946	0.0988734	-3.0978461	0.00194933	0.05106586	Plekha7	pleckstrin homology domain containing, family A member 7
ENSMUSG0C	884.724276	-0.7190245	0.16998821	-4.2298492	2.34E-05	0.00188518	Smtnl2	smoothelin-like 2
ENSMUSG0C	892.753141	-1.302015	0.15379981	-8.4656475	2.55E-17	3.41E-14	Slitrk6	SLIT and NTRK-like family, member 6
ENSMUSG0C	1835.54542	-0.3300683	0.11124747	-2.9669732	0.00300747	0.06774365	Stard5	StAR-related lipid transfer (START) domain containing 5
ENSMUSG0C	835.048359	-0.4176527	0.11848892	-3.5248251	0.00042376	0.0179975	Eid2	EP300 interacting inhibitor of differentiation 2
ENSMUSG0C	273.875523	-0.7100265	0.23232222	-3.0562146	0.00224151	0.05583887	Gsg1l	GSG1-like
ENSMUSG0C	644.614972	-0.4964277	0.15431376	-3.2170021	0.00129538	0.03943426	Slc25a23	solute carrier family 25 (mitochondrial carrier; phosphate carrier), member 23
ENSMUSG0C	317.24843	-0.6638839	0.18320786	-3.623665	0.00029046	0.01378131	Asphd1	aspartate beta-hydroxylase domain containing 1
ENSMUSG0C	946.708349	-0.6535034	0.13701784	-4.7694772	1.85E-06	0.00023824	Camk2n1	calcium/calmodulin-dependent protein kinase II inhibitor 1
ENSMUSG0C	739.162497	-0.9922163	0.19974234	-4.9674808	6.78E-07	9.93E-05	Sh2d7	SH2 domain containing 7
ENSMUSG0C	1645.53995	-0.4306152	0.13798418	-3.1207578	0.00180386	0.04862332	Kctd4	potassium channel tetramerisation domain containing 4
ENSMUSG0C	303.539134	-0.9203557	0.2792446	-3.2958766	0.00098115	0.03261922	Slitrk4	SLIT and NTRK-like family, member 4
ENSMUSG0C	1772.63212	-0.429255	0.13423262	-3.1978438	0.00138459	0.04100325	Cystm1	cysteine-rich transmembrane module containing 1
ENSMUSG0C	10768.0513	-0.314282	0.08770421	-3.5834312	0.00033911	0.01541562	Cd24a	CD24a antigen
ENSMUSG0C	504.437217	-0.421977	0.14692973	-2.8719644	0.00407929	0.08376493	Cldn2	claudin 2
ENSMUSG0C	923.025758	-0.7534347	0.14220467	-5.2982417	1.17E-07	2.05E-05	Ptgs1	prostaglandin-endoperoxide synthase 1
ENSMUSG0C	2074.00502	-0.284638	0.09522175	-2.9892124	0.00279698	0.06546202	Tgif1	TGFB-induced factor homeobox 1
ENSMUSG0C	1685.61865	-0.3185653	0.10582578	-3.0102807	0.00261006	0.06183543	Gpr68	G protein-coupled receptor 68
ENSMUSG0C	371.780495	-0.6370881	0.16862073	-3.778231	0.00015795	0.00838512	Baiap3	BAI1-associated protein 3
ENSMUSG0C	161.086471	-0.8382602	0.29209958	-2.8697754	0.00410763	0.08404963	Cdcp2	CUB domain containing protein 2
ENSMUSG0C	144.272795	-0.6633908	0.23777803	-2.7899585	0.00527148	0.09891409	Ankfn1	ankyrin-repeat and fibronectin type III domain containing 1
ENSMUSG0C	300.157277	-0.7500107	0.24945436	-3.0066048	0.00264183	0.06224923	Cd300lf	CD300 molecule like family member F
ENSMUSG0C	301.538826	-0.7194111	0.21099916	-3.409545	0.00065071	0.02441999	Sstr2	somatostatin receptor 2
ENSMUSG0C	1791.62613	-0.5336528	0.1548877	-3.4454173	0.00057018	0.02221145	Tenm4	teneurin transmembrane protein 4
ENSMUSG0C	337.699523	-0.5564106	0.19328283	-2.8787379	0.0039927	0.08270165	Bend7	BEN domain containing 7
ENSMUSG0C	295.025104	-0.7342826	0.23084678	-3.1808223	0.00146858	0.04233829	NA	NA
ENSMUSG0C	277.751234	-0.5606898	0.1944308	-2.8837499	0.00392971	0.08165632	Nkx1-2	NK1 homeobox 2
ENSMUSG0C	393.498525	-1.3674311	0.19440877	-7.0337933	2.01E-12	1.17E-09	Pkd1l3	polycystic kidney disease 1 like 3
ENSMUSG0C	2735.20732	-0.3468379	0.08393165	-4.132385	3.59E-05	0.00266025	Zfp710	zinc finger protein 710
ENSMUSG0C	5768.44834	-0.3546546	0.09311504	-3.8087787	0.00013965	0.00767133	Bnip5	BCL2 interacting protein 5
ENSMUSG0C	1016.88595	-0.3381216	0.11836658	-2.8565631	0.00428255	0.08670779	Il23r	interleukin 23 receptor
ENSMUSG0C	280.414334	-0.7240389	0.21680279	-3.33962	0.00083893	0.02939297	Ccr2	chemokine (C-C motif) receptor 2
ENSMUSG0C	167.800748	-0.7184546	0.25319245	-2.837583	0.00454565	0.09007259	Frmpd4	FERM and PDZ domain containing 4
ENSMUSG0C	882.704492	-0.386117	0.10992618	-3.5125115	0.00044389	0.01863409	Pdp1	pyruvate dehydrogenase phosphatase catalytic subunit 1
ENSMUSG0C	345.655022	-0.5080062	0.17508958	-2.9014072	0.00371491	0.07859986	Cdc42ep1	CDC42 effector protein (Rho GTPase binding) 1
ENSMUSG0C	431.715393	-0.6749437	0.15076055	-4.4769254	7.57E-06	0.00078024	Prss33	protease, serine 33
ENSMUSG0C	1065.2729	-1.2812575	0.18298647	-7.001925	2.52E-12	1.33E-09	Cyp2g1	cytochrome P450, family 2, subfamily g, polypeptide 1
ENSMUSG0C	1710.85621	-0.6876395	0.11419289	-6.0217361	1.73E-09	5.09E-07	Grem2	gremlin 2, DAN family BMP antagonist
ENSMUSG0C	57.4553854	-1.7993284	0.50053659	-3.5947989	0.00032464	0.01487631	Gm867	predicted gene 867
ENSMUSG0C	541.831217	-0.7560537	0.16275248	-4.6454208	3.39E-06	0.0003993	Ch25h	cholesterol 25-hydroxylase

ENSMUSG0C	238.575359	-0.7444922	0.19154158	-3.8868436	0.00010156	0.00599457	Pcdh20	protocadherin 20
ENSMUSG0C	1142.96587	-0.500927	0.10695591	-4.6834908	2.82E-06	0.00034143	Fam171a1	family with sequence similarity 171, member A1
ENSMUSG0C	365.553122	-0.5985462	0.18252349	-3.279283	0.00104071	0.03432181	Ttc24	tetratricopeptide repeat domain 24
ENSMUSG0C	800.392609	-1.257428	0.16256217	-7.7350588	1.03E-14	1.06E-11	Rgs13	regulator of G-protein signaling 13
ENSMUSG0C	149.204962	-0.9258129	0.30782927	-3.0075533	0.0026336	0.06213939	Tas2r105	taste receptor, type 2, member 105
ENSMUSG0C	486.422828	-0.4923378	0.14772308	-3.3328424	0.00085964	0.03004725	Pcdh19	protocadherin 19
ENSMUSG0C	2512.81267	-0.2859441	0.08840602	-3.2344413	0.00121881	0.03780674	Crebzf	CREB/ATF bZIP transcription factor
ENSMUSG0C	649.900742	-0.487483	0.14973027	-3.2557413	0.00113097	0.03636146	Spn	sialophorin
ENSMUSG0C	1319.88002	-0.3382764	0.11762482	-2.8758929	0.00402886	0.08312157	Casr	calcium-sensing receptor
ENSMUSG0C	1460.10329	-0.4749254	0.16070876	-2.9551933	0.00312473	0.0694234	Igfn1	immunoglobulin-like and fibronectin type III domain containing 1
ENSMUSG0C	2783.6272	-0.4076223	0.12101607	-3.3683318	0.00075625	0.02720764	Klf13	Kruppel-like factor 13
ENSMUSG0C	184.094284	-0.7382202	0.21830073	-3.3816663	0.00072048	0.02630115	D630039A03	RIKEN cDNA D630039A03 gene
ENSMUSG0C	2441.01518	-1.1319888	0.3104078	-3.6467795	0.00026555	0.01284439	Cd177	CD177 antigen
ENSMUSG0C	4266.51035	-0.3308996	0.09745712	-3.3953356	0.00068545	0.02523395	Dlg2	discs large MAGUK scaffold protein 2
ENSMUSG0C	1355.55683	-1.2233382	0.16576479	-7.379964	1.58E-13	1.25E-10	Sh2d6	SH2 domain containing 6
ENSMUSG0C	2961.47567	-0.5584629	0.14992544	-3.7249373	0.00019536	0.00988915	Cyp2f2	cytochrome P450, family 2, subfamily f, polypeptide 2
ENSMUSG0C	131.804834	-1.0698081	0.35002281	-3.0563952	0.00224016	0.05583887	Gm609	predicted gene 609
ENSMUSG0C	322.089422	-0.7777656	0.1894898	-4.1045248	4.05E-05	0.00293333	Nrgn	neurogranin
ENSMUSG0C	2844.84761	-0.2931365	0.09672475	-3.0306252	0.00244048	0.05902233	Cbx7	chromobox 7
ENSMUSG0C	461.477352	-0.698692	0.19977572	-3.4973821	0.00046985	0.01934153	Tnfaip6	tumor necrosis factor alpha induced protein 6
ENSMUSG0C	4415.21358	-0.3380348	0.08938028	-3.7819843	0.00015558	0.00838512	Tcf4	transcription factor 4
ENSMUSG0C	949.957882	-0.6323816	0.16982703	-3.7236803	0.00019634	0.00990974	Klk1b26	kallikrein 1-related petidase b26
ENSMUSG0C	1091.28342	-0.3420193	0.11147571	-3.0681058	0.0021542	0.05405064	Camk2d	calcium/calmodulin-dependent protein kinase II, delta
ENSMUSG0C	389.019951	-0.4689469	0.16009473	-2.9291841	0.00339853	0.07374744	Shisa6	shisa family member 6
ENSMUSG0C	1028.6993	-0.3918579	0.12093216	-3.2403116	0.00119399	0.0372598	Nt5dc3	5'-nucleotidase domain containing 3
ENSMUSG0C	6364.796	-0.3579164	0.12567387	-2.8479782	0.00439979	0.08796053	Spock3	sparc/osteonectin, cwcv and kazal-like domains proteoglycan 3
ENSMUSG0C	6181.10868	-0.2805052	0.10040385	-2.793769	0.00520977	0.09861739	Msi1	musashi RNA-binding protein 1
ENSMUSG0C	2450.28526	-0.5980466	0.15822645	-3.7796879	0.00015703	0.00838512	Cadm4	cell adhesion molecule 4
ENSMUSG0C	1732.11515	-0.3179213	0.1137919	-2.7938837	0.00520792	0.09861739	Arntl	aryl hydrocarbon receptor nuclear translocator-like
ENSMUSG0C	3432.28488	-0.325184	0.10925521	-2.9763702	0.00291682	0.06692217	Tmem245	transmembrane protein 245
ENSMUSG0C	940.856662	-0.5247536	0.15403538	-3.4067086	0.00065751	0.02462209	Zcchc24	zinc finger, CCHC domain containing 24
ENSMUSG0C	567.006552	-0.4731016	0.15961171	-2.9640781	0.00303591	0.06812414	Tmem191c	transmembrane protein 191C
ENSMUSG0C	1160.23675	-0.6126246	0.1381016	-4.4360424	9.16E-06	0.00092226	Fut2	fucosyltransferase 2
ENSMUSG0C	701.264846	-0.5453995	0.16282198	-3.3496675	0.00080909	0.02869372	Irs1	insulin receptor substrate 1
ENSMUSG0C	231.418245	-0.6647654	0.2328719	-2.8546401	0.00430856	0.08693512	Sag	S-antigen, retina and pineal gland (arrestin)
ENSMUSG0C	320.380497	-0.8918059	0.21491214	-4.1496301	3.33E-05	0.0025212	Myh1	myosin, heavy polypeptide 1, skeletal muscle, adult
ENSMUSG0C	710.284258	-0.427273	0.13161548	-3.2463734	0.00116885	0.03685519	Slit3	slit guidance ligand 3
ENSMUSG0C	1249.25772	-0.737631	0.15634029	-4.7181124	2.38E-06	0.00030037	Ptprn2	protein tyrosine phosphatase, receptor type, N polypeptide 2
ENSMUSG0C	67.9461182	-2.0097516	0.43504696	-4.61962	3.84E-06	0.00044333	Tas2r102	taste receptor, type 2, member 102
ENSMUSG0C	145.177059	-1.0457102	0.30131111	-3.4705331	0.00051943	0.02074489	Gja5	gap junction protein, alpha 5
ENSMUSG0C	1105.13075	-0.6209828	0.19484967	-3.1869842	0.00143765	0.04165346	Saa2	serum amyloid A 2
ENSMUSG0C	1218.64182	-0.6243458	0.14281456	-4.3717238	1.23E-05	0.00116026	Ces5a	carboxylesterase 5A
ENSMUSG0C	60.2505495	-1.4584017	0.43280072	-3.3696841	0.00075254	0.02713054	Zscan5b	zinc finger and SCAN domain containing 5B
ENSMUSG0C	2291.96733	-0.5288375	0.1350256	-3.9165726	8.98E-05	0.00541169	Sez6l	seizure related 6 homolog like
ENSMUSG0C	8442.8395	-0.2330648	0.08183086	-2.8481283	0.00439772	0.08796053	Spock2	sparc/osteonectin, cwcv and kazal-like domains proteoglycan 2
ENSMUSG0C	601.674195	-0.4546364	0.15399249	-2.9523286	0.00315387	0.0699597	Kcnc1	potassium voltage gated channel, Shaw-related subfamily, member 1
ENSMUSG0C	68.6609298	-1.3196761	0.43454766	-3.0368962	0.00239028	0.05829398	Ifnl2	interferon lambda 2
ENSMUSG0C	791.708643	-0.6651408	0.18644159	-3.567556	0.00036033	0.01604696	Ntrk3	neurotrophic tyrosine kinase, receptor, type 3
ENSMUSG0C	1008.07422	-0.4198971	0.11635988	-3.6086072	0.00030785	0.01425667	Hspa2	heat shock protein 2
ENSMUSG0C	612.194119	-1.2267924	0.20440214	-6.001857	1.95E-09	5.57E-07	Alox5ap	arachidonate 5-lipoxygenase activating protein
ENSMUSG0C	1278.817	-0.3809363	0.10913376	-3.4905452	0.00048204	0.0197035	Sntb1	syntrophin, basic 1
ENSMUSG0C	166.757736	-0.6473421	0.2317123	-2.7937323	0.00521036	0.09861739	Intu	inturned planar cell polarity protein
ENSMUSG0C	1489.1333	-0.3211313	0.10431112	-3.0785909	0.00207982	0.05294725	Taok3	TAO kinase 3
ENSMUSG0C	388.690155	-0.6394986	0.22017845	-2.9044559	0.00367892	0.07812323	Tmem236	transmembrane protein 236
ENSMUSG0C	2515.21344	-0.6907619	0.11880303	-5.8143454	6.09E-09	1.51E-06	Dpp6	dipeptidylpeptidase 6
ENSMUSG0C	292.841043	-1.1161491	0.25899845	-4.3094817	1.64E-05	0.0014391	Myl1	myosin, light polypeptide 1
ENSMUSG0C	5244.97798	-0.4370048	0.12023434	-3.6346087	0.0002784	0.0133549	Serpinb2	serine (or cysteine) peptidase inhibitor, clade B, member 2
ENSMUSG0C	389.102668	-0.5361666	0.18779022	-2.855136	0.00430184	0.08690017	Ap3b2	adaptor-related protein complex 3, beta 2 subunit
ENSMUSG0C	1352.29326	-0.3623648	0.12458183	-2.9086493	0.00362994	0.0773661	Shisal1	shisa like 1
ENSMUSG0C	3670.90765	-0.3036921	0.0981237	-3.0949921	0.00196818	0.05127252	Mapk3	mitogen-activated protein kinase 3
ENSMUSG0C	1810.85855	-0.4308668	0.13618342	-3.1638713	0.00155686	0.04436914	Usp31	ubiquitin specific peptidase 31
ENSMUSG0C	372.696814	-0.746671	0.19370966	-3.8545885	0.00011592	0.00661834	Sox11	SRY (sex determining region Y)-box 11
ENSMUSG0C	554.646405	-0.4840263	0.17264446	-2.803602	0.00505352	0.09659388	Mapk15	mitogen-activated protein kinase 15
ENSMUSG0C	1951.14885	-0.6975306	0.15118909	-4.6136307	3.96E-06	0.00045288	Chil4	chitinase-like 4
ENSMUSG0C	1873.2711	-0.7080042	0.15887146	-4.4564593	8.33E-06	0.00084356	Klk1	kallikrein 1
ENSMUSG0C	383.312539	-0.674134	0.18641106	-3.6163839	0.00029875	0.01404203	Mro	maestro
ENSMUSG0C	1303.5375	-0.4533094	0.16019293	-2.8297716	0.00465812	0.09144523	Fbln2	fibulin 2
ENSMUSG0C	387.358474	-0.5762512	0.20231378	-2.8483044	0.00439529	0.08796053	Krt2	keratin 2
ENSMUSG0C	973.047158	-0.3864221	0.13478056	-2.867046	0.00414323	0.08467843	Ethe1	ethylmalonic encephalopathy 1
ENSMUSG0C	916.595158	-0.4208327	0.12595334	-3.341179	0.00083423	0.02928734	Gimap8	GTPase, IMAP family member 8
ENSMUSG0C	668.757002	-0.5663852	0.1594406	-3.5523275	0.00038184	0.01662242	Zfp428	zinc finger protein 428
ENSMUSG0C	2435.53526	-0.7258508	0.15217828	-4.76974	1.84E-06	0.00023824	Selenop	selenoprotein P
ENSMUSG0C	2298.01476	-0.2896583	0.10043507	-2.8840358	0.00392614	0.08165632	Phactr4	phosphatase and actin regulator 4
ENSMUSG0C	244.460605	-0.6014334	0.21345533	-2.8176076	0.00483829	0.09392324	Klk1b3	kallikrein 1-related peptidase b3
ENSMUSG0C	1741.40498	-0.8105793	0.1098294	-7.3803488	1.58E-13	1.25E-10	Fxyd6	FXYD domain-containing ion transport regulator 6
ENSMUSG0C	406.840152	-1.2897226	0.20301452	-6.3528589	2.11E-10	7.67E-08	Cldn9	claudin 9
ENSMUSG0C	767.350278	-0.5150771	0.17853343	-2.8850459	0.00391357	0.08161309	Serpinb7	serine (or cysteine) peptidase inhibitor, clade B, member 7
ENSMUSG0C	277.832547	-0.7741669	0.19257714	-4.0200354	5.82E-05	0.00391217	Col4a4	collagen, type IV, alpha 4
ENSMUSG0C	727.726129	-0.8036277	0.14115821	-5.6930997	1.25E-08	2.79E-06	Tspan6	tetraspanin 6
ENSMUSG0C	143.817383	-1.3066964	0.30191249	-4.3280633	1.50E-05	0.00135719	A630073D07	RIKEN cDNA A630073D07 gene
ENSMUSG0C	2076.69251	-0.639718	0.15097966	-4.2371141	2.26E-05	0.0018347	Sptbn2	spectrin beta, non-erythrocytic 2
ENSMUSG0C	393.091138	-0.580211	0.18474618	-3.1405845	0.00168611	0.04663973	Col8a1	collagen, type VIII, alpha 1
ENSMUSG0C	2323.10853	-0.3461518	0.11869218	-2.9163825	0.00354116	0.0759387	Usf3	upstream transcription factor family member 3
ENSMUSG0C	520.407957	-0.6746473	0.20810817	-3.2418107	0.00118773	0.03719771	Actc1	actin, alpha, cardiac muscle 1
ENSMUSG0C	5770.2668	-0.3647436	0.113329	-3.218449	0.00128886	0.03930455	Lyz1	lysozyme 1
ENSMUSG0C	2318.6633	-0.2821259	0.09070492	-3.1103708	0.00186853	0.04975022	Tlnrd1	talin rod domain containing 1
ENSMUSG0C	181.77581	-0.9311167	0.25585342	-3.6392585	0.00027342	0.01315231	Cited4	Cbp/p300-interacting transactivator, with Glu/Asp-rich carboxy-terminal domain, 4
ENSMUSG0C	58.7260323	-1.1571858	0.38342402	-3.0180315	0.00254422	0.06056476	Tas2r115	taste receptor, type 2, member 115
ENSMUSG0C	2771.17177	-0.3707376	0.12615509	-2.9387447	0.00329544	0.07199947	Arhgef10	Rho guanine nucleotide exchange factor (GEF) 10
ENSMUSG0C	180.151665	-0.9481883	0.26665083	-3.5559174	0.00037666	0.01647945	Ect2l	epithelial cell transforming sequence 2 oncogene-like
ENSMUSG0C	138.62818	-1.4032569	0.30633549	-4.5807845	4.63E-06	0.00051707	Gm4952	predicted gene 4952
ENSMUSG0C	220.984746	-0.8482669	0.28827109	-2.9426013	0.00325467	0.07146737	Nanos1	nanos C2HC-type zinc finger 1
ENSMUSG0C	743.778423	-0.4820129	0.15114907	-3.18899	0.00142771	0.04159741	Gpr27	G protein-coupled receptor 27
ENSMUSG0C	216.09469	-0.981893	0.24775742	-3.9631224	7.40E-05	0.00478863	Mucl3	mucin like 3
ENSMUSG0C	386.775079	-0.8193627	0.20584653	-3.9804545	6.88E-05	0.00451973	Klhl40	kelch-like 40
ENSMUSG0C	1270.86254	-0.4990652	0.17286118	-2.8870866	0.00388827	0.08118282	Adh1	alcohol dehydrogenase 1 (class I)
ENSMUSG0C	534.283257	-0.76022	0.16920851	-4.4928	7.03E-06	0.00072858	Ceacam1	carcinoembryonic antigen-related cell adhesion molecule 1
ENSMUSG0C	609.785454	-0.9225177	0.23498317	-3.9258883	8.64E-05	0.0052981	Fat3	FAT atypical cadherin 3
ENSMUSG0C	483.225389	-0.5657433	0.16410379	-3.4474722	0.00056586	0.02209589	Mcidas	multiciliate differentiation and DNA synthesis associated cell cycle protein
ENSMUSG0C	281.331287	-0.7959101	0.20838981	-3.819333	0.00013381	0.00746823	Malrd1	MAM and LDL receptor class A domain containing 1
ENSMUSG0C	4830.86764	-0.3928317	0.11945358	-3.2885722	0.00100697	0.03333529	Nrbp2	nuclear receptor binding protein 2
ENSMUSG0C	416.640179	-0.5741166	0.20070146	-2.8605499	0.00422907	0.08582842	Erich3	glutamate rich 3
ENSMUSG0C	4722.09477	-0.4723246	0.12519889	-3.7725943	0.00016156	0.00850692	Ctdsp2	CTD (carboxy-terminal domain, RNA polymerase II, polypeptide A) small phosphatase 2
ENSMUSG0C	573.75923	-0.4831106	0.16522404	-2.9239726	0.00345595	0.07457061	Cfap74	cilia and flagella associated protein 74
ENSMUSG0C	463.975042	-0.454232	0.14199375	-3.1989575	0.00137925	0.04098458	Fam174b	family with sequence similarity 174, member B
ENSMUSG0C	232.868384	-0.8819894	0.21662993	-4.0714105	4.67E-05	0.00325235	Ccr5	chemokine (C-C motif) receptor 5
ENSMUSG0C	478.584214	-0.5348545	0.16813284	-3.1811422	0.00146696	0.04233829	Ackr4	atypical chemokine receptor 4
ENSMUSG0C	256.687567	-0.8844859	0.20020901	-4.4178124	9.97E-06	0.00098618	Nhsl2	NHS-like 2
ENSMUSG0C	484.895691	-0.474719	0.15455068	-3.0716073	0.0021291	0.05380836	Aox2	aldehyde oxidase 2
ENSMUSG0C	130.170131	-0.84544	0.28555366	-2.9607043	0.00306936	0.06878616	NA	NA
ENSMUSG0C	983.478209	-0.5289661	0.12788794	-4.1361688	3.53E-05	0.00263925	Crocc2	ciliary rootlet coiled-coil, rootletin family member 2
ENSMUSG0C	179.873236	-0.8740369	0.24354311	-3.5888384	0.00033215	0.0151806	E030013I19R	RIKEN cDNA E030013I19 gene
ENSMUSG0C	359.666215	-0.4973306	0.1749823	-2.8421763	0.00448067	0.08916792	2610035D17	RIKEN cDNA 2610035D17 gene
ENSMUSG0C	209.74948	-0.6598937	0.21647967	-3.0482941	0.00230145	0.05708701	E130317F20	RIKEN cDNA E130317F20 gene
ENSMUSG0C	331.146595	-0.5558241	0.17695602	-3.1410297	0.00168355	0.04663973	NA	NA
ENSMUSG0C	1608.11648	-0.410752	0.11848071	-3.4668263	0.00052664	0.02093703	Miat	myocardial infarction associated transcript (non-protein coding)
ENSMUSG0C	454.338783	-0.5586114	0.19914667	-2.8050251	0.00503127	0.09627409	Rassf10	Ras association (RalGDS/AF-6) domain family (N-terminal) member 10
ENSMUSG0C	473.377206	-0.8705429	0.20352295	-4.2773699	1.89E-05	0.0015964	Rgs21	regulator of G-protein signalling 21
ENSMUSG0C	20197.9671	-0.2070656	0.07117522	-2.9092378	0.00362311	0.07731525	Kctd12	potassium channel tetramerisation domain containing 12
ENSMUSG0C	304.004697	-1.3890239	0.25580823	-5.4299422	5.64E-08	1.10E-05	6030407O03	RIKEN cDNA 6030407O03 gene
ENSMUSG0C	232.546198	-1.1845056	0.253565	-4.6714082	2.99E-06	0.00035924	Ovol3	ovo like zinc finger 3
ENSMUSG0C	598.63191	-0.5972855	0.2044043	-2.9220786	0.00347704	0.07493274	Flg	filaggrin
ENSMUSG0C	82.7680027	-1.2572802	0.32099393	-3.9168349	8.97E-05	0.00541169	NA	NA
ENSMUSG0C	111.395164	-1.1815162	0.28697722	-4.1171078	3.84E-05	0.00281882	Gm19026	calmodulin regulated spectrin-associated protein 1 pseudogene
ENSMUSG0C	150.376898	-1.2736041	0.3201014	-3.9787521	6.93E-05	0.0045351	NA	NA
ENSMUSG0C	73.2274389	-1.4934689	0.36303196	-4.1138772	3.89E-05	0.00284658	Tas2r142-ps	taste receptor, type 2, member 142, pseudogene 5
ENSMUSG0C	266.052967	-0.7813204	0.24729325	-3.1594894	0.00158046	0.04474882	5430431A17	RIKEN cDNA 5430431A17 gene
ENSMUSG0C	108.312259	-1.0009982	0.32477509	-3.0821272	0.00205527	0.052694	NA	NA
ENSMUSG0C	188.718925	-0.778548	0.24825908	-3.1360302	0.00171252	0.04683633	Gm6249	predicted gene 6249
ENSMUSG0C	102.93311	-1.0871438	0.33227791	-3.2717907	0.00106869	0.03504528	Rnf223	ring finger 223
ENSMUSG0C	530.250107	-0.5490524	0.16532942	-3.32096	0.00089708	0.0308105	Gm8714	interferon inducible GTPase 1 pseudogene

Table 4. Upregulated in cKO mTEC

row	baseMean	log2FoldChai	lfcSE	stat	pvalue	padj	symbol	geneName
ENSMUSG0C	248.784604	0.67900503	0.20630747	3.29122853	0.00099751	0.03308498	Wnt9a	wingless-type MMTV integration site family, member 9A
ENSMUSG0C	37648.8113	0.37322502	0.09128367	4.08862839	4.34E-05	0.00305913	Ccnd2	cyclin D2
ENSMUSG0C	1270.68134	0.46379886	0.11927174	3.88858956	0.00010083	0.00597186	Drp2	dystrophin related protein 2
ENSMUSG0C	168.652322	0.86951595	0.25943969	3.35151477	0.00080371	0.02856114	Tssk3	testis-specific serine kinase 3
ENSMUSG0C	28101.9167	0.31325586	0.06767648	4.62872534	3.68E-06	0.00042711	Icosl	icos ligand
ENSMUSG0C	6525.79171	0.29975184	0.07394261	4.05384452	5.04E-05	0.00344045	Slc7a7	solute carrier family 7 (cationic amino acid transporter, y+ system), member 7
ENSMUSG0C	3181.78051	0.4458609	0.10220187	4.36255126	1.29E-05	0.00119707	S100a3	S100 calcium binding protein A3
ENSMUSG0C	759.24386	0.39979357	0.14135155	2.82836345	0.00467867	0.09164185	B9d1	B9 protein domain 1
ENSMUSG0C	6821.14491	6.23274608	0.10328413	60.3456295	0	0	Slc13a2	solute carrier family 13 (sodium-dependent dicarboxylate transporter), member 2
ENSMUSG0C	5586.86937	0.50023527	0.08920551	5.60767255	2.05E-08	4.35E-06	Mcm3ap	minichromosome maintenance complex component 3 associated protein
ENSMUSG0C	68.0594647	1.26117411	0.41411286	3.04548405	0.00232306	0.05720062	Nkx2-1	NK2 homeobox 1
ENSMUSG0C	1256.20788	0.43805327	0.14740673	2.97173178	0.00296125	0.06715766	Gstt1	glutathione S-transferase, theta 1
ENSMUSG0C	8628.79468	0.32945996	0.06902237	4.77323433	1.81E-06	0.00023735	Aif1l	allograft inflammatory factor 1-like
ENSMUSG0C	4202.96284	0.45430086	0.08438572	5.38362266	7.30E-08	1.40E-05	Gria3	glutamate receptor, ionotropic, AMPA3 (alpha 3)
ENSMUSG0C	2070.03893	0.24534545	0.08624793	2.84465324	0.00444598	0.08868023	Naa20	N(alpha)-acetyltransferase 20, NatB catalytic subunit
ENSMUSG0C	4789.52907	0.45723785	0.10110626	4.5223498	6.12E-06	0.00065736	Bax	BCL2-associated X protein
ENSMUSG0C	2259.22798	0.4035225	0.11421404	3.53303747	0.00041081	0.01753311	Brinp2	bone morphogenic protein/retinoic acid inducible neural-specific 2
ENSMUSG0C	5708.19989	0.27245084	0.08640678	3.1531189	0.00161536	0.04543084	Eno2	enolase 2, gamma neuronal
ENSMUSG0C	8141.24847	0.30635761	0.10971703	2.79225205	0.00523426	0.09885478	Grap	GRB2-related adaptor protein
ENSMUSG0C	8180.70333	0.4557348	0.11314764	4.02778895	5.63E-05	0.00380007	Cd44	CD44 antigen
ENSMUSG0C	1393.58794	1.00276937	0.10198414	9.83260085	8.15E-23	1.77E-19	Spc25	SPC25, NDC80 kinetochore complex component, homolog (S. cerevisiae)
ENSMUSG0C	1200.4561	0.4632209	0.13979122	3.31366233	0.00092083	0.03143867	Fblim1	filamin binding LIM protein 1
ENSMUSG0C	7233.36887	0.29455195	0.08225069	3.58114866	0.00034209	0.01541562	Susd2	sushi domain containing 2
ENSMUSG0C	799.609983	0.4941355	0.15392471	3.21024151	0.00132623	0.03990957	Adamts4	a disintegrin-like and metallopeptidase (reprolysin type) with thrombospondin type 1 motif, 4
ENSMUSG0C	3446.41307	0.35661008	0.08923607	3.99625482	6.44E-05	0.00427698	Epha2	Eph receptor A2
ENSMUSG0C	1276.22422	0.31990455	0.11479053	2.78685484	0.00532223	0.09954459	Pdk1	pyruvate dehydrogenase kinase, isoenzyme 1
ENSMUSG0C	3982.5527	0.58556483	0.09895486	5.91749433	3.27E-09	8.89E-07	Sulf2	sulfatase 2
ENSMUSG0C	8602.17859	0.21900012	0.07191707	3.04517588	0.00232544	0.05720062	Clic1	chloride intracellular channel 1
ENSMUSG0C	1705.12567	0.89731198	0.10548876	8.50623271	1.80E-17	2.61E-14	Cpt1c	carnitine palmitoyltransferase 1c
ENSMUSG0C	190.281119	0.99556241	0.25823846	3.85520582	0.00011563	0.00661834	Hmgcll1	3-hydroxymethyl-3-methylglutaryl-Coenzyme A lyase-like 1
ENSMUSG0C	1974.80327	0.57257203	0.10810139	5.29662054	1.18E-07	2.05E-05	Fbxw9	F-box and WD-40 domain protein 9
ENSMUSG0C	17862.4902	0.27648486	0.08554378	3.232086	0.0012289	0.03796574	Rps18	ribosomal protein S18
ENSMUSG0C	19377.2333	0.2954743	0.07870119	3.75438137	0.00017377	0.00903242	Lpo	lactoperoxidase
ENSMUSG0C	1231.80302	0.35409432	0.11462571	3.08913525	0.0020074	0.05178498	Met	met proto-oncogene
ENSMUSG0C	29381.7823	0.23719663	0.08145394	2.91203388	0.00359084	0.07681479	Apobec3	apolipoprotein B mRNA editing enzyme, catalytic polypeptide 3
ENSMUSG0C	6152.59393	0.27431795	0.09489377	2.89078984	0.00384275	0.08061904	Nedd8	neural precursor cell expressed, developmentally down-regulated gene 8
ENSMUSG0C	3756.28498	0.24116893	0.07792871	3.09473802	0.00196987	0.05127252	Vac14	Vac14 homolog (S. cerevisiae)
ENSMUSG0C	1650.91281	0.54360792	0.13826571	3.93161779	8.44E-05	0.00521008	Glis2	GLIS family zinc finger 2
ENSMUSG0C	2070.11168	0.30275965	0.10439174	2.90022609	0.00372894	0.07861012	Srp19	signal recognition particle 19
ENSMUSG0C	4237.25586	0.28204991	0.09087886	3.10358095	0.00191194	0.05036702	Aldh1a3	aldehyde dehydrogenase family 1, subfamily A3
ENSMUSG0C	11663.0822	0.26593811	0.07789606	3.41401233	0.00064014	0.02414943	Gadd45b	growth arrest and DNA-damage-inducible 45 beta
ENSMUSG0C	7417.09836	0.23983872	0.06885598	3.48319357	0.00049547	0.02014715	Psma2	proteasome subunit alpha 2
ENSMUSG0C	401.968396	0.61584446	0.17786455	3.46243508	0.00053531	0.02123319	Tenm1	teneurin transmembrane protein 1
ENSMUSG0C	21995.9229	0.35060447	0.11417823	3.07067692	0.00213574	0.05382006	Ctsz	cathepsin Z
ENSMUSG0C	3567.75927	0.27926189	0.08604661	3.24547236	0.00117256	0.03685519	Glod4	glyoxalase domain containing 4
ENSMUSG0C	7966.53088	0.25143095	0.08097893	3.10489358	0.00190347	0.05029622	Psmb6	proteasome (prosome, macropain) subunit, beta type 6
ENSMUSG0C	32125.6407	0.24439189	0.08262305	2.95791417	0.00309728	0.06896803	Pfn1	profilin 1
ENSMUSG0C	860.493321	0.33553249	0.11999699	2.79617433	0.00517115	0.09841009	Trpv2	transient receptor potential cation channel, subfamily V, member 2
ENSMUSG0C	8861.69104	0.31695925	0.10494741	3.02017207	0.00252631	0.06026117	Ikzf1	IKAROS family zinc finger 1
ENSMUSG0C	31318.2372	0.39146113	0.11207261	3.49292417	0.00047776	0.01962093	Myh11	myosin, heavy polypeptide 11, smooth muscle
ENSMUSG0C	2024.61352	0.35019286	0.12386939	2.82711366	0.00469697	0.09189692	Noxo1	NADPH oxidase organizer 1
ENSMUSG0C	5233.51448	0.26485024	0.09381166	2.82321243	0.00475451	0.0926065	Rgs17	regulator of G-protein signaling 17
ENSMUSG0C	15857.8806	0.24784495	0.08502801	2.91486236	0.00355845	0.0762157	Crybg1	crystallin beta-gamma domain containing 1
ENSMUSG0C	1518.65109	0.35899547	0.10853164	3.30774931	0.00094049	0.03186137	Kitl	kit ligand
ENSMUSG0C	4797.2474	0.69366614	0.105724	6.5611038	5.34E-11	2.33E-08	Igf1	insulin-like growth factor 1
ENSMUSG0C	3689.11368	0.30330172	0.08781189	3.45399383	0.00055235	0.02176034	Snrpd3	small nuclear ribonucleoprotein D3
ENSMUSG0C	7509.5214	0.31481654	0.0732951	4.29519204	1.75E-05	0.00148985	Mdm2	transformed mouse 3T3 cell double minute 2
ENSMUSG0C	2607.17348	0.32545833	0.09084386	3.58261236	0.00034018	0.01541562	Cdc34	cell division cycle 34
ENSMUSG0C	12221.6129	0.9206719	0.09189051	10.019227	1.25E-23	3.12E-20	Ccng1	cyclin G1
ENSMUSG0C	1863.82878	0.30193731	0.10517785	2.87073091	0.00409524	0.0838946	Nudcd2	NudC domain containing 2
ENSMUSG0C	31757.3783	0.19885219	0.06344895	3.13405011	0.00172411	0.04683633	Tnip1	TNFAIP3 interacting protein 1
ENSMUSG0C	2278.80106	0.3163877	0.10154824	3.11563942	0.00183547	0.04907061	Supt4a	SPT4A, DSIF elongation factor subunit
ENSMUSG0C	9180.85612	0.27331463	0.07978621	3.42558716	0.00061347	0.02347778	Nup85	nucleoporin 85
ENSMUSG0C	24461.6324	0.32567275	0.08985984	3.62423027	0.00028982	0.01378131	Itgb4	integrin beta 4
ENSMUSG0C	13602.107	0.41110211	0.08748247	4.69925113	2.61E-06	0.00032247	Nos2	nitric oxide synthase 2, inducible
ENSMUSG0C	926.731864	0.42112599	0.14981117	2.81104528	0.00493808	0.09480358	Krt24	keratin 24
ENSMUSG0C	4165.80074	0.29121886	0.09587818	3.03738416	0.00238641	0.05829398	Gtf2a1	general transcription factor II A, 1
ENSMUSG0C	10248.9161	0.30181698	0.09020181	3.34601904	0.00081981	0.02889739	Psma6	proteasome subunit alpha 6
ENSMUSG0C	44861.262	0.22967759	0.07759499	2.95995377	0.00307685	0.06886533	Nfkbia	nuclear factor of kappa light polypeptide gene enhancer in B cells inhibitor, alpha
ENSMUSG0C	7327.22967	0.2175508	0.07299282	2.98044128	0.00287833	0.06650074	Psmc1	protease (prosome, macropain) 26S subunit, ATPase 1
ENSMUSG0C	1913.96515	1.02700917	0.10108482	10.1598751	2.99E-24	8.69E-21	Dglucy	D-glutamate cyclase
ENSMUSG0C	1317.94337	0.48335549	0.115077	4.2002789	2.67E-05	0.00209102	Dcaf4	DDB1 and CUL4 associated factor 4
ENSMUSG0C	2223.55738	0.36976599	0.10460402	3.53491178	0.00040791	0.01745982	Eci2	enoyl-Coenzyme A delta isomerase 2
ENSMUSG0C	6597.92332	0.27722213	0.07739544	3.58189252	0.00034111	0.01541562	Syk	spleen tyrosine kinase
ENSMUSG0C	678.516406	0.66081715	0.14172221	4.66276355	3.12E-06	0.00036957	Slc25a48	solute carrier family 25, member 48
ENSMUSG0C	1646.07261	0.61119535	0.11525176	5.30313251	1.14E-07	2.02E-05	Polk	polymerase (DNA directed), kappa
ENSMUSG0C	8553.7073	0.46655983	0.11910238	3.9173005	8.95E-05	0.00541169	F2rl1	coagulation factor II (thrombin) receptor-like 1
ENSMUSG0C	6980.28347	0.68802894	0.09895343	6.95305821	3.57E-12	1.78E-09	Crhbp	corticotropin releasing hormone binding protein
ENSMUSG0C	2746.99886	0.52495141	0.09209022	5.70040371	1.20E-08	2.76E-06	Plau	plasminogen activator, urokinase
ENSMUSG0C	4099.35866	0.30278566	0.07377729	4.10404967	4.06E-05	0.00293333	Tdh	L-threonine dehydrogenase
ENSMUSG0C	39118.2084	0.39111688	0.06435437	6.07754936	1.22E-09	3.79E-07	Clu	clusterin
ENSMUSG0C	3684.90648	0.41176595	0.08731914	4.71564341	2.41E-06	0.00030184	Tnfrsf10b	tumor necrosis factor receptor superfamily, member 10b
ENSMUSG0C	6862.6406	0.32903587	0.08163339	4.03065306	5.56E-05	0.00376867	Bmp1	bone morphogenetic protein 1
ENSMUSG0C	23163.117	0.25005467	0.07940273	3.14919487	0.00163721	0.04568707	Ankrd33b	ankyrin repeat domain 33B
ENSMUSG0C	8019.76473	0.39176872	0.09226556	4.24609913	2.18E-05	0.00179514	Rnf19a	ring finger protein 19A
ENSMUSG0C	4603.82956	0.37998027	0.11104324	3.42191269	0.00062182	0.02369322	Has2	hyaluronan synthase 2
ENSMUSG0C	1361.11547	0.68364845	0.11930219	5.73039299	1.00E-08	2.36E-06	Gtse1	G two S phase expressed protein 1
ENSMUSG0C	5194.64713	0.29421616	0.08615657	3.41490113	0.00063805	0.02414943	Slc25a17	solute carrier family 25 (mitochondrial carrier, peroxisomal membrane protein), member 17
ENSMUSG0C	11907.6769	0.34669975	0.0805485	4.3042357	1.68E-05	0.00144677	C1qtnf6	C1q and tumor necrosis factor related protein 6
ENSMUSG0C	3093.61202	0.31752847	0.08521761	3.72608979	0.00019447	0.00988915	Nubp1	nucleotide binding protein 1
ENSMUSG0C	808.797589	0.5914141	0.13852761	4.26928688	1.96E-05	0.00164167	Gpt	glutamic pyruvic transaminase, soluble
ENSMUSG0C	7115.20612	0.22758995	0.07306861	3.11474316	0.00184105	0.04909376	Gsdmd	gasdermin D
ENSMUSG0C	1112.94867	0.53344817	0.17019344	3.1343638	0.00172227	0.04683633	Ly6i	lymphocyte antigen 6 complex, locus I
ENSMUSG0C	1753.40056	0.30359369	0.10681307	2.84228977	0.00447908	0.08916792	Arl6	ADP-ribosylation factor-like 6
ENSMUSG0C	3926.58276	0.25411825	0.09084339	2.79732225	0.00515281	0.09816838	Klhl22	kelch-like 22
ENSMUSG0C	3369.92003	0.30331339	0.10541914	2.87721378	0.00401204	0.08287259	Abcc5	ATP-binding cassette, sub-family C (CFTR/MRP), member 5
ENSMUSG0C	1102.91155	0.35548753	0.1241694	2.8629239	0.00419751	0.08555851	Samsn1	SAM domain, SH3 domain and nuclear localization signals, 1
ENSMUSG0C	1541.05469	0.34517694	0.11248513	3.06864512	0.00215032	0.05405064	Zfp641	zinc finger protein 641
ENSMUSG0C	17716.2483	0.47504527	0.06762543	7.02465388	2.15E-12	1.21E-09	Slc4a8	solute carrier family 4 (anion exchanger), member 8
ENSMUSG0C	7262.93403	0.67321395	0.13496905	4.98791363	6.10E-07	9.08E-05	Sncg	synuclein, gamma
ENSMUSG0C	10340.5319	1.07325655	0.08808334	12.1845581	3.76E-34	1.64E-30	Cdkn1a	cyclin-dependent kinase inhibitor 1A (P21)
ENSMUSG0C	13819.4166	0.27438242	0.09357429	2.93224165	0.00336525	0.0733405	Crip3	cysteine-rich protein 3
ENSMUSG0C	11254.8617	0.27636463	0.09750334	2.83441184	0.00459101	0.0905755	Psmb8	proteasome (prosome, macropain) subunit, beta type 8 (large multifunctional peptidase 7)
ENSMUSG0C	6961.87904	0.28038754	0.09327374	3.00607156	0.00264647	0.06227423	Cdc23	CDC23 cell division cycle 23
ENSMUSG0C	730.684743	0.42179225	0.12009076	3.51227888	0.00044428	0.01863409	Tmem216	transmembrane protein 216
ENSMUSG0C	5893.9182	0.32018949	0.09514073	3.36543019	0.00076424	0.02743872	Fas	Fas (TNF receptor superfamily member 6)
ENSMUSG0C	10406.3304	0.6225077	0.0894707	6.95767072	3.46E-12	1.77E-09	Dnase1l3	deoxyribonuclease 1-like 3
ENSMUSG0C	16148.9308	0.25225673	0.08954566	2.81707385	0.00484634	0.09397471	Cd63	CD63 antigen
ENSMUSG0C	3204.16549	0.36218403	0.08183938	4.42554694	9.62E-06	0.0009627	Baiap2	brain-specific angiogenesis inhibitor 1-associated protein 2
ENSMUSG0C	5903.84127	0.27132047	0.08944215	3.03347443	0.00241755	0.05879446	Fam20c	FAM20C, golgi associated secretory pathway kinase
ENSMUSG0C	4723.77265	0.34667308	0.09618928	3.60407191	0.00031327	0.01446943	Adam23	a disintegrin and metallopeptidase domain 23
ENSMUSG0C	1707.11452	0.36576738	0.11878922	3.07912929	0.00207607	0.05294725	Ercc5	excision repair cross-complementing rodent repair deficiency, complementation group 5
ENSMUSG0C	345.233671	0.6426024	0.20775201	3.09312234	0.00198062	0.05139883	Ecrg4	ECRG4 augurin precursor
ENSMUSG0C	2881.98997	0.2725861	0.08105343	3.36304226	0.00077089	0.02756351	Il1r2	interleukin 1 receptor, type II

ENSMUSG0C	1721.51865	0.34845007	0.1029791	3.38369716	0.00071517	0.02616224	Rev1	REV1, DNA directed polymerase
ENSMUSG0C	12559.3505	0.27251296	0.0776004	3.5117467	0.00044517	0.01863409	Ogfrl1	opioid growth factor receptor-like 1
ENSMUSG0C	3422.30728	0.30466338	0.07881205	3.86569565	0.00011077	0.00642963	Wnt10a	wingless-type MMTV integration site family, member 10A
ENSMUSG0C	720.539296	0.76957393	0.12707559	6.05603247	1.40E-09	4.26E-07	Plcd4	phospholipase C, delta 4
ENSMUSG0C	15095.1561	0.24954401	0.08190871	3.04661127	0.00231437	0.05720062	Itm2c	integral membrane protein 2C
ENSMUSG0C	2868.60314	0.89495121	0.13688882	6.53779612	6.24E-11	2.64E-08	Serpine2	serine (or cysteine) peptidase inhibitor, clade E, member 2
ENSMUSG0C	3766.40794	0.23484766	0.07904995	2.97087669	0.00296951	0.06724067	Scly	selenocysteine lyase
ENSMUSG0C	3103.75023	0.2749238	0.08577305	3.2052467	0.00134947	0.04037505	Smyd2	SET and MYND domain containing 2
ENSMUSG0C	2108.17098	0.40074759	0.10830222	3.70027116	0.00021537	0.01077651	Cr2	complement receptor 2
ENSMUSG0C	28026.0071	0.4561416	0.08003573	5.69922429	1.20E-08	2.76E-06	Vim	vimentin
ENSMUSG0C	8319.63946	0.31652409	0.0799791	3.95758511	7.57E-05	0.00484692	Psmb7	proteasome (prosome, macropain) subunit, beta type 7
ENSMUSG0C	6038.41377	0.58453922	0.09080792	6.43709539	1.22E-10	4.93E-08	Il2ra	interleukin 2 receptor, alpha chain
ENSMUSG0C	1141.39305	0.66163337	0.11535642	5.73555749	9.72E-09	2.32E-06	Ak1	adenylate kinase 1
ENSMUSG0C	159.25188	0.8323783	0.28000292	2.9727487	0.00295146	0.06715766	1700007K13I	RIKEN cDNA 1700007K13 gene
ENSMUSG0C	5987.74093	0.55375239	0.08118228	6.82109938	9.03E-12	4.37E-09	Stk39	serine/threonine kinase 39
ENSMUSG0C	2209.12323	0.69043958	0.09620601	7.17667811	7.14E-13	4.78E-10	Cers6	ceramide synthase 6
ENSMUSG0C	1599.84638	3.40842526	0.12017733	28.3616314	6.02E-177	5.24E-173	Abcb11	ATP-binding cassette, sub-family B (MDR/TAP), member 11
ENSMUSG0C	35950.4428	0.2494842	0.07329122	3.40401208	0.00066404	0.02475995	Cd82	CD82 antigen
ENSMUSG0C	521.087972	0.59118627	0.17493703	3.37942331	0.00072638	0.02646123	F2	coagulation factor II
ENSMUSG0C	4231.63125	0.33419867	0.08619796	3.87710672	0.00010571	0.0061975	Pdrg1	p53 and DNA damage regulated 1
ENSMUSG0C	490.761231	0.4643018	0.1635006	2.83975598	0.00451481	0.08974464	Col9a3	collagen, type IX, alpha 3
ENSMUSG0C	1868.21516	0.26692696	0.09324443	2.86265862	0.00420103	0.08555851	Armc1	armadillo repeat containing 1
ENSMUSG0C	3177.87633	0.65568812	0.0934907	7.01340497	2.33E-12	1.27E-09	Zmat3	zinc finger matrin type 3
ENSMUSG0C	118.676606	0.75948632	0.26540439	2.86161927	0.00421483	0.08563922	1810062G17	RIKEN cDNA 1810062G17 gene
ENSMUSG0C	6689.25067	0.39244897	0.11534486	3.40239666	0.00066798	0.02480297	Slc7a11	solute carrier family 7 (cationic amino acid transporter, y+ system), member 11
ENSMUSG0C	133.22768	1.17671948	0.25678928	4.58243231	4.60E-06	0.00051632	Sohlh2	spermatogenesis and oogenesis specific basic helix-loop-helix 2
ENSMUSG0C	5153.3573	0.24821592	0.08577434	2.8938249	0.0038058	0.07996572	Etfdh	electron transferring flavoprotein, dehydrogenase
ENSMUSG0C	191.668214	0.90253022	0.25995036	3.47193295	0.00051673	0.02068446	Hsd3b1	hydroxy-delta-5-steroid dehydrogenase, 3 beta- and steroid delta-isomerase 1
ENSMUSG0C	3951.04196	0.23932718	0.08284159	2.88897366	0.00386501	0.08097129	Gba	glucosidase, beta, acid
ENSMUSG0C	11853.6282	0.2619279	0.08118151	3.22644755	0.00125337	0.03842424	Lmna	lamin A
ENSMUSG0C	1020.52225	0.37393437	0.13005761	2.87514408	0.00403843	0.08322037	Gatb	glutamyl-tRNA(Gln) amidotransferase, subunit B
ENSMUSG0C	1253.0635	0.37046046	0.12310532	3.00929683	0.00261853	0.06186769	Nudt17	nudix (nucleoside diphosphate linked moiety X)-type motif 17
ENSMUSG0C	6222.20922	0.39149491	0.09815992	3.98833751	6.65E-05	0.00440542	Trp53inp1	transformation related protein 53 inducible nuclear protein 1
ENSMUSG0C	531.874068	0.49076788	0.14255377	3.44268603	0.00057597	0.02235329	Smim8	small integral membrane protein 8
ENSMUSG0C	17398.4989	0.25941267	0.07515279	3.45180342	0.00055685	0.02183893	Bspry	B-box and SPRY domain containing
ENSMUSG0C	3900.73834	0.26262729	0.08962725	2.93021702	0.00338725	0.07372781	B4galt1	UDP-Gal:betaGlcNAc beta 1,4- galactosyltransferase, polypeptide 1
ENSMUSG0C	22575.8251	0.24655695	0.07670737	3.21425353	0.00130784	0.03967499	Tpm2	tropomyosin 2, beta
ENSMUSG0C	4958.23015	0.32376243	0.10309733	3.14035724	0.00168742	0.04663973	Glipr2	GLI pathogenesis-related 2
ENSMUSG0C	4243.25979	0.49627056	0.12125275	4.09286018	4.26E-05	0.00302833	Ttc39a	tetratricopeptide repeat domain 39A
ENSMUSG0C	469.491661	0.55158965	0.15007089	3.67552732	0.00023736	0.01164261	Cyp2j13	cytochrome P450, family 2, subfamily j, polypeptide 13
ENSMUSG0C	6303.33578	0.31908377	0.08861932	3.60061173	0.00031747	0.01462459	Tnfrsf8	tumor necrosis factor receptor superfamily, member 8
ENSMUSG0C	2648.567	0.33723107	0.10074274	3.34744792	0.00081559	0.02880715	Tinagl1	tubulointerstitial nephritis antigen-like 1
ENSMUSG0C	5761.26429	0.37731354	0.08056643	4.68326003	2.82E-06	0.00034143	Sesn2	sestrin 2
ENSMUSG0C	8431.90233	0.22210022	0.07297824	3.04337595	0.0023394	0.05737458	Park7	Parkinson disease (autosomal recessive, early onset) 7
ENSMUSG0C	831.699961	0.74198456	0.13328114	5.56706362	2.59E-08	5.37E-06	Abcb1b	ATP-binding cassette, sub-family B (MDR/TAP), member 1B
ENSMUSG0C	1775.11974	0.40347731	0.11180358	3.60880504	0.00030761	0.01425667	Lzic	leucine zipper and CTNNBIP1 domain containing
ENSMUSG0C	9662.76865	0.56363702	0.07438212	7.57758735	3.52E-14	3.23E-11	Plb1	phospholipase B1
ENSMUSG0C	1156.42165	0.43371602	0.11795279	3.67703063	0.00023596	0.01160693	Anxa3	annexin A3
ENSMUSG0C	7998.29663	0.26120471	0.06968864	3.74816774	0.00017813	0.00923154	Arpc1b	actin related protein 2/3 complex, subunit 1B
ENSMUSG0C	4872.06177	0.47306456	0.08702277	5.43610114	5.45E-08	1.08E-05	Exoc4	exocyst complex component 4
ENSMUSG0C	1713.72658	0.30741449	0.10328571	2.97635057	0.00291701	0.06692217	Mrpl19	mitochondrial ribosomal protein L19
ENSMUSG0C	4290.70503	0.38842618	0.08502268	4.56850092	4.91E-06	0.00054482	Tmem43	transmembrane protein 43
ENSMUSG0C	15669.8123	0.34199815	0.0824492	4.14798617	3.35E-05	0.00252837	Usp18	ubiquitin specific peptidase 18
ENSMUSG0C	16052.9843	0.26340985	0.07205302	3.65577829	0.0002564	0.01249699	Aebp2	AE binding protein 2
ENSMUSG0C	169.385414	1.06783509	0.2949086	3.62090182	0.00029358	0.0138915	Slc7a10	solute carrier family 7 (cationic amino acid transporter, y+ system), member 10
ENSMUSG0C	551.336753	0.6115159	0.18687059	3.2724031	0.00106637	0.03503542	Ddias	DNA damage-induced apoptosis suppressor
ENSMUSG0C	229.525402	0.76111131	0.22671411	3.35714133	0.00078753	0.02810089	Art2b	ADP-ribosyltransferase 2b
ENSMUSG0C	1311.80569	0.50613575	0.12856232	3.93689032	8.25E-05	0.00516702	Il21r	interleukin 21 receptor
ENSMUSG0C	85.6211023	0.95605203	0.34002816	2.8116849	0.00492828	0.09473669	Zfp300	zinc finger protein 300
ENSMUSG0C	158.536937	0.68793964	0.23959853	2.8712181	0.00408893	0.08386406	Rab33a	RAB33A, member RAS oncogene family
ENSMUSG0C	3321.17926	0.32554335	0.09772803	3.33111558	0.00086499	0.0300639	Agpat5	1-acylglycerol-3-phosphate O-acyltransferase 5 (lysophosphatidic acid acyltransferase, epsilon)
ENSMUSG0C	2764.59517	0.35478725	0.08993892	3.94475787	7.99E-05	0.00503972	Plpbp	pyridoxal phosphate binding protein
ENSMUSG0C	1849.65581	0.41883293	0.11083343	3.77894028	0.0001575	0.00838512	Il15	interleukin 15
ENSMUSG0C	5765.83544	0.44211353	0.13853988	3.19123663	0.00141665	0.04159741	Ccl22	chemokine (C-C motif) ligand 22
ENSMUSG0C	6565.96341	0.44451061	0.07543527	5.89261007	3.80E-09	1.02E-06	Ces2g	carboxylesterase 2G
ENSMUSG0C	5110.8953	0.25576335	0.07879911	3.24576439	0.00117136	0.03685519	Cfdp1	craniofacial development protein 1
ENSMUSG0C	1678.63585	0.47662822	0.11249209	4.23699339	2.27E-05	0.0018347	Clmp	CXADR-like membrane protein
ENSMUSG0C	2045.79741	0.45913958	0.10433688	4.40054916	1.08E-05	0.0010504	Il10ra	interleukin 10 receptor, alpha
ENSMUSG0C	2331.11271	1.03995645	0.24702526	4.2099194	2.55E-05	0.00203307	Mcam	melanoma cell adhesion molecule
ENSMUSG0C	1517.07091	0.31369512	0.10349293	3.03107776	0.00243682	0.0590159	Tmed1	transmembrane p24 trafficking protein 1
ENSMUSG0C	5619.82316	0.2264926	0.08053776	2.81225358	0.00491957	0.09473669	Cgnl1	cingulin-like 1
ENSMUSG0C	1135.39514	0.30652618	0.1030798	2.97367843	0.00294253	0.06715766	Lca5	Leber congenital amaurosis 5 (human)
ENSMUSG0C	5573.44396	0.57422751	0.09060279	6.33785705	2.33E-10	8.27E-08	Sh3bgrl2	SH3 domain binding glutamic acid-rich protein like 2
ENSMUSG0C	11334.0234	0.24671733	0.07700681	3.20383792	0.00135609	0.04050353	Tspan3	tetraspanin 3
ENSMUSG0C	13310.805	0.42598719	0.09703671	4.38995919	1.13E-05	0.0010847	Col12a1	collagen, type XII, alpha 1
ENSMUSG0C	11954.6704	0.2253433	0.06904483	3.26372469	0.00109958	0.03572198	Stoml1	stomatin-like 1
ENSMUSG0C	523.83657	0.55736651	0.16759768	3.32562193	0.00088222	0.03043526	Cgas	cyclic GMP-AMP synthase
ENSMUSG0C	3898.78936	0.29308114	0.08518088	3.44069155	0.00058023	0.02245231	Plscr1	phospholipid scramblase 1
ENSMUSG0C	6032.18075	0.28575722	0.07028239	4.06584409	4.79E-05	0.00329404	Mras	muscle and microspikes RAS
ENSMUSG0C	33824.7433	0.2575155	0.07898562	3.26028341	0.00111301	0.03595702	Rpsa	ribosomal protein SA
ENSMUSG0C	3578.3624	0.31908942	0.08564942	3.72552933	0.00019491	0.00988915	Slc25a38	solute carrier family 25, member 38
ENSMUSG0C	212.673964	1.22219052	0.30059997	4.06583718	4.79E-05	0.00329404	Lyzl4	lysozyme-like 4
ENSMUSG0C	2360.83333	0.26891619	0.09011718	2.98407254	0.00284439	0.0661274	Mrpl3	mitochondrial ribosomal protein L3
ENSMUSG0C	6376.61595	0.26067571	0.0811361	3.21282039	0.00131438	0.03973503	Col6a4	collagen, type VI, alpha 4
ENSMUSG0C	1188.1037	0.44675581	0.12586994	3.54934466	0.00038619	0.01676995	Alpk2	alpha-kinase 2
ENSMUSG0C	5179.21124	0.286986	0.0928992	3.08921941	0.00200683	0.05178498	Mgll	monoglyceride lipase
ENSMUSG0C	2369.60275	0.32439584	0.1063443	3.05043002	0.00228514	0.05676338	Gstt2	glutathione S-transferase, theta 2
ENSMUSG0C	2105.03987	0.26415316	0.09278996	2.84678606	0.0044163	0.08818932	Tnxb	tenascin XB
ENSMUSG0C	5891.72656	0.32111168	0.11525935	2.78599247	0.00533641	0.09959676	Chst2	carbohydrate sulfotransferase 2
ENSMUSG0C	7038.33733	0.27240248	0.09124746	2.98531578	0.00283286	0.06603553	Atp6v0b	ATPase, H+ transporting, lysosomal V0 subunit B
ENSMUSG0C	473.840269	0.46239604	0.15830824	2.92085895	0.00349068	0.07513372	B3galt2	UDP-Gal:betaGlcNAc beta 1,3-galactosyltransferase, polypeptide 2
ENSMUSG0C	7681.88055	0.33699361	0.08638094	3.90124958	9.57E-05	0.0056873	Poglut1	protein O-glucosyltransferase 1
ENSMUSG0C	10248.7971	0.26789507	0.08852274	3.02628548	0.00247578	0.05964761	Tspoap1	TSPO associated protein 1
ENSMUSG0C	7032.53709	0.38989312	0.0853892	4.56607076	4.97E-06	0.00054768	Cpne2	copine II
ENSMUSG0C	858.893799	0.49714228	0.11872788	4.1872412	2.82E-05	0.00219502	Lif	leukemia inhibitory factor
ENSMUSG0C	5688.08573	0.22765612	0.07348151	3.0981414	0.00194738	0.05106586	Cyb561a3	cytochrome b561 family, member A3
ENSMUSG0C	465.129677	0.61786676	0.19324563	3.1973129	0.00138714	0.04100905	Eda2r	ectodysplasin A2 receptor
ENSMUSG0C	12146.6815	0.26376215	0.08596062	3.06840667	0.00215204	0.05405064	Bmp2k	BMP2 inducible kinase
ENSMUSG0C	1713.72664	0.69282856	0.14463725	4.79011163	1.67E-06	0.00021989	Nostrin	nitric oxide synthase trafficker
ENSMUSG0C	910.979145	0.44452419	0.11635536	3.82040144	0.00013323	0.00745985	Ccdc122	coiled-coil domain containing 122
ENSMUSG0C	1298.98787	0.52453645	0.13842572	3.78929892	0.00015107	0.00822073	Art3	ADP-ribosyltransferase 3
ENSMUSG0C	1582.34561	0.51820014	0.121545	4.26344281	2.01E-05	0.00167716	Ttc21b	tetratricopeptide repeat domain 21B
ENSMUSG0C	2716.64658	0.65196116	0.14134775	4.61246235	3.98E-06	0.00045288	Cobll1	Cobl-like 1
ENSMUSG0C	4295.54912	0.35935319	0.1179874	3.04569121	0.00232146	0.05720062	Tanc1	tetratricopeptide repeat, ankyrin repeat and coiled-coil containing 1
ENSMUSG0C	1234.22026	0.44879717	0.11923149	3.76408246	0.00016716	0.0087411	Thyn1	thymocyte nuclear protein 1
ENSMUSG0C	1005.61068	0.49472211	0.14228811	3.47690419	0.00050724	0.02044573	Tnni3	troponin I, cardiac 3
ENSMUSG0C	3802.89143	0.341873	0.08716699	3.92204685	8.78E-05	0.00536442	Ggta1	glycoprotein galactosyltransferase alpha 1, 3
ENSMUSG0C	20708.2933	0.6903632	0.12485947	5.5291219	3.22E-08	6.59E-06	Acta2	actin alpha 2, smooth muscle, aorta
ENSMUSG0C	8964.8192	0.26336217	0.07958214	3.30931232	0.00093525	0.03174578	Tmem63b	transmembrane protein 63b
ENSMUSG0C	885.90923	0.44268064	0.1331178	3.32548048	0.00088266	0.03043526	Nudt1	nudix (nucleoside diphosphate linked moiety X)-type motif 1
ENSMUSG0C	1817.0916	0.5007186	0.10341741	4.84172461	1.29E-06	0.00017648	Aaas	achalasia, adrenocortical insufficiency, alacrimia
ENSMUSG0C	3947.52294	0.44057467	0.11190117	3.93717652	8.24E-05	0.00516702	Rps27l	ribosomal protein S27-like
ENSMUSG0C	3355.80438	0.3584812	0.11661136	3.07415336	0.00211101	0.05342877	Neto2	neuropilin (NRP) and tolloid (TLL)-like 2
ENSMUSG0C	1535.72006	0.45891468	0.10374449	4.42350906	9.71E-06	0.00096628	Piwil4	piwi-like RNA-mediated gene silencing 4
ENSMUSG0C	1674.11231	0.55778234	0.15433002	3.6142179	0.00030126	0.01406371	Arhgap10	Rho GTPase activating protein 10
ENSMUSG0C	821.850919	0.65872387	0.16745803	3.93366545	8.37E-05	0.00518425	Islr	immunoglobulin superfamily containing leucine-rich repeat

ENSMUSG0C	23784.6161	0.20780886	0.06684499	3.10881739	0.00187838	0.04983062	Clic4	chloride intracellular channel 4 (mitochondrial)
ENSMUSG0C	1221.3388	0.52424736	0.12596074	4.16199028	3.15E-05	0.00239893	Galnt6	polypeptide N-acetylgalactosaminyltransferase 6
ENSMUSG0C	17460.8061	0.20922417	0.07446234	2.80979856	0.00495725	0.09506677	Tap1	transporter 1, ATP-binding cassette, sub-family B (MDR/TAP)
ENSMUSG0C	1480.18432	0.34720697	0.09746424	3.56240367	0.00036747	0.01611798	Nudt22	nudix (nucleoside diphosphate linked moiety X)-type motif 22
ENSMUSG0C	55989.7746	0.30070386	0.06914311	4.34900714	1.37E-05	0.00126666	Icam1	intercellular adhesion molecule 1
ENSMUSG0C	3805.37491	0.42614141	0.07545886	5.64733414	1.63E-08	3.59E-06	Kcnh3	potassium voltage-gated channel, subfamily H (eag-related), member 3
ENSMUSG0C	467.139867	0.5512256	0.19799428	2.78404807	0.00536851	0.09998056	Kcnk2	potassium channel, subfamily K, member 2
ENSMUSG0C	21294.0674	0.2143331	0.06817811	3.14372305	0.00166813	0.04632725	Inf2	inverted formin, FH2 and WH2 domain containing
ENSMUSG0C	3882.46131	0.44283013	0.08409988	5.26552654	1.40E-07	2.41E-05	Cd81	CD81 antigen
ENSMUSG0C	2303.74652	0.37173319	0.09088907	4.0899659	4.31E-05	0.0030539	Ccdc33	coiled-coil domain containing 33
ENSMUSG0C	6043.06543	0.28957381	0.08240573	3.51400076	0.00044141	0.01861089	Tapbpl	TAP binding protein-like
ENSMUSG0C	1307.22254	0.40683724	0.14444314	2.81659091	0.00485363	0.09401142	Asic3	acid-sensing (proton-gated) ion channel 3
ENSMUSG0C	930.686073	1.04101426	0.20813597	5.00160664	5.69E-07	8.68E-05	Atg9b	autophagy related 9B
ENSMUSG0C	947.472847	0.63432711	0.11236065	5.64545615	1.65E-08	3.59E-06	Afg1l	AFG1 like ATPase
ENSMUSG0C	6692.3884	0.36209823	0.09576588	3.78107772	0.00015615	0.00838512	Ankrd35	ankyrin repeat domain 35
ENSMUSG0C	10155.1272	0.29309823	0.07784594	3.76510607	0.00016648	0.00873159	Tfdp1	transcription factor Dp 1
ENSMUSG0C	1377.74629	0.50199623	0.1629567	3.08054975	0.00206619	0.05283192	Gdf15	growth differentiation factor 15
ENSMUSG0C	3208.41441	0.39433098	0.1033273	3.81632898	0.00013545	0.00748769	Sp6	trans-acting transcription factor 6
ENSMUSG0C	7482.49661	0.32159014	0.1004299	3.20213533	0.00136413	0.04060441	Atp5j2	ATP synthase, H+ transporting, mitochondrial F0 complex, subunit F2
ENSMUSG0C	1676.86348	0.32954724	0.10693456	3.08176558	0.00205777	0.052694	Mboat1	membrane bound O-acyltransferase domain containing 1
ENSMUSG0C	3333.85942	0.66450008	0.10037844	6.6199486	3.59E-11	1.60E-08	Ephx1	epoxide hydrolase 1, microsomal
ENSMUSG0C	9369.22389	0.23251415	0.07824398	2.97165562	0.00296199	0.06715766	Stap2	signal transducing adaptor family member 2
ENSMUSG0C	4114.03734	0.27798786	0.08556017	3.24903361	0.00115798	0.03672836	Rabggtb	Rab geranylgeranyl transferase, b subunit
ENSMUSG0C	1924.08996	0.41435567	0.12552812	3.30089915	0.00096376	0.03233501	Igfbp2	insulin-like growth factor binding protein 2
ENSMUSG0C	3429.93402	0.30315131	0.08774224	3.45502118	0.00055025	0.02172675	Orai2	ORAI calcium release-activated calcium modulator 2
ENSMUSG0C	827.568229	0.38785181	0.12996485	2.98428243	0.00284244	0.0661274	Cercam	cerebral endothelial cell adhesion molecule
ENSMUSG0C	1036.77501	0.64300524	0.12086996	5.31981006	1.04E-07	1.88E-05	Xkr5	X-linked Kx blood group related 5
ENSMUSG0C	53.4487247	1.26502973	0.39942725	3.16710924	0.00153962	0.04406462	Rep15	RAB15 effector protein
ENSMUSG0C	961.379318	0.64609951	0.1304766	4.95184185	7.35E-07	0.00010668	Dnm3	dynamin 3
ENSMUSG0C	7702.88134	0.32974868	0.09234334	3.57089819	0.00035576	0.01591028	Ppp1r15a	protein phosphatase 1, regulatory subunit 15A
ENSMUSG0C	2946.37257	0.39058292	0.11795599	3.31125978	0.00092877	0.03158723	Matn1	matrilin 1, cartilage matrix protein
ENSMUSG0C	4520.12336	0.35365532	0.0868792	4.07065556	4.69E-05	0.00325235	Rcsd1	RCSD domain containing 1
ENSMUSG0C	411.942842	1.04882323	0.16498544	6.35706556	2.06E-10	7.67E-08	Slc19a2	solute carrier family 19 (thiamine transporter), member 2
ENSMUSG0C	1192.15015	1.1444331	0.1591166	7.1924178	6.37E-13	4.43E-10	Grid1	glutamate receptor, ionotropic, delta 1
ENSMUSG0C	3496.36519	0.31942143	0.10650585	2.99909744	0.00270781	0.06354588	Plekha6	pleckstrin homology domain containing, family A member 6
ENSMUSG0C	6108.79904	0.34342638	0.09819039	3.49755588	0.00046954	0.01934153	Phlda3	pleckstrin homology like domain, family A, member 3
ENSMUSG0C	482.467416	0.51407393	0.16795626	3.06076065	0.00220776	0.05523511	Rhod	ras homolog family member D
ENSMUSG0C	4090.1176	0.25535415	0.08441987	3.02481079	0.00248789	0.05980575	Slc16a6	solute carrier family 16 (monocarboxylic acid transporters), member 6
ENSMUSG0C	2871.61779	0.35578292	0.09619371	3.69860889	0.00021678	0.01081623	Klhdc8a	kelch domain containing 8A
ENSMUSG0C	51757.3848	0.25212178	0.08965082	2.81226415	0.00491941	0.09473669	S100a14	S100 calcium binding protein A14
ENSMUSG0C	4883.87366	0.35094786	0.09579595	3.66349368	0.0002488	0.01216945	Ikbke	inhibitor of kappaB kinase epsilon
ENSMUSG0C	1820.8734	0.42232146	0.12989654	3.251214	0.00114913	0.03668296	Dusp15	dual specificity phosphatase-like 15
ENSMUSG0C	1911.60577	0.42818386	0.11061163	3.87105632	0.00010836	0.00633207	Atpaf2	ATP synthase mitochondrial F1 complex assembly factor 2
ENSMUSG0C	10238.0033	0.24350211	0.0679308	3.5845611	0.00033765	0.01539116	Tmem18	transmembrane protein 18
ENSMUSG0C	2304.25259	0.35028351	0.11839993	2.95847748	0.00309163	0.06896803	Tuba1c	tubulin, alpha 1C
ENSMUSG0C	2429.80173	0.39912884	0.10314774	3.86948695	0.00010906	0.00635165	Niban3	niban apoptosis regulator 3
ENSMUSG0C	1410.7291	0.70538211	0.11100195	6.354682	2.09E-10	7.67E-08	Rbm20	RNA binding motif protein 20
ENSMUSG0C	1001.57834	0.3454724	0.10624527	3.25164957	0.00114737	0.03668296	Npsr1	neuropeptide S receptor 1
ENSMUSG0C	1730.79594	0.38773769	0.11970477	3.23911651	0.00119901	0.03734935	Tnip3	TNFAIP3 interacting protein 3
ENSMUSG0C	230.951917	0.66481377	0.21907437	3.03464877	0.00240816	0.05864795	Dnd1	DND microRNA-mediated repression inhibitor 1
ENSMUSG0C	532.491298	1.13664949	0.15460046	7.35217413	1.95E-13	1.43E-10	D630023F18	RIKEN cDNA D630023F18 gene
ENSMUSG0C	1937.96879	0.319887	0.09795788	3.26555663	0.00109249	0.03569147	S1pr1	sphingosine-1-phosphate receptor 1
ENSMUSG0C	23556.8183	0.20874179	0.0712684	2.92895293	0.00340106	0.07374744	Rpl18a	ribosomal protein L18A
ENSMUSG0C	341.598476	0.99862237	0.22395978	4.45893613	8.24E-06	0.00083875	E330021D16	RIKEN cDNA E330021D16 gene
ENSMUSG0C	21617.6229	0.39331819	0.07329083	5.36654	8.03E-08	1.52E-05	Sh3tc2	SH3 domain and tetratricopeptide repeats 2
ENSMUSG0C	1210.56993	0.65998654	0.10433263	6.32579226	2.52E-10	8.60E-08	Pqlc3	PQ loop repeat containing
ENSMUSG0C	4407.30379	0.3016329	0.09760103	3.09046825	0.00199841	0.0517063	Igfals	insulin-like growth factor binding protein, acid labile subunit
ENSMUSG0C	3987.31903	0.67110262	0.10503284	6.38945504	1.66E-10	6.44E-08	Lxn	latexin
ENSMUSG0C	21710.3849	0.21042256	0.07296308	2.88395942	0.00392709	0.08165632	Syngr2	synaptogyrin 2
ENSMUSG0C	693.524105	1.09320057	0.14871804	7.3508269	1.97E-13	1.43E-10	Inka2	inka box actin regulator 2
ENSMUSG0C	746.321367	0.4839699	0.15689663	3.08464181	0.00203797	0.05247672	Tenm2	teneurin transmembrane protein 2
ENSMUSG0C	83290.3275	0.37656147	0.08916477	4.22320895	2.41E-05	0.00193267	Tmsb4x	thymosin, beta 4, X chromosome
ENSMUSG0C	3177.54256	0.33802824	0.09184244	3.6805234	0.00023276	0.01148152	Ptges	prostaglandin E synthase
ENSMUSG0C	542.360706	0.41666329	0.13553309	3.07425498	0.00211029	0.05342877	Tatdn1	TatD DNase domain containing 1
ENSMUSG0C	10432.6065	0.32412736	0.08396363	3.86033036	0.00011323	0.00655063	Gja1	gap junction protein, alpha 1
ENSMUSG0C	253.035465	0.61425161	0.20596087	2.98237044	0.00286026	0.06634016	Serpinb1b	serine (or cysteine) peptidase inhibitor, clade B, member 1b
ENSMUSG0C	307.782768	0.64272914	0.19672745	3.26710458	0.00108654	0.03556361	Cox6b2	cytochrome c oxidase subunit 6B2
ENSMUSG0C	660.140091	0.79721176	0.1694151	4.70567122	2.53E-06	0.00031472	E230016K23	RIKEN cDNA E230016K23 gene
ENSMUSG0C	1028.67196	0.42597414	0.11431925	3.72618028	0.0001944	0.00988915	Tg	thyroglobulin
ENSMUSG0C	1042.68461	0.53359966	0.13167519	4.05239328	5.07E-05	0.00344834	Sh2d4a	SH2 domain containing 4A
ENSMUSG0C	3015.28125	0.43575848	0.11477212	3.79672776	0.00014662	0.00801715	Krt15	keratin 15
ENSMUSG0C	2670.50453	0.28898757	0.10368317	2.78721771	0.00531627	0.0995401	Maf	avian musculoaponeurotic fibrosarcoma oncogene homolog
ENSMUSG0C	1109.15591	0.32536529	0.11581699	2.80930531	0.00496485	0.09510781	Nod2	nucleotide-binding oligomerization domain containing 2
ENSMUSG0C	1808.13241	0.26917164	0.09448066	2.84896037	0.00438623	0.08796053	Cars2	cysteinyl-tRNA synthetase 2 (mitochondrial)(putative)
ENSMUSG0C	310.302177	0.76222618	0.21538918	3.53883232	0.0004019	0.01732253	Uts2b	urotensin 2B
ENSMUSG0C	1134.11873	0.40405438	0.10643371	3.79630067	0.00014687	0.00801715	Drc3	dynein regulatory complex subunit 3
ENSMUSG0C	10140.696	0.35460231	0.08118581	4.36778656	1.26E-05	0.00117502	Capg	capping protein (actin filament), gelsolin-like
ENSMUSG0C	423.08435	0.59335094	0.18635087	3.18405242	0.00145229	0.04200777	Glipr1	GLI pathogenesis-related 1 (glioma)
ENSMUSG0C	3189.00247	0.44624056	0.0905757	4.92671407	8.36E-07	0.00012034	St6galnac2	ST6 (alpha-N-acetyl-neuraminyl-2,3-beta-galactosyl-1,3)-N-acetylgalactosaminide alpha-2,6-sialyltransferase 2
ENSMUSG0C	15551.4874	0.2334624	0.07586165	3.07747611	0.00208762	0.05301569	Birc2	baculoviral IAP repeat-containing 2
ENSMUSG0C	795.625084	0.4801942	0.14430469	3.32764104	0.00087585	0.0303807	Bloc1s2	biogenesis of lysosomal organelles complex-1, subunit 2
ENSMUSG0C	13561.5654	0.25906843	0.08969222	2.88841573	0.00387188	0.08097129	Colq	collagen-like tail subunit (single strand of homotrimer) of asymmetric acetylcholinesterase
ENSMUSG0C	3565.03847	0.26524102	0.09342893	2.83896032	0.00452608	0.08986614	Zfp809	zinc finger protein 809
ENSMUSG0C	1284.64747	0.73571037	0.15135985	4.86067066	1.17E-06	0.00016168	Adtrp	androgen dependent TFPI regulating protein
ENSMUSG0C	479.647065	0.58314699	0.20431438	2.8541652	0.00431501	0.0869644	Gstp3	glutathione S-transferase pi 3
ENSMUSG0C	1610.51367	0.73258477	0.12863109	5.69523863	1.23E-08	2.79E-06	Serpina9	serine (or cysteine) peptidase inhibitor, clade A (alpha-1 antiproteinase, antitrypsin), member 9
ENSMUSG0C	18017.4913	0.23390419	0.07076856	3.30519919	0.00094909	0.03202808	Rpl5	ribosomal protein L5
ENSMUSG0C	680.530711	0.56963349	0.20232465	2.81544288	0.00487101	0.0942148	Fcer1g	Fc receptor, IgE, high affinity I, gamma polypeptide
ENSMUSG0C	266.809243	0.54185243	0.19110347	2.83538762	0.00457701	0.09046481	Rad51b	RAD51 paralog B
ENSMUSG0C	332.630966	0.92601659	0.21330017	4.34137764	1.42E-05	0.00130452	Actg2	actin, gamma 2, smooth muscle, enteric
ENSMUSG0C	11324.3073	0.26397399	0.07594779	3.47572956	0.00050947	0.02048805	Ptk2b	PTK2 protein tyrosine kinase 2 beta
ENSMUSG0C	7211.95701	0.32885073	0.10117166	3.25042331	0.00115233	0.03668296	Tnip2	TNFAIP3 interacting protein 2
ENSMUSG0C	2690.58167	0.75211742	0.08599572	8.74598703	2.21E-18	3.85E-15	Ptp4a3	protein tyrosine phosphatase 4a3
ENSMUSG0C	39559.2599	0.25674194	0.08719687	2.94439392	0.00323588	0.07114438	H2-Q7	histocompatibility 2, Q region locus 7
ENSMUSG0C	8303.91913	0.29309406	0.08893091	3.2957502	0.00098159	0.03261922	Blnk	B cell linker
ENSMUSG0C	278590.206	0.21088086	0.07490841	2.81518266	0.00487495	0.0942148	H2-K1	histocompatibility 2, K1, K region
ENSMUSG0C	1275.95972	0.45169431	0.16125932	2.80104307	0.00509377	0.09725642	Akap6	A kinase (PRKA) anchor protein 6
ENSMUSG0C	2753.55075	0.50156062	0.12742543	3.93611102	8.28E-05	0.00516702	Dsg1b	desmoglein 1 beta
ENSMUSG0C	5190.14355	0.30328286	0.07661077	3.95874982	7.53E-05	0.00484114	Ei24	etoposide induced 2.4 mRNA
ENSMUSG0C	1255.11458	0.58328567	0.12972459	4.49633843	6.91E-06	0.00072085	Ces2f	carboxylesterase 2F
ENSMUSG0C	300.838242	1.02822204	0.23798803	4.32047803	1.56E-05	0.00139029	Plppr1	phospholipid phosphatase related 1
ENSMUSG0C	1327.364	0.42056345	0.12755921	3.29700584	0.00097721	0.03261922	Arhgap22	Rho GTPase activating protein 22
ENSMUSG0C	1481.76931	0.3713611	0.11529908	3.22085055	0.00127811	0.03904507	Slc24a3	solute carrier family 24 (sodium/potassium/calcium exchanger), member 3
ENSMUSG0C	5450.72365	0.26737581	0.08631512	3.09767042	0.00195048	0.05106586	Ifi27	interferon, alpha-inducible protein 27
ENSMUSG0C	120200.915	0.4209795	0.12331843	3.41375978	0.00064073	0.02414943	ND1	NADH dehydrogenase subunit 1
ENSMUSG0C	81301.9581	0.52100722	0.12685608	4.10707328	4.01E-05	0.00291944	ND2	NADH dehydrogenase subunit 2
ENSMUSG0C	326444.506	0.41822261	0.12608582	3.31696773	0.00091	0.0311891	COX1	cytochrome c oxidase subunit I
ENSMUSG0C	105014.819	0.34398625	0.10910791	3.15271588	0.00161759	0.04543084	ND5	NADH dehydrogenase subunit 5
ENSMUSG0C	305454.647	0.5176894	0.12559153	4.12200874	3.76E-05	0.0027712	CYTB	cytochrome b
ENSMUSG0C	952.381258	0.40099558	0.13507857	2.96860999	0.0029915	0.06765064	Cyp2b19	cytochrome P450, family 2, subfamily b, polypeptide 19
ENSMUSG0C	4191.77067	0.2758103	0.08582339	3.21369638	0.00131038	0.03968291	Vsig10	V-set and immunoglobulin domain containing 10
ENSMUSG0C	2714.88352	0.53093031	0.16157172	3.2860349	0.00101608	0.0335732	Myl9	myosin, light polypeptide 9, regulatory
ENSMUSG0C	1520.99889	1.25054499	0.09436227	13.2525952	4.36E-40	2.53E-36	Psrc1	proline/serine-rich coiled-coil 1
ENSMUSG0C	1559.51761	0.30969823	0.11099779	2.79012964	0.00526869	0.09891409	Urm1	ubiquitin related modifier 1
ENSMUSG0C	18343.9573	0.27075275	0.06820494	3.96969422	7.20E-05	0.00467584	Marcks	myristoylated alanine rich protein kinase C substrate
ENSMUSG0C	162.28567	0.8452356	0.25643533	3.29609651	0.00098038	0.03261922	Zfp820	zinc finger protein 820

ENSMUSG0C	1821.34367	0.49895827	0.14559746	3.42697106	0.00061035	0.0234099	Fcho1	FCH domain only 1
ENSMUSG0C	3332.34579	0.28202762	0.08411415	3.35291547	0.00079965	0.02847511	Sp110	Sp110 nuclear body protein
ENSMUSG0C	57.8460696	1.2253263	0.3755268	3.26295301	0.00110258	0.03575268	Olfr155	olfactory receptor 155
ENSMUSG0C	40978.1778	0.24944203	0.07178743	3.47473125	0.00051137	0.02051706	H2-Q6	histocompatibility 2, Q region locus 6
ENSMUSG0C	3438.41267	0.33853867	0.10745945	3.15038527	0.00163055	0.04559307	Pappa2	pappalysin 2
ENSMUSG0C	1260.68282	0.80795542	0.13823613	5.84474862	5.07E-09	1.28E-06	Gm5532	predicted gene 5532
ENSMUSG0C	162.337213	0.73973919	0.23188223	3.19015037	0.00142199	0.04159741	1700066B19	RIKEN cDNA 1700066B19 gene
ENSMUSG0C	42713.0254	0.24092513	0.07426971	3.24392173	0.00117896	0.03698965	Il4i1	interleukin 4 induced 1
ENSMUSG0C	360.774443	0.58073043	0.16061492	3.61566915	0.00029957	0.01404203	4930579C12	RIKEN cDNA 4930579C12 gene
ENSMUSG0C	24744.9693	0.26121005	0.08194603	3.18758633	0.00143466	0.04163612	S100a16	S100 calcium binding protein A16
ENSMUSG0C	660.904052	0.55543101	0.14545984	3.81844923	0.00013429	0.00747108	Foxs1	forkhead box S1
ENSMUSG0C	275.890667	1.33568972	0.21930259	6.09062432	1.12E-09	3.56E-07	Ano3	anoctamin 3
ENSMUSG0C	7815.26237	0.4470813	0.08721703	5.12607817	2.96E-07	4.77E-05	Cd80	CD80 antigen
ENSMUSG0C	2363.93374	0.82386324	0.12260481	6.71966499	1.82E-11	8.57E-09	Scn9a	sodium channel, voltage-gated, type IX, alpha
ENSMUSG0C	1558.0509	0.51975155	0.13080769	3.97340209	7.09E-05	0.00462086	Rprm	reprimo, TP53 dependent G2 arrest mediator candidate
ENSMUSG0C	1247.22855	0.3124632	0.10562366	2.95826896	0.00309372	0.06896803	Sarnp	SAP domain containing ribonucleoprotein
ENSMUSG0C	232.91097	0.74703927	0.21214816	3.52130922	0.00042942	0.01814931	Zfp268	zinc finger protein 268
ENSMUSG0C	283.142326	1.01718794	0.2091491	4.8634584	1.15E-06	0.00016069	Dact3	dishevelled-binding antagonist of beta-catenin 3
ENSMUSG0C	2544.03994	0.72114899	0.11035065	6.53506808	6.36E-11	2.64E-08	Col22a1	collagen, type XXII, alpha 1
ENSMUSG0C	19653.3258	0.32539693	0.09130995	3.56365242	0.00036573	0.01611798	Psme2	proteasome (prosome, macropain) activator subunit 2 (PA28 beta)
ENSMUSG0C	12116.0589	0.44821063	0.0883796	5.07142632	3.95E-07	6.14E-05	Pglyrp2	peptidoglycan recognition protein 2
ENSMUSG0C	3670.6854	0.27571272	0.0947244	2.91068325	0.00360639	0.07705294	Eloc	elongin C
ENSMUSG0C	232.624681	0.96634408	0.32492259	2.97407474	0.00293873	0.06715766	NA	NA
ENSMUSG0C	127.725294	1.10084505	0.27129207	4.05778554	4.95E-05	0.00339624	AI847159	expressed sequence AI847159
ENSMUSG0C	694.656351	0.42736082	0.12652844	3.37758714	0.00073125	0.02652754	Slc2a4rg-ps	Slc2a4 regulator, pseudogene
ENSMUSG0C	974.614103	0.79973811	0.15269872	5.23735951	1.63E-07	2.73E-05	Snhg15	small nucleolar RNA host gene 15
ENSMUSG0C	4101.36852	0.25973564	0.08474869	3.06477485	0.00217834	0.05457758	NA	NA
ENSMUSG0C	2647.00435	0.76538619	0.09264389	8.26159416	1.44E-16	1.79E-13	Slc13a2os	solute carrier family 13 (sodium-dependent dicarboxylate transporter), member 2, opposite strand
ENSMUSG0C	136.311476	1.28830647	0.25376485	5.07677266	3.84E-07	6.02E-05	Trp53cor1	tumor protein p53 pathway corepressor 1
ENSMUSG0C	84.6487882	0.89422381	0.30343594	2.94699372	0.0032088	0.07081722	NA	NA
ENSMUSG0C	118.566424	0.98807471	0.30349209	3.25568518	0.00113119	0.03636146	A930001C03	RIKEN cDNA A930001C03 gene
ENSMUSG0C	329.84203	0.72644992	0.2355307	3.08431093	0.00204024	0.05247672	Pou3f1	POU domain, class 3, transcription factor 1
ENSMUSG0C	964.296717	0.56807862	0.13144422	4.32182266	1.55E-05	0.00138896	Tcerg1l	transcription elongation regulator 1-like
ENSMUSG0C	1262.54436	0.30779901	0.11027776	2.79112511	0.00525252	0.09891409	1110038B12	RIKEN cDNA 1110038B12 gene
ENSMUSG0C	328.865373	1.05802462	0.2073805	5.10185213	3.36E-07	5.32E-05	1700092C10	RIKEN cDNA 1700092C10 gene
ENSMUSG0C	426.538006	0.67473247	0.16123611	4.18474784	2.85E-05	0.00219961	Art2a	ADP-ribosyltransferase 2a
ENSMUSG0C	2394.52405	0.27397596	0.09701084	2.82417874	0.00474019	0.09243113	Serpinb10	serine (or cysteine) peptidase inhibitor, clade B (ovalbumin), member 10
ENSMUSG0C	13293.0517	0.31332725	0.10561849	2.96659484	0.00301117	0.06774365	Rpl41	ribosomal protein L41
ENSMUSG0C	3612.26106	0.27873562	0.08743748	3.18782757	0.00143346	0.04163612	S100a2	S100 calcium binding protein A2
ENSMUSG0C	15304.1207	0.39112333	0.08092612	4.83309149	1.34E-06	0.00018288	NA	NA
ENSMUSG0C	3040.58035	0.54797446	0.12092789	4.53141485	5.86E-06	0.00063368	Ptprv	protein tyrosine phosphatase, receptor type, V
ENSMUSG0C	1144.17063	0.44669711	0.14266489	3.13109362	0.00174157	0.04716313	Bcl2a1d	B cell leukemia/lymphoma 2 related protein A1d
ENSMUSG0C	3132.24028	0.3663043	0.12797663	2.86227491	0.00420612	0.08556209	NA	NA
ENSMUSG0C	609.01218	0.41645611	0.13771098	3.0241315	0.00249348	0.05980575	NA	NA
ENSMUSG0C	777.750592	0.41873943	0.1378811	3.03696039	0.00238977	0.05829398	NA	NA
ENSMUSG0C	2086.27939	0.42609454	0.11841234	3.59839635	0.00032019	0.01471079	NA	NA
ENSMUSG0C	170.833351	0.84976664	0.24821601	3.42349647	0.00061821	0.02360724	NA	NA
ENSMUSG0C	984.438368	0.99006698	0.33798163	2.92935146	0.0033967	0.07374744	NA	NA
ENSMUSG0C	240.030646	0.72727609	0.21448084	3.39086743	0.00069672	0.02554095	NA	NA
ENSMUSG0C	161.64821	0.74001387	0.25300757	2.92486855	0.00344602	0.07444852	NA	NA
ENSMUSG0C	1840.06533	0.49087164	0.12783985	3.83973879	0.00012317	0.0069407	NA	NA
ENSMUSG0C	526.055862	0.56253061	0.15709852	3.58075049	0.00034261	0.01541562	Gm32815	predicted gene, 32815
ENSMUSG0C	178.278618	0.88182939	0.28205129	3.1264859	0.00176909	0.04775994	2900057B20	RIKEN cDNA 2900057B20 gene
ENSMUSG0C	186.722322	0.92067346	0.21655583	4.25143686	2.12E-05	0.00176123	NA	NA
ENSMUSG0C	477.906206	0.40086994	0.14381454	2.78740901	0.00531314	0.0995401	A930007A09	RIKEN cDNA A930007A09 gene

Table 5. GO Downregulated in cKO cTEC

id	term	inPopulation	inStudySet	estimate	std.error	items
GO:0021562	vestibulococl	8	3	0.630868	0.012551	ENSMUSG00000025969; ENSMUSG00000027950; ENSMUSG00000045871
GO:0071636	positive regu	19	5	0.57294	0.03062055	ENSMUSG00000015957; ENSMUSG00000030530; ENSMUSG00000032487; ENSMUSG00000036446; ENSMUSG00000067001
GO:0061669	spontaneous	9	3	0.522488	0.01575685	ENSMUSG00000035864; ENSMUSG00000037386; ENSMUSG00000052301
GO:0032532	regulation of	6	3	0.432208	0.02883883	ENSMUSG00000025497; ENSMUSG00000026175; ENSMUSG00000034918
GO:1901570	fatty acid de	60	7	0.40268	0.01725937	ENSMUSG00000018796; ENSMUSG00000025701; ENSMUSG00000026335; ENSMUSG00000029919; ENSMUSG00000032487; ENSMUSG00000047250; ENSMUSG00000060063
GO:1901021	positive regu	41	5	0.29478	0.01376317	ENSMUSG00000006930; ENSMUSG00000019370; ENSMUSG00000026576; ENSMUSG00000034330; ENSMUSG00000040118
GO:0048710	regulation of	32	4	0.263372	0.01238944	ENSMUSG00000003436; ENSMUSG00000033326; ENSMUSG00000036634; ENSMUSG00000038539
GO:0048712	negative regı	17	3	0.228728	0.02559467	ENSMUSG00000003436; ENSMUSG00000033326; ENSMUSG00000038539
GO:1903515	calcium ion t	2	2	0.228412	0.00952399	ENSMUSG00000020788; ENSMUSG00000029467
GO:0046461	neutral lipid	32	4	0.200284	0.00918372	ENSMUSG00000020573; ENSMUSG00000032080; ENSMUSG00000034171; ENSMUSG00000039202
GO:0032528	microvillus o	25	4	0.18494	0.01640636	ENSMUSG00000025497; ENSMUSG00000026175; ENSMUSG00000028943; ENSMUSG00000034918
GO:0032914	positive regu	7	3	0.181428	0.01902413	ENSMUSG00000030530; ENSMUSG00000036446; ENSMUSG00000067001
GO:0046464	acylglycerol (	32	4	0.158756	0.01636517	ENSMUSG00000020573; ENSMUSG00000032080; ENSMUSG00000034171; ENSMUSG00000039202
GO:1905867	epididymis d	5	2	0.157036	0.0128256	ENSMUSG00000004266; ENSMUSG00000046352
GO:0046967	cytosol to en	4	2	0.145408	0.01344832	ENSMUSG00000020788; ENSMUSG00000029467
GO:0051452	intracellular	36	4	0.142164	0.00822518	ENSMUSG00000031883; ENSMUSG00000032245; ENSMUSG00000037242; ENSMUSG00000038023
GO:0007035	vacuolar acid	20	3	0.137568	0.01044827	ENSMUSG00000032245; ENSMUSG00000037242; ENSMUSG00000038023
GO:0098814	spontaneous	15	3	0.136004	0.00376617	ENSMUSG00000035864; ENSMUSG00000037386; ENSMUSG00000052301
GO:1903279	regulation of	3	2	0.129832	0.00554275	ENSMUSG00000007097; ENSMUSG00000026576
GO:0120316	sperm flagel	19	3	0.128928	0.01028344	ENSMUSG00000006435; ENSMUSG00000022783; ENSMUSG00000047021
GO:0046503	glycerolipid (	51	5	0.126076	0.00861516	ENSMUSG00000020573; ENSMUSG00000022425; ENSMUSG00000032080; ENSMUSG00000034171; ENSMUSG00000039202
GO:0007288	sperm axone	19	3	0.123424	0.00782094	ENSMUSG00000006435; ENSMUSG00000022783; ENSMUSG00000047021
GO:0030046	parallel actir	6	2	0.117652	0.00539875	ENSMUSG00000010154; ENSMUSG00000028943
GO:0045742	positive regu	28	4	0.1136	0.01167347	ENSMUSG00000006435; ENSMUSG00000006930; ENSMUSG00000031980; ENSMUSG00000033717
GO:0032536	regulation of	12	3	0.109972	0.0117723	ENSMUSG00000025497; ENSMUSG00000026175; ENSMUSG00000034918
GO:0002540	leukotriene p	3	2	0.100676	0.00595281	ENSMUSG00000025701; ENSMUSG00000060063

Table 6. GO Upregulated in cKO cTEC

id	term	inPopulation	inStudySet	estimate	std.error	items
GO:0002181	cytoplasmic t	96	16	1	0	ENSMUSG00000007892; ENSMUSG00000017404; ENSMUSG00000033554; ENSMUSG00000034892; ENSMUSG00000037805; ENSMUSG00000039001; ENSMUSG00000047215; ENSMUSG00000049517; ENSMUSG00000057322; ENSMUSG00000057841; ENSMUSG00000057863; ENSMUSG00000059291; ENSMUSG00000060636; ENSMUSG00000060938; ENSMUSG00000067288; ENSMUSG00000079641
GO:0042274	ribosomal sm	63	11	0.984084	0.00435238	ENSMUSG00000009927; ENSMUSG00000025290; ENSMUSG00000036781; ENSMUSG00000037563; ENSMUSG00000039001; ENSMUSG00000047675; ENSMUSG00000057322; ENSMUSG00000061477; ENSMUSG00000063457; ENSMUSG00000067288; ENSMUSG00000090733
GO:0042273	ribosomal lar	69	11	0.853292	0.00511053	ENSMUSG00000005846; ENSMUSG00000008167; ENSMUSG00000025794; ENSMUSG00000038900; ENSMUSG00000043716; ENSMUSG00000057322; ENSMUSG00000058558; ENSMUSG00000059291; ENSMUSG00000060636; ENSMUSG00000060938; ENSMUSG00000062997
GO:0042773	ATP synthesi	59	8	0.502352	0.02389553	ENSMUSG00000038302; ENSMUSG00000041881; ENSMUSG00000059534; ENSMUSG00000064345; ENSMUSG00000064351; ENSMUSG00000064363; ENSMUSG00000064367; ENSMUSG00000064370
GO:0019646	aerobic elect	45	7	0.422072	0.02159802	ENSMUSG00000038302; ENSMUSG00000041881; ENSMUSG00000059534; ENSMUSG00000064345; ENSMUSG00000064351; ENSMUSG00000064363; ENSMUSG00000064370

Table 7. GO Downregulated in cKO mTEC

id	term	inPopulation	inStudySet	estimate	std.error	items
GO:1904057	negative reg	3	3	0.206852	0.02040512	ENSMUSG00000016529; ENSMUSG00000027895; ENSMUSG00000037362
GO:0071625	vocalization	19	6	0.1717	0.02960254	ENSMUSG00000024109; ENSMUSG00000028351; ENSMUSG00000030739; ENSMUSG00000031253; ENSMUSG00000037541; ENSMUSG00000038738
GO:0060074	synapse mat	29	7	0.121604	0.0360469	ENSMUSG00000000632; ENSMUSG00000006435; ENSMUSG00000022055; ENSMUSG00000024109; ENSMUSG00000030683; ENSMUSG00000038738; ENSMUSG00000058153
GO:0060412	ventricular s	38	8	0.118564	0.03581177	ENSMUSG00000010175; ENSMUSG00000020644; ENSMUSG00000031558; ENSMUSG00000032744; ENSMUSG00000039910; ENSMUSG00000056427; ENSMUSG00000057123; ENSMUSG00000063632

Table 8. GO Upregulated in cKO mTEC

id	term	inPopulation	inStudySet	estimate	std.error	items
GO:0008630	intrinsic apo	104	11	0.979104	0.00694728	ENSMUSG00000003873; ENSMUSG00000005087; ENSMUSG00000006445; ENSMUSG00000022037; ENSMUSG00000023067; ENSMUSG00000032369; ENSMUSG00000036781; ENSMUSG00000041801; ENSMUSG00000042349; ENSMUSG00000062762; ENSMUSG00000099974
GO:0010499	proteasomal	21	5	0.94258	0.00541975	ENSMUSG00000015671; ENSMUSG00000018286; ENSMUSG00000021024; ENSMUSG00000024338; ENSMUSG00000026750
GO:0002474	antigen proc	20	5	0.650944	0.01865091	ENSMUSG00000037321; ENSMUSG00000038213; ENSMUSG00000058715; ENSMUSG00000060550; ENSMUSG00000061232
GO:0042104	positive regu	24	4	0.650616	0.01957691	ENSMUSG00000000732; ENSMUSG00000020053; ENSMUSG00000026770; ENSMUSG00000039323
GO:0042119	neutrophil ac	28	6	0.466008	0.02662026	ENSMUSG00000021457; ENSMUSG00000021678; ENSMUSG00000025279; ENSMUSG00000029484; ENSMUSG00000031712; ENSMUSG00000058715
GO:1903318	negative reg	35	5	0.461056	0.02988771	ENSMUSG00000016256; ENSMUSG00000020184; ENSMUSG00000026073; ENSMUSG00000026249; ENSMUSG00000057367
GO:0051459	regulation of	9	3	0.402196	0.02560588	ENSMUSG00000021680; ENSMUSG00000026051; ENSMUSG00000034394
GO:0010955	negative reg	35	5	0.367084	0.02005999	ENSMUSG00000016256; ENSMUSG00000020184; ENSMUSG00000026073; ENSMUSG00000026249; ENSMUSG00000057367
GO:0051497	negative reg	30	4	0.303788	0.01587286	ENSMUSG00000009376; ENSMUSG00000018293; ENSMUSG00000032232; ENSMUSG00000045092
GO:0051458	corticotropin	11	3	0.300964	0.03527856	ENSMUSG00000021680; ENSMUSG00000026051; ENSMUSG00000034394
GO:0019882	antigen proc	103	11	0.220484	0.01780813	ENSMUSG00000024338; ENSMUSG00000031104; ENSMUSG00000037321; ENSMUSG00000037405; ENSMUSG00000038213; ENSMUSG00000055994; ENSMUSG00000058715; ENSMUSG00000060550; ENSMUSG00000061232; ENSMUSG00000073409; ENSMUSG00000079197
GO:0002446	neutrophil m	29	5	0.201468	0.01860846	ENSMUSG00000021457; ENSMUSG00000021678; ENSMUSG00000025279; ENSMUSG00000027249; ENSMUSG00000029484
GO:2000479	regulation of	19	3	0.189408	0.00357906	ENSMUSG00000020826; ENSMUSG00000028893; ENSMUSG00000061603
GO:0006749	glutathione r	57	6	0.18934	0.00825447	ENSMUSG00000001663; ENSMUSG00000027737; ENSMUSG00000028964; ENSMUSG00000033318; ENSMUSG00000050737; ENSMUSG00000058216
GO:0019677	NAD cataboli	4	2	0.163744	0.01103615	ENSMUSG00000028100; ENSMUSG00000030651
GO:0090131	mesenchyme	5	2	0.150904	0.01030295	ENSMUSG00000035783; ENSMUSG00000059430
GO:0006521	regulation of	11	3	0.145828	0.00800367	ENSMUSG00000000958; ENSMUSG00000027737; ENSMUSG00000028964
GO:0042276	error-prone t	6	2	0.140008	0.00413767	ENSMUSG00000021668; ENSMUSG00000026082
GO:0042773	ATP synthesi	59	6	0.128484	0.00630264	ENSMUSG00000028964; ENSMUSG00000038302; ENSMUSG00000064345; ENSMUSG00000064351; ENSMUSG00000064367; ENSMUSG00000064370
GO:0018120	peptidyl-argi	5	2	0.12332	0.00754271	ENSMUSG00000030651; ENSMUSG00000034842
GO:0015990	electron tran	4	2	0.11802	0.00682092	ENSMUSG00000064351; ENSMUSG00000064370
GO:0007253	cytoplasmic :	6	2	0.113916	0.00434986	ENSMUSG00000021025; ENSMUSG00000049775
GO:0032232	negative reg	35	4	0.103308	0.00331504	ENSMUSG00000009376; ENSMUSG00000018293; ENSMUSG00000032232; ENSMUSG00000045092
GO:1903999	negative reg	6	2	0.101224	0.01004241	ENSMUSG00000034848; ENSMUSG00000043659
